# Fracture healing requires proliferation of callus cells at early and mature stages of osteoblast differentiation

**DOI:** 10.1172/jci.insight.196751

**Published:** 2026-09-08

**Authors:** Nicole R. Gould, Andre F. Coello, Jennifer A. McKenzie, Mariam Obaji, Tiandao Li, Katherine R. Hixon, Leyi Chen, Kristen Barwick, Tiffany Lee, Bo Zhang, David Ornitz, Matthew J. Silva

**Affiliations:** 1 Department of Orthopaedic Surgery,; 2 Musculoskeletal Research Center,; 3 Department of Biomedical Engineering, and; 4 Department of Developmental Biology, Washington University in St. Louis, St. Louis, Missouri, USA.; 5 Thayer School of Engineering, Dartmouth College, Hanover, New Hampshire, USA.; 6 Department of Mechanical Engineering and Materials Science, Washington University in St. Louis, St. Louis, Missouri, USA.

**Keywords:** Bone biology, Cell biology, Mouse models, Orthopedics, Osteoclast/osteoblast biology

## Abstract

Fractures heal by rapid formation of mineralized callus, a process requiring periosteal cell proliferation and differentiation. Our objective was to dissect the contribution of proliferating osteoblast-lineage cells to fracture callus formation. First, mice expressing thymidine kinase (TK) in 3.6Col1a1-lineage cells were treated with ganciclovir (GCV) to ablate proliferating osteolineage cells for 5 or 10 days. Immunostaining demonstrated that this approach specifically depleted TK^+^ proliferating cells in the bony regions of the callus, while sparing other proliferating cells. Single-cell RNA-seq of callus cells revealed that GCV-treated Col1-TK mice had fewer osteoblasts and chondrocytes than controls, with more myofibroblasts and immune cells, consistent with fibrous nonunion. In controls, 15%–30% of callus cells expressing the early osteoblast marker Osterix (Osx, *Sp7*) and the late marker osteocalcin (Ocn, *Bglap*) were in the cell cycle. Next, we targeted proliferating osteolineage cells at different stages of differentiation by crossing Osx-CreERT2, Ocn-Cre, and Dmp1-CreERT2 mice with ROSA-TK mice. Following fracture, each Cre ROSA-TK mouse line exhibited decreased callus bone volume and a shift from callus bone to fibrous tissue. Therefore, during fracture repair, proliferation of callus cells at early and mature stages of osteoblast differentiation is critical to the formation of a mineralized callus that is essential for healing.

## Abstract 2

<div> Following fracture, bones heal via the formation of mineralized callus. We determined that proliferation of osteoblasts is essential for callus formation essential to fracture healing. </div>

## Graphical abstract

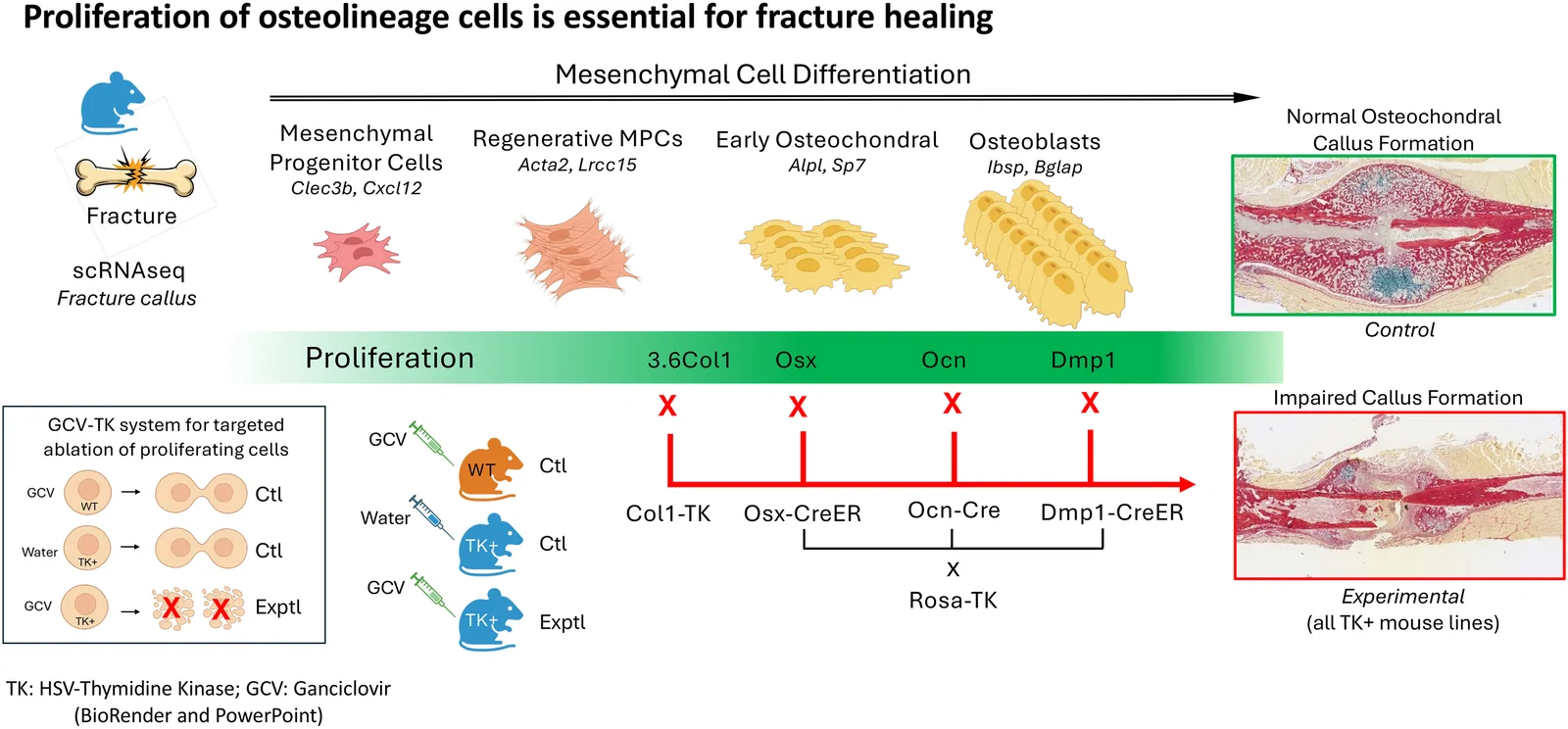

## Introduction

Healing of broken bones occurs via a regenerative process involving multiple cell types and overlapping stages of inflammation, fibrovascular callus formation, intramembranous and endochondral ossification, and bone remodeling (1, 2). Functionally, long bone healing is successful when a periosteal mineralized callus bridges the fracture gap with sufficient stiffness and strength to allow weight bearing. Failed healing leads to fracture nonunion, characterized by inadequate osteochondral tissue and persistence of fibrovascular tissue (3, 4). In recent years, our understanding of cell types and molecular factors that contribute to fracture healing has increased greatly with the use of mouse genetic and lineage-tracing models. In particular, the contribution to callus formation of different skeletal stem and progenitor cell populations has been described (5–11).

Among the cell types essential for fracture healing is the osteoblast. Bone-forming osteoblasts are needed in great number for mineralized callus formation, and rapid expansion of this population must involve proliferation of osteoprogenitors and/or more differentiated cells. Proliferation of periosteal cells is a histological hallmark of early fracture healing (12–14). However, the molecular identity of these proliferating cells is not well described. In particular, the differentiation stage(s) of proliferating osteolineage cells in the fracture callus is unknown. Osteolineage cell proliferation in fracture healing is often assumed to occur at the progenitor stage (1, 2, 7), consistent with the view of the mature osteoblast as a postmitotic cell (7, 15–17). This view is supported by classic in vitro studies (15, 18) and histological observations of cells in developing animals (19, 20), but has not been critically examined in the context of fracture healing.

We recently studied the role of osteolineage cell proliferation in fracture healing using the Col1-TK mouse, in which herpes simplex virus (HSV) thymidine kinase (TK) expression is driven by the 3.6 kb fragment of the *Col1a1* promoter (21). Treatment with ganciclovir (GCV), a nucleoside analog that incorporates into the DNA of replicating cells and is converted to a toxic form by HSV-TK, leads to ablation of proliferating 3.6Col1a1-lineage cells, which in turn results in minimal callus formation and fracture nonunion (22). This finding demonstrated that proliferation of osteolineage cells is essential to fracture healing. But because the *3.6Col1a1* promoter is active in both pre- and mature osteoblasts and periosteal fibroblasts (23), it remains unclear at what stage(s) of differentiation osteolineage cells in the fracture callus are proliferating to contribute to fracture healing.

Here we characterize the cellular composition of the periosteal callus in the first 5–10 days after fracture in normal-healing mice and in Col1-TK non-healers, with the goal of identifying the contribution of osteolineage cell proliferation to mineralized callus formation. Using single-cell mRNA sequencing (scRNA-seq), we identify a shift from osteochondral cells to fibroblasts in normal versus non-healing mice. Using a newly generated Cre-inducible ROSA-TK mouse model, we show that proliferation of cells in the fracture callus at both early (Osx^+^) and mature (Ocn^+^, Dmp1^+^) stages of osteoblast differentiation is essential to fracture healing.

## Results

### Ablation of proliferating 3.6Col1a1-lineage cells for the first 7 or 14 days after fracture impairs callus bone formation.

When GCV is administered to Col1-TK mice, HSV-TK expressed in 3.6Col1a1-lineage cells converts GCV into a toxic form, which incorporates into the DNA of replicating cells to induce apoptosis (21). We recently reported that GCV dosing for 14 or 28 days following fracture in Col1-TK mice results in failure of mineralized callus formation, establishing the first 2 weeks as a critical time for osteogenic cell proliferation (22). To extend the prior work, we treated WT control and TK^+^ experimental mice with GCV for 3, 7, or 14 days and evaluated fracture healing at 21 days post-fracture (DPF) (Figure 1A). Based on radiographic analysis, the periosteal fracture callus in 90%–100% of control mice was fully bridged by 21 DPF, regardless of GCV dosing duration (Figure 1B and Supplemental Figure 1A; supplemental material available online with this article; https://doi.org/10.1172/jci.insight.196751DS1). Experimental mice treated with GCV for 3 days had delayed callus formation compared with control, but by 21 DPF 100% had a fully bridged callus. In contrast, experimental mice treated with GCV for 7 or 14 days had significantly impaired bridging, with only 57% and 14% of samples fully bridged, respectively.

Consistent with radiographic findings, micro-CT of fracture callus showed that experimental mice treated with GCV for 7 or 14 days had progressively less bone volume at 21 DPF compared with controls, while 3 days of GCV did not alter callus bone volume (Figure 1C). Other micro-CT parameters also showed a significant interaction between GCV duration and genotype, with longer treatment durations impairing callus formation in experimental but not control mice (Supplemental Figure 1B). Notably, the parameter most affected by 3 days of GCV dosing was total volume (–20%, TK vs. WT, *P* < 0.05), indicating that targeting proliferation for the first 3 days of fracture healing reduces eventual callus size at 21 DPF.

Histological analysis also demonstrated progressively smaller callus size and altered tissue composition in experimental mice with longer GCV dosing (Figure 1D and Supplemental Figure 1C). At 21 DPF, in both control and experimental mice treated with GCV for 3 days, callus composition was 95% woven bone, indicating that healing had largely progressed past the cartilage stage. In contrast, GCV treatment for 7 or 14 days in experimental mice significantly altered callus composition, with woven bone constituting 70% and 20% of the callus, respectively, and fibrous tissue constituting 8% and 45% of the callus, respectively. Notably, WT mice treated with GCV had normal healing (as previously reported) (22), and we saw no evidence that bone cells away from the fracture site (e.g., cortical osteocytes, bone lining cells) were affected by GCV treatment in control or TK^+^ mice (Supplemental Figure 1D). In summary, targeted ablation of proliferating osteoblast-lineage cells for the first 3 days after fracture resulted in a modest, transient delay in healing, while ablation for 7 days led to moderate impairment in callus size and composition at 21 DPF, and ablation for 14 days resulted in a small fracture callus with markedly less bone and more fibrous tissue. This indicates that proliferation of 3.6Col1a1-lineage cells throughout the first 2 weeks after fracture contributes to callus formation.

### Five-day ablation of proliferating 3.6Col1a1-lineage cells depletes fracture callus of osteoblasts and chondrocytes.

As ablation of proliferating 3.6Col1a1-lineage cells for 7 or 14 days after fracture caused persistent impairment in callus size and composition, we sought to describe the transcriptional and histological changes in the early callus. After fracture, Col1-TK mice were treated for 5 days with either water (Control) or GCV (Experimental) (Figure 2A). Periosteal whole-callus tissue was dissected and enzymatically digested, and the resulting cell samples were profiled using scRNA-seq. For analysis, Control and Experimental samples were integrated before clustering to facilitate comparisons between groups. Unbiased cluster analysis revealed that the early fracture callus contained multiple cell types of 5 major lineages, consistent with recent reports (10, 11). The majority (88%) were mesenchymal (*Prrx1^+^Col1a1^hi^*, *Myh11^–^*), immune (*Ptprc*/CD45^+^), or endothelial (*Pecam1^+^*) (Figure 2B and Supplemental Figure 2A). Smaller populations of smooth muscle (*Myh11^+^*) and skeletal muscle (*Myod1^+^*) were also present, the latter likely from contaminant tissue. (See Supplemental Results for a detailed description of scRNA-seq data.) Notably, chondrocytes and pre-osteoblasts were the only cell types depleted in Experimental mice; these cells (cluster 5) constituted 12% of all callus cells in Control mice, but only 1% in Experimental samples (Supplemental Figure 2B). This result is consistent with specific ablation of proliferating osteochondral cells in 3.6Col1a1-TK mice.

To enhance resolution of the mesenchymal population, cells of the original mesenchymal clusters (0, 1, 4, 5, and 17) were selected in silico for reclustering. Analysis of this mesenchymal subpopulation identified 11 clusters; we assigned cell identities to each cluster based on canonical gene expression and bioinformatic analysis of differentially expressed genes (Figure 2C, Supplemental Figure 2C, and Supplemental Table 1). Overall, the mesenchymal clusters strongly expressed multiple collagen and other extracellular matrix (ECM) formation and remodeling transcripts, including *Col1a1*, *Col3a1*, *Fn1*, *Mmp2*, and *Timp1* (Supplemental Figure 2D), consistent with active synthesis and remodeling of the early callus matrix (10). Clusters were composed predominantly of a diverse population of fibroblastic cells (clusters 0, 1, 2, 3, 6, 8, and 9; 77% of cells) as well as osteochondral cells (clusters 4 and 5; 16%) (Figure 2C). The fibroblastic clusters included mesenchymal progenitors (clusters 6, 1, and 2). Cluster 6 was the least differentiated, marked specifically by expression of *Clec3b* and *Cd34* (Figure 2D and Supplemental Figure 2C), as well as *Ly6a* and *Pi16* (not shown); a similar population of “mesenchymal progenitor cells” (MPCs) in the early callus has been reported by others (9, 11). Cluster 0 contained myofibroblastic cells, marked by the DEGs *Acta2*, *Actg2*, and *Lrrc15*; these appear similar to the regenerative MPCs described by Wang et al. (11).

Clusters 4 and 5 contained the most differentiated of the mesenchymal cells. Cluster 4 was composed of early osteochondral cells, marked by expression of *Alpl*, *Sp7*, *Pth1r*, and *Inhba*. A subset of cells in cluster 4 expressed markers of mature osteoblasts (*Ibsp*, *Bglap*). Cluster 5 was composed of early and mature chondrocytes, based on expression of the cartilage matrix genes *Hapln1*, *Col2a1*, *Snorc*, and *Matn3*. A subset of cells in this cluster also expressed the hypertrophic chondrocyte markers *Col10a1* and *Ihh*. Notably, GCV treatment of Col1-TK mice caused a significant reduction in the number of cells in these two clusters, from 25% of all cells in Control mice to 4% of cells in Experimental mice (Figure 2E). To assess whether GCV treatment targeted proliferating cells, we counted the number of cells in the cell cycle (S or G_2_/M phase) in the Control and Experimental groups (Supplemental Figure 2F). The greatest reductions in proliferating cells with GCV treatment were observed in clusters 4 and 5, confirming the targeted ablation of proliferating osteochondral cells. On the other hand, the myofibroblastic cells of cluster 0 were significantly enriched in the experimental fracture callus (Control, 14%; Experimental, 27%). Also enriched was cluster 8, which was a small population of cells that expressed nonspecific fibroblast markers (*Col1a1*, *Col3a1*, *Fn1*) and specifically expressed innate immune response genes, including *Isg15* and *Ifit1*. Notably, the abundance of callus mesenchymal progenitors (clusters 6, 1, 2) was unaffected by experimental treatment, indicating that the loss of mature osteochondral cells in the callus is not due to progenitor depletion.

We also performed histological analysis at 5 DPF, when the periosteal callus is composed mainly of fibrous/undifferentiated tissue, with small amounts of woven bone and early cartilage. Callus area did not differ between Control and Experimental groups, but callus composition was altered; callus tissue from Experimental mice had less woven bone and cartilage with more fibrous and periosteal tissue (Figure 2F and Supplemental Figure 2G). Immunostaining for the proliferation marker proliferating cell nuclear antigen (PCNA) showed abundant PCNA^+^ cells throughout the callus of Control (water-treated) TK^+^ mice (Supplemental Figure 2H). By contrast, immunostaining for HSV-TK showed TK^+^ cells limited to regions of new periosteal woven bone (Figure 2G and Supplemental Figure 2H). Quantitative analysis confirmed that PCNA^+^ cells were approximately 6-fold more numerous than TK^+^ cells, indicating that TK was expressed only in a subset of proliferating cells (Supplemental Figure 2I). These assays also illustrated the loss of TK^+^ cells in calluses of Experimental (GCV-treated Col1-TK) mice compared with Control mice, most notably in areas of woven bone. By contrast, the number of PCNA^+^ cells in the experimental callus was not diminished, indicating that GCV dosing did not blunt the proliferation of TK^–^ cells in Col1-TK mice.

In summary, scRNA-seq and histology indicate that targeted ablation of proliferating 3.6Col1a1-lineage cells in the first 5 days after fracture deprives the callus of osteoblasts and chondrocytes, resulting in less cartilage and bone formation and a shift toward less differentiated fibrous tissue.

### Ten-day ablation of proliferating 3.6Col1a1-lineage cells depletes fracture callus of osteoblasts and chondrocytes, and causes a shift toward fibroblasts and fibrous tissue.

We next analyzed the callus at 10 DPF, when osteochondral cells and tissue are normally predominant (Figure 3A). scRNA-seq of total callus again revealed a mixed population of cells from 5 lineages, with 90% being either mesenchymal, immune, or endothelial, and the remainder being smooth or skeletal muscle (Figure 3B and Supplemental Figure 3A). From day 5 to day 10, callus cell composition shifted with a large increase in mesenchymal cells (from 46% to 69%), reflecting maturation of callus ECM. As was observed on day 5, callus osteochondral cells on day 10 were significantly depleted by GCV treatment. In calluses from Control mice, 33% of all cells were in clusters 1 (chondrocytes) and 2 (hypertrophic chondrocytes), whereas in Experimental mice, only 9% of cells were in these 2 clusters (Supplemental Figure 3B). The opposite shift was seen in cells in clusters 5 (smooth muscle cells) and 6 (mesenchymal progenitors), which were enriched in Experimental mice (21% of all cells) compared with Control (4.4%). Notably, in Control mice, the proportion of cells in immune clusters declined significantly from day 5 to day 10 (5 DPF, 31%; 10 DPF, 8.7%; *P* = 0.04; Figure 3C), indicating substantial resolution of the early immune cell response. In contrast, in the Experimental group, the decline in immune cells was more modest, from 33% at 5 DPF to 19% at 10 DPF (*P* = 0.19), suggesting a delay in resolution of the immune response.

To enhance resolution of the mesenchymal populations, cells in original clusters 0, 1, 2, 4, 6, 8, 9, and 11 (Figure 3B) were reclustered, resulting in 12 clusters mainly composed of chondrocytes (clusters 0, 2, 3, 6, 8), fibroblastic cells (clusters 1, 4, 7, 9), and osteoblasts (cluster 5) (Figure 3, D and E, and Supplemental Figure 3C). The most notable change in the mesenchymal populations from day 5 to day 10 was an increased proportion of cells in chondrocyte clusters from 16% (day 5) to 55% (day 10). There was a complementary decline in cells in fibroblastic clusters from 77% (day 5) to 34% (day 10).

Similarly to results from day 5, treatment with GCV markedly depleted osteochondral cells in the day 10 callus. Mature and hypertrophic chondrocytes in clusters 0 and 2 were reduced from 39% of mesenchymal cells in Control mice to 15% of cells in Experimental mice (Figure 3F). Similarly, osteoblastic cells (*Sp7^+^*, *Bglap^+^*, *Ibsp^+^*) were reduced from 11% of cells in Control mice to 3.8% in Experimental mice. Moreover, clusters 0, 2, 5, and 6, which were composed of mature and hypertrophic chondrocytes and osteoblasts, had the greatest reduction in number of cells in the cell cycle (Supplemental Figure 3D). In contrast to the reduction in mature osteochondral cells, the proportion of *Clec3b^+^* mesenchymal progenitors (cluster 7) increased from 2.6% in Control mice to 11% in Experimental. Moreover, there was an increase in the proportion of cells in cluster 4, from 2.6% to 21%. This fibroblastic cluster was marked by matrix-producing genes, including *Tnn*, *Postn*, and *Aspn*, and appeared to be at the transition from mesenchymal progenitors to mature osteochondral cells, similarly to the so-called Osteo-X cells observed in the periosteum of intact bones by Nookaew et al. (24). In summary, scRNA-seq indicates that ablation of proliferating 3.6Col1a1-lineage cells in the first 10 DPF leads to depletion of osteochondral cells and relative enrichment of progenitors, as well as persistence of immune cells.

Analysis of micro-CT at 10 DPF revealed that callus bone volume was reduced by 60% in Experimental mice compared with Control (Figure 3G and Supplemental Figure 3E), while histological analysis revealed 50% smaller callus area (Figure 3H). Callus composition was also affected, with calluses from Experimental mice having less woven bone, but more fibrous and periosteal tissue (Supplemental Figure 3F). Immunostaining illustrated strong TK and PCNA staining localized to woven bone regions of callus in Control mice, whereas Experimental mice had markedly less TK-stained woven bone, with a shift in PCNA staining toward regions of non-mineralized tissue (Figure 3I and Supplemental Figure 3G). Quantitative analysis confirmed significantly fewer TK^+^ cells in Experimental callus (*P* = 0.038) (Supplemental Figure 3H). Thus, scRNA-seq and histological analyses indicate that ablation of proliferating 3.6Col1a1-lineage cells in the first 10 days after fracture specifically depletes cartilage and bone cells in the fracture callus, with a shift toward increased fibroblasts and fibrous tissue.

### Development of a ROSA-TK mouse to drive cell-specific TK expression.

Results using Col1-TK mice, in which the *3.6Col1a1* promoter drives HSV-TK expression, show an essential role for proliferation of osteogenic cells during fracture healing, which suggests that osteoblastic cells in fracture callus are not postmitotic. But a limitation of this finding is that the *3.6Col1a1* promoter is active in early osteoblasts and periosteal fibroblasts (23). To specifically target proliferating osteoblasts at different stages of differentiation, we generated a Cre-inducible mouse. A ROSA26-targeting vector was used to knock in an expression cassette containing the HSV-TK gene downstream of a floxed stop cassette (ROSA26-LSL-HSV-TK, or ROSA-TK) (Supplemental Methods, Supplemental Figure 4, and Supplemental Figure 5, A–C). Validation of this model was done by crossing of ROSA-TK mice with Osx-CreERT2 mice, which targets early and mature osteoblasts (25, 26). We confirmed tamoxifen-inducible (TMX-inducible), Cre-mediated excision of the stop cassette and bone-specific TK expression (Supplemental Figure 5, D–F).

### Ablation of proliferating Osx-CreERT2–expressing cells impairs fracture callus formation, phenocopying results from Col1-TK mice.

To further examine the role of proliferating early and mature osteoblasts during bone repair, we assessed fracture healing in Osx-CreERT2 ROSA-TK mice. Cre was activated 2 weeks before fracture by treatment of mice with TMX (Figure 4A). Because Osterix (Osx) expression promotes pre-osteoblast proliferation (27) and because *Osx* and *3.6Col1a1* promoters are active in similar cell types, we hypothesized that Osx-CreERT2 ROSA-TK mice would have a phenotype similar to that of Col1-TK mice. We first assessed HSV-TK expression by immunostaining and confirmed that Cre^–^TK^+^ mice (treated with GCV) did not express TK in the callus at 14 DPF, whereas Cre^+^TK^+^ mice (treated with water to allow normal healing) had strong TK expression in osteoblasts in the woven bone regions of the callus (Figure 4B), consistent with findings in Osx-CreERT2 TdT reporter mice (26). Subsequent experiments included 3 genotype controls (Cre^–^TK^–^, Cre^–^TK^+^, and Cre^+^TK^–^) in addition to experimental (Cre^+^TK^+^) mice, all treated with TMX and GCV (except where noted). Fracture healing was evaluated 2 weeks post-fracture, when the normal callus includes both woven bone and cartilage, but little fibrous tissue.

Radiographic analysis indicated that 89% of Cre^–^ control mice had a fully bridged fracture callus, whereas only 33% of Osx-CreERT2 ROSA-TK were fully bridged (Figure 4C). Histologically, fracture calluses from each control group were of similar size and were composed predominantly of woven bone, with a small amount of cartilage and no fibrous tissue. In contrast, fracture calluses from experimental mice were substantially smaller than control, with less woven bone and more fibrous tissue (Figure 4D and Supplemental Figure 6A). Micro-CT analysis showed that the calluses from each control group were similarly sized, whereas experimental mice had smaller calluses with less callus bone volume (Figure 4E and Supplemental Figure 6B). Measures of bone morphology at the mid-diaphysis of contralateral, intact femora were not significantly different between control and experimental mice, indicating that short-term ablation of proliferating Osx-expressing cells does not alter bone structure (Supplemental Figure 6C). Notable changes in callus size and composition only occurred in Cre^+^TK^+^ mice treated with both TMX and GCV (Supplemental Figure 6, D and E). In all, targeting early osteoblasts in Osx-CreERT2 ROSA-TK mice considerably impaired fracture callus bone formation and phenocopied results from Col1-TK mice. These results support the use of the ROSA-TK mouse to target proliferating cells in a Cre-dependent manner.

### Sp7/Osx-expressing cells proliferate throughout early fracture healing.

The impaired callus formation in Osx-CreERT2 ROSA-TK mice indicates that proliferation of Osx-expressing cells is essential for fracture healing. To better characterize cell proliferation during healing, cell cycle stages were assigned to the scRNA-seq data using described gene markers (28). Approximately 20% of all mesenchymal cells were in the cell cycle (S or G_2_/M phase) at both 5 and 10 DPF (Figure 5A), indicating that even 10 days after fracture, when the callus is well patterned, there is abundant cell proliferation. Of cells with high *Sp7* gene expression (≥90% of *Sp7* mean value), about 30% were in the cell cycle at 5 DPF, and 20% at 10 DPF (Figure 5B). Thus, based on their transcriptome, many *Sp7*-expressing cells proliferate during fracture healing.

We next performed immunofluorescence (IF) staining of callus sections from control mice to visualize expression of PCNA (which is expressed during S phase) (29, 30) and Osx (Figure 5C). PCNA was broadly expressed in cells of the early fracture callus; approximately 40% of cells at 3 DPF had PCNA^+^ nuclei, while about 20% of cells were PCNA^+^ at 5 and 10 DPF (Figure 5D). Notably, approximately 50% of Osx^+^ cells in fracture callus were also PCNA^+^ at 3 DPF, and the proportion of Osx^+^PCNA^+^ cells remained relatively high (~20%) at 5 and 10 DPF. Osx^+^PCNA^+^ cells were observed throughout the callus, specifically among osteoblast-like cells on new woven bone surfaces (Figure 5C) and among chondrocyte-like cells in cartilage regions (Supplemental Figure 7). Thus, by scRNA-seq and IF we find evidence that a substantial proportion of proliferating cells express Osx, consistent with the functional impairment in callus formation when these cells are ablated in Osx-CreERT2 ROSA-TK mice.

### Proliferation of osteocalcin-lineage cells is necessary for fracture healing.

As osteoblasts mature and initiate matrix mineralization, they express osteocalcin (Ocn) (31) and are said to exit the cell cycle (16). Thus, we hypothesized that there is negligible proliferation of Ocn^+^ cells during fracture healing and thus little contribution of these cells to callus formation. To test this hypothesis, we crossed ROSA-TK mice with Ocn-Cre mice to drive TK expression in mature osteoblasts, and subjected Ocn-Cre ROSA-TK mice and their genotype controls to femur fracture (Figure 6A). Immunostaining confirmed that TK was strongly expressed in the woven bone callus of Cre^+^TK^+^ mice (treated with water), but not Cre^–^TK^+^ mice (Figure 6B). We also observed some TK^+^ cells in the cartilage of the callus. In mice treated with GCV for 14 days after fracture, radiographic analysis showed that 77% of genotype controls had full bridging of mineralized callus, compared with only 33% of experimental mice (Figure 6C). Histologically, callus area was significantly reduced in experimental mice and callus composition was altered, with less woven bone and more fibrous tissue in Cre^+^TK^+^ mice compared with controls (Figure 6D and Supplemental Figure 8A). By micro-CT analysis, callus bone volume and other measures of callus morphology at 14 DPF were significantly diminished in Cre^+^TK^+^ mice compared with controls, whereas bone morphology in non-fractured contralateral femora was normal (Figure 6E and Supplemental Figure 8, B–D). Moreover, we observed no histological evidence of off-target effects at sites away from the fracture (Supplemental Figure 8E).

To assess whether impaired healing of Ocn-Cre ROSA-TK mice at 14 DPF could recover with time, we evaluated healing 12 weeks after fracture, i.e., 10 weeks after stopping GCV treatment. We observed radiographically and histologically that callus formation in Cre^+^TK^+^ mice remained impaired, with incomplete bridging and persistence of fibrous tissue (Supplemental Figure 9, A and B). Because the impaired fracture healing of Ocn-Cre ROSA-TK mice was unexpected, we examined healing at earlier time points. In Cre^–^TK^+^ control mice, callus area increased progressively from 3 to 5 to 10 days, with increasing woven bone and cartilage and decreasing fibrous tissue, consistent with normal healing (Supplemental Figure 9, C and D). In contrast, in Cre^+^TK^+^ experimental mice, callus size and composition did not change over time. Thus, ablation of proliferating Ocn-lineage cells consequentially impairs early callus formation, resulting in a fibrotic callus phenotype that persists even after withdrawal of GCV, indicating that proliferation of callus cells expressing this mature osteoblast marker is required for normal fracture healing.

### Bglap/Ocn-expressing cells proliferate during fracture healing.

To characterize the proliferative state of mature osteoblasts in fracture callus, we queried the scRNA-seq data for cell cycle status of cells expressing *Bglap*, the gene that encodes Ocn. At 5 and 10 DPF, 15%–20% of *Bglap^+^* cells were in the cell cycle (Figure 7A). While the proportion of *Bglap^+^* cells in the cell cycle did not change from 5 to 10 days, the total number of *Bglap^+^* cells increased over 25-fold from 5 DPF (72 cells) to 10 DPF (1,933 cells), indicating a large increase in the number of *Bglap*-expressing cells that were proliferating. We next visualized Ocn and PCNA expression in sections of fracture callus from control mice (Figure 7B). Similar to results from Osx-CreERT2 ROSA-TK mice (Figure 5D), at 3 DPF approximately 40% of all callus cells had PCNA^+^ nuclei, a rate that decreased to approximately 20% at 5 and 10 DPF (Figure 7C). Ocn^+^PCNA^+^ cells were visualized on new woven bone surfaces (Figure 7B) and in chondrocyte-like cells in cartilage regions (Supplemental Figure 10). Of cells that were Ocn^+^ at 3 DPF, 40% also expressed PCNA; at 5 and 10 DPF, 20% were Ocn^+^PCNA^+^. Thus, a substantial proportion of Ocn^+^ cells proliferate early in fracture healing. Taken together, the functional and expression data support that Ocn-expressing cells have the ability to proliferate after fracture and that this function is vital for callus formation.

### Proliferation of Dmp1-expressing cells is necessary for fracture callus bridging and mineralization.

While the Ocn-Cre mouse is widely used to target mature osteoblasts (32), a limitation is that Cre is constitutively active and thus TK will be expressed in all Ocn-lineage cells, not only cells expressing Ocn during fracture repair. In addition, Ocn-Cre may target chondrocytes during fracture healing (Figure 6B and Supplemental Figure 10). Therefore, we crossed ROSA-TK mice with Dmp1-CreERT2 mice to induce TK expression in cells expressing the late-stage osteoblast and osteocyte marker Dmp1, at the time of fracture (Figure 8A). We confirmed strong TK expression in osteoblasts and osteocytes but not chondrocytes during fracture healing (Figure 8B), consistent with a prior report (26). Dmp1-CreERT2 ROSA-TK mice treated with GCV had significantly impaired healing compared with Cre^–^ controls, based on radiographic, histological, and micro-CT analyses (Figure 8, C–E). Thus, ablation of proliferating Dmp1-expressing cells during fracture healing leads to a failure of mineralized callus formation with an increase in fibrous tissue, comparable to results seen when cells were targeted using *3.6Col1a1*, *Osx*, and *Ocn* promoters.

### Impaired fracture healing in ROSA-TK mice is not due to a gap junction–mediated bystander effect.

A feature of the TK/GCV system that has been noted in some contexts is a gap junction–mediated bystander effect, whereby TK-phosphorylated GCV can be passed from TK-expressing cells to non-TK-expressing neighbors, thus inducing apoptosis indirectly (33, 34). To test whether this effect contributes to impaired fracture healing in ROSA-TK mice, we treated Osx-CreERT2 ROSA-TK, Ocn-Cre ROSA-TK, and Dmp1-CreERT2 ROSA-TK mice and their respective controls with the gap junction blocker carbenoxolone (CBX) once daily (along with GCV) following femur fracture. Micro-CT analysis at 14 DPF showed significantly diminished callus bone volume in Cre^+^TK^+^ mice, regardless of CBX treatment (Supplemental Figure 11 and Supplemental Results). In sum, treatment with a gap junction inhibitor did not rescue impaired fracture healing in any of the ROSA-TK models, and thus we find no evidence of a bystander effect.

### Ablation of proliferating adiponectin-expressing cells modestly impacts fracture callus formation.

Because the 3 lines of Cre ROSA-TK mice tested above had impaired callus formation, we asked whether there is an inherent defect in fracture healing in Cre^+^ROSA-TK^+^ mice treated with GCV. To address this, we evaluated fracture healing in Adq-CreERT2 ROSA-TK mice (Supplemental Figure 12A). Adiponectin (*Adq*) is expressed by marrow but not periosteal osteo-progenitors (35), and thus we hypothesized that fracture healing (predominantly a periosteal phenomenon in our model) would be minimally affected in these mice. In support of the hypothesis, we observed a modest impairment in radiographic healing and non-significant reductions in callus size and callus bone volume in Adq-Cre^+^ROSA-TK^+^ mice, while callus composition and bone mineral density were unaffected (Supplemental Figure 12, B–D, and Supplemental Results). Thus, we conclude that there is not an inherent defect in fracture healing in Cre^+^ROSA-TK^+^ mice.

## Discussion

Our results reveal that proliferation of osteoblast-lineage cells at early and later stages of differentiation is critical to formation of a mineralized callus during fracture healing. Proliferation of periosteal cells is a hallmark of early callus formation (12–14), yet the identity of these proliferating cells, apart from their histological appearance, has not been well documented. Analysis of scRNA-seq and immunofluorescence, as well as functional results of the targeting of proliferating cells in TK-expressing mice, demonstrates that callus cells expressing molecular markers of early (3.6Col1a1^+^, Osx^+^) and mature (Ocn^+^, Dmp1^+^) osteoblasts proliferate in the first 10 days following fracture. Based on these findings, a working model is presented (Supplemental Figure 13). In brief, after fracture, mesenchymal progenitor cells (MPCs) differentiate through several stages, including regenerative MPCs, early osteochondral cells, and osteoblasts. Cell proliferation occurs along this path to produce the large numbers of cells needed at each stage of callus formation and maturation. Ablation of proliferating cells that express 3.6Col1, Osx, Ocn, or Dmp1 leads to impaired callus formation. Thus, proliferation of cells at each of these osteoblast lineage stages is essential to fracture repair.

Osteoblasts are often described as postmitotic (7, 16, 17), a view supported by in vitro (15, 18) and observational in vivo studies of the periosteum early in skeletal development. In classic studies that focused on rapid appositional bone growth in embryonic and 2-week-old animals, cell proliferation was commonly observed in pre-osteoblasts (adjacent to but not on the bone surface), whereas proliferation of mature (surface) osteoblasts was rare (19, 20). Thus, proliferation of pre-osteoblasts, followed by their differentiation, appears to be the major source of matrix-synthesizing periosteal osteoblasts early in the developing skeleton. On the other hand, studies in older mice have found evidence that osteoblasts can proliferate. Corral et al. (36) showed that ablation of proliferating Ocn^+^ osteoblasts in 6- to 10-week-old mice caused profound depletion of endosteal osteoblasts and reduced bone formation. Using lineage tracing in 5-month-old mice, we previously reported that up to 20% of Osx^+^ and Dmp1^+^ periosteal cells proliferate after mechanical loading and become incorporated into newly formed lamellar and woven bone (37, 38). Of direct relevance to the current study, Matthews et al. (6) used an inducible 2.3Col1 reporter to show that approximately 10% of osteoblasts in fracture callus derive from preexisting 2.3Col1-expressing cells. This result is consistent with our recent report that Osx^+^ bone surface cells pulse-labeled before fracture contribute to osteoblasts and osteocytes in fracture callus woven bone (26). Taken together with the current findings, these studies support a role for osteoblast proliferation as a source of bone-forming osteoblasts in the postnatal skeleton, especially during fracture repair.

The finding that proliferation of osteoblast-lineage cells is vital to the regenerative process of fracture healing is consistent with observations in other organs, wherein replication of mature tissue-resident cells contributes to regeneration after injury. Following partial liver resection, “virtually all” hepatocytes in the remaining lobes proliferate to restore liver mass within 7 days (in rodents), a process that does not normally require stem/progenitor cells (39). And following partial pancreatectomy, proliferation of mature pancreatic β cells is the major source for new β cells during regeneration (40). Thus, the proliferation of cells expressing mature tissue markers can occur during injury repair, indicating that tissue regeneration does not necessarily depend solely on expansion of progenitor cells. We note that our results do not rule out the possibility that committed osteoblasts first “de-differentiate” before reentering the cell cycle (41); additional studies of fracture healing are needed to test this idea.

Our results using the Col1-TK mouse provide insight into the critical window for osteolineage cell proliferation during fracture repair. Ablating proliferating 3.6Col1a1-lineage cells for the first 3–5 days after fracture led to a transient reduction in callus woven bone and increase in fibrous tissue (Supplemental Figure 2G), although this effect was recovered by 21 days (Figure 1, C and D). On the other hand, ablating these cells for the first 7 days after fracture led to impaired callus composition that persisted on day 21. (A limitation of our study is that we did not determine whether the impairment caused by 7 days of ablation might recover by a later time point.) We had previously established that ablating proliferating 3.6Col1a1-lineage cells for the first 14 days after fracture led to impaired healing at 12 weeks, meeting the time point criterion for nonunion in a rodent model (22, 42). Here, we observed a similar finding in Ocn-Cre ROSA-TK mice (Supplemental Figure 9, A and B). These findings demonstrate that proliferation of osteolineage cells occurs throughout the first 2 weeks of fracture healing, which suggests that this population is transient and needs to be replenished by cells not initially committed to the osteoblast lineage.

There are other clinical implications to our findings. Diminished proliferation of periosteal cells contributes to poor fracture healing with aging (13, 43, 44). Cancer chemotherapy agents act by inhibiting cell proliferation and have been shown to impact bone healing (45–47). Blocking the proliferation of osteolineage cells as a source of new osteoblasts results in a callus composed primarily of fibrous tissue, with an appearance and function consistent with clinical nonunion (22). In patients, the causes and radiographic features of nonunion are diverse (48, 49). Nonetheless, a common histological feature in human nonunion tissue is vascularized fibrous tissue wherein fibroblast-like cells are the predominant cell type, regardless of whether the nonunion is considered atrophic or hypertrophic (3, 4). Interestingly, fibrotic nonunion is also reported in mice with systemic inflammation (50) and local radiation injury (51), suggesting that fibrous tissue is a common outcome in nonunion, regardless of the underlying mechanism.

One of the early stages of fracture healing has been described as the fibrovascular phase, wherein the callus contains “undefined fibrous cells” (1) or “undifferentiated mesenchymal cells” (2). With recent advances in scRNA-seq, the identity and fate of diverse populations of fibroblastic cells in fracture callus have become clearer. Yao et al. (9) identified a population of “proliferative progenitor cells (PPCs)” that appear to represent an intermediate differentiation stage between mesenchymal progenitors (MPCs) and osteoblasts/chondrocytes in the early (day 5) fracture callus of normal-healing mice. Wang et al. (11) identified a similar population of cells in early fracture callus, which they labeled “regenerative MPCs (rMPCs).” These cells resemble myofibroblasts, with high expression of myofibroblast marker genes including *Acta2*, *Lrrc15*, and *Tpm2* (9, 11). We observed similar cells in cluster 0 at day 5 with high expression of these same genes (Figure 2C and Supplemental Figure 2C), as well as other genes associated with cell motility, ECM, and wound healing (*Tnc*, *Tagln2*, *Col5a3*, *Timp1*) (52–54) (Supplemental Figure 2D). Notably, the proportion of these fibroblastic cells was greater in experimental mice with impaired callus formation than in control mice (Figure 2E). Perrin et al. (55) described “injury-induced fibrogenic cells” in the callus of normal healers (days 3–7), which represented an intermediate stage between skeletal progenitors and osteochondral cells. Finally, Xiao et al. (56) also identified populations of fibroblastic cells that constituted a large proportion of mesenchymal cells in the early (days 4 and 7) fracture callus, and reported that these cells were more abundant at day 7 in mice with systemic inflammation than in control mice. These authors suggested that starting from a common progenitor, fibroblastic cells (enriched in poor healers) and osteochondral cells (enriched in normal healers) represent alternative fates. Collectively, these studies highlight an important role for fibroblastic cells in the early fracture callus and indicate that failure to progress beyond a fibroblast-rich callus is a signature of poor healing.

An innovative feature of our study is that cells were targeted not only based on promoter activity of osteoblast genes but also based on physiological status (i.e., proliferating). This differs from other studies of fracture healing that used diphtheria toxin–based approaches to ablate cells based on promoter activity, thus targeting all Cre^+^ cells (57, 58). We observed that the HSV-TK/GCV system effectively and specifically ablated proliferating cells of the targeted osteoblast lineage. Moreover, our prior (22) and current studies did not reveal any off-target effects at the cellular or morphological levels. We acknowledge that ablating proliferating cells using the HSV-TK/GCV system may have limitations. In tumors, this system can target TK^–^ cells via gap junction–mediated transfer of monophosphorylated GCV from neighboring TK^+^ cells (33). However, we found no evidence that this “bystander effect” played a role in our results, as TK^+^ mice dosed with the gap junction inhibitor carbenoxolone had no rescue of their diminished callus formation (Supplemental Figure 11). We also showed that Cre^+^ROSA-TK^+^ mice do not have an inherent defect in fracture healing, as Adq-CreERT2 ROSA-TK mice had normal callus composition and only a modest reduction in callus bone volume (Supplemental Figure 12).

We used mouse genetic models that have been widely used to target osteoblast-lineage cells. However, a limitation of these models is that they may not be as biologically precise as intended. For example, *Ocn* may be expressed by hypertrophic chondrocytes during development and fracture healing (59, 60) that become osteoblasts (61); targeting these cells may have contributed to poor healing in Ocn-Cre ROSA-TK mice. In addition, while Dmp1-CreERT2 is an efficient tool for targeting osteocytes and mature osteoblasts (62), it may also be active in a population of perivascular cells within the cortical bone niche that can contribute a small number of cells to fracture callus, as well as a population of CD45^+^ bone marrow cells (63). These populations are likely to have been targeted in the Dmp1-CreERT2 ROSA-TK mice in our study and may have contributed to impairment of callus formation.

Mizoguchi and Ono (7) have highlighted the diverse origins of osteoblasts in development and regeneration, noting that osteoblasts can arise from multiple skeletal stem and progenitor cell populations (e.g., *Cxcl12^+^*, *Gli1^+^*, *Acta2^+^*, *Ctsk^+^*) located in different tissues (marrow, growth plate, periosteum) (6, 64–66). This view has been informed by a rich literature of lineage-tracing studies, and supports a model whereby expansion of local skeletal stem and progenitor cells, followed by their differentiation, is the predominant pipeline of bone-forming osteoblasts. However, the possibility that mature skeletal cells can be activated to contribute to bone regeneration has also been postulated (41). Our findings lend support to the latter, with evidence that during fracture healing, proliferation of callus cells at early and mature stages of osteoblast differentiation is an essential source for bone-forming osteoblasts.

In conclusion, our findings indicate that proliferation of callus cells at early and mature stages of osteoblast differentiation is essential for the formation of mineralized callus necessary for fracture healing. Blockade of osteoblastic cell proliferation leads to depletion of callus osteoblasts and chondrocytes, and persistence of immune cells and fibroblasts. These results suggest that designing interventions to target this process may have potential for treating fracture nonunion.

## Methods

### Sex as a biological variable.

Fractures were performed in female and male mice, data from which were combined. Preliminary statistical analysis showed no sex dependence.

### Chemicals and reagents.

See Supplemental Methods.

### Mouse care.

Mice were housed in standard conditions under 12-hour light/12-hour dark cycles, up to 5 per cage, with ad libitum food and water. Male and female mice were used in approximately equal number. Mice were euthanized via CO_2_ asphyxiation and secondary cervical dislocation.

### Col1-TK mice.

Transgenic 3.6Col1a1-TK (Col1-TK) mice were used to target proliferating (pre)osteoblasts, as previously described (21, 22). Mice were given ganciclovir (GCV) starting on the day of fracture (at 12 weeks of age) and were euthanized at 5, 10, or 21 days post-fracture.

### Fracture model.

A model of mid-diaphyseal femur fracture with intramedullary pin stabilization was used (22, 67). This model heals via secondary fracture healing, with intramembranous bone formation at the periphery and endochondral bone formation within the fracture gap.

### Drug treatments.

Osx-CreERT2, Dmp1-CreERT2, and Adq-CreERT2 mice were dosed with tamoxifen (TMX; 100 mg/kg in corn oil, oral gavage, twice per week) from 2 weeks before fracture until 2 weeks after fracture. Except where noted, mice were injected with GCV (8 mg/kg in water, once per day, i.p.) from the day of fracture to the indicated time (3, 5, 7, 10, or 14 days post-fracture). Some control mice were injected with either corn oil (control for TMX) or water (control for GCV). CBX (50 mg/kg, in saline, once per day, i.p.) was administered from the day of fracture until euthanasia 2 weeks after fracture.

### Single-cell RNA sequencing.

For scRNA-seq, 12 Col1-TK mice were fractured and treated with water (Control) or GCV (Experimental) for 5 or 10 days (*n* = 3 per time per treatment). Callus tissue was dissected and enzymatically digested, and single-cell suspensions (1 per mouse) were processed for library preparation (10x Genomics). An average of 8,700 cells per sample were sequenced (Illumina), resulting in detection of 23,140 genes per sample (average). Using Seurat (68), all cells from each time point were subjected to unbiased clustering. Based on bioinformatic analysis of the top 50 DEGs per cluster as well as gene expression plots, each cluster was annotated. Clusters primarily composed of mesenchymal cells were reclustered for higher resolution. Cell cycle stage was determined based on canonical gene expression profiles as previously described (28); cells assigned to either the S or the G_2_/M phase were judged to be actively proliferating.

### Generation, breeding, and validation of ROSA-TK mouse line.

We generated a novel Cre-inducible mouse (ROSA26-LSL-HSV-TK, or “ROSA-TK”) for targeted ablation of proliferating cells. A plasmid comprising the truncated herpes simplex virus thymidine kinase allele (HSV-ΔTK) (69) downstream of a CAG promoter and floxed stop cassette (Supplemental Figure 4) was knocked into the ROSA26 locus using TALENs. Genomic PCR confirmed knockin in 6 founder mice and F_1_ transmission (Supplemental Figure 5, A–C). Two heterozygous founders were used to establish the ROSA-TK lines backcrossed on a C57BL/6 background. ROSA-TK mice were crossed with Osx-CreERT2 (25), Dmp1-CreERT2 (70), Ocn-Cre (The Jackson Laboratory), and Adq-CreERT2 (The Jackson Laboratory) mice to generate control (Cre^–^TK^–^, Cre^–^TK^+^, Cre^+^TK^–^) and experimental (Cre^+^TK^+^) mice.

### Radiographic evaluation.

Fracture healing was assessed from a lateral radiograph (Faxitron) using the modified radiographic union score for tibia fractures (RUST) method adapted for mouse femora (71). Scores ranged from 2 (no callus on either cortex) to 6 (fully bridged callus on both cortices, but fracture line still visible).

### Micro-CT.

Fractured and intact contralateral femora were scanned by micro–computed tomography (micro-CT; Scanco; 10.5 μm voxel) for analysis of a 600-slice region of interest (ROI) centered at the fracture site. A single threshold (200 per mille) was used to determine standard bone measures (bone volume [BV], tissue volume [TV], BV/TV, volumetric bone mineral density [vBMD], and tissue mineral density [TMD]), inclusive of callus and cortical bone. To exclude the cortical bone within the ROI, a 2-threshold method was used (lower = 150, upper = 460 per mille), from which callus bone volume was determined.

### Histological analysis of callus.

Femora were decalcified and embedded in paraffin. Sagittal sections, 5 μm thick, were cut and stained by H&E and Picrosirius red/Alcian blue (PSRAB). Slides were scanned, and the periosteal callus was analyzed to determine percentage area of cartilage, woven bone, fibrous tissue, and periosteum (Supplemental Figure 1C).

### TK immunohistochemistry.

Anti-TK antibody (1:1,000; gift from William Summers, Yale University, New Haven, Connecticut, USA) was used to visualize HSV-TK expression in the callus region of paraffin-embedded histological sections, as previously described (72, 73). Anti-PCNA (1:500) was used to stain proliferating cells. DAB chromogen–positive cells were counted using image processing and particle counting tools in Fiji (https://fiji.sc). In brief, we counted the numbers of TK^+^ and PCNA^+^ cells (separately) in 4 ROIs of the fracture callus (top right, top left, bottom right, and bottom left), which are sites of woven bone formation.

### Co-immunofluorescence.

To visualize proliferating osteoblasts, histological sections were costained with either anti-PCNA (1:500) and anti-Osx (1:150), or anti-PCNA and anti-Ocn (1:500), and visualized by fluorescence microscopy. Proliferating cell nuclear antigen (PCNA) is expressed during the S phase of the cell cycle, and its staining in fracture callus closely mimics BrdU incorporation (29, 30). QuPath (74) was used to identify cells that were PCNA^+^ and either Osx^+^ or Ocn^+^. Percentage of proliferating early osteoblasts was determined as the number of PCNA^+^Osx^+^ cells divided by the total number of Osx^+^ nuclei, while percentage of proliferating mature osteoblasts was the number of PCNA^+^Ocn^+^ cells divided by the number of Ocn^+^ cells.

### Statistics.

Group sample sizes were determined a priori based on prior results and expected effect sizes. Personnel were blinded to animal genotype and treatment during data collection and analysis. Analyses were carried out in GraphPad Prism 9.0. Statistical tests used are stated in the figure legends. *P* < 0.05 was defined as significance.

### Study approval.

This study was approved by the Washington University IACUC (22-0384 and 21-0050) at Washington University in St. Louis, St. Louis, Missouri, USA.

### Data availability.

scRNA-seq data are available at the NCBI’s Gene Expression Omnibus database (GEO GSE343272). ROSA-TK mice will be made available upon reasonable request. Supporting data values for all data points shown in graphs and values behind any reported means are provided in the Supporting Data Values Excel file.

## Author contributions

Study design: NRG, AFC, JAM, DMO, and MJS. Data collection and analysis: all authors. Manuscript preparation: NRG, AFC, MO, and MJS. Manuscript review: all authors.

## Conflict of interest

The authors have declared that no conflict of interest exists.

## Funding support

This work is the result of NIH funding, in whole or in part, and is subject to the NIH Public Access Policy. Through acceptance of this federal funding, the NIH has been given a right to make the work publicly available in PubMed Central.

- US Department of Defense grant PW81XWH-21-1-0386 (to MJS).
- NIH grants R21 AR076636, P30 AR074992, and S10 RR023660 (to MJS).
- NIH grant R01 AR079246 (to DMO).
- NIH grant T32 AR060719 (to NRG, AFC, and KRH).
- NIH grant S10 RR027552 to Alafi Neuroimaging Core.

## Supplementary Material

Supplemental data

Supplemental table 1

Supporting data values

## Figures and Tables

**Figure 1:**
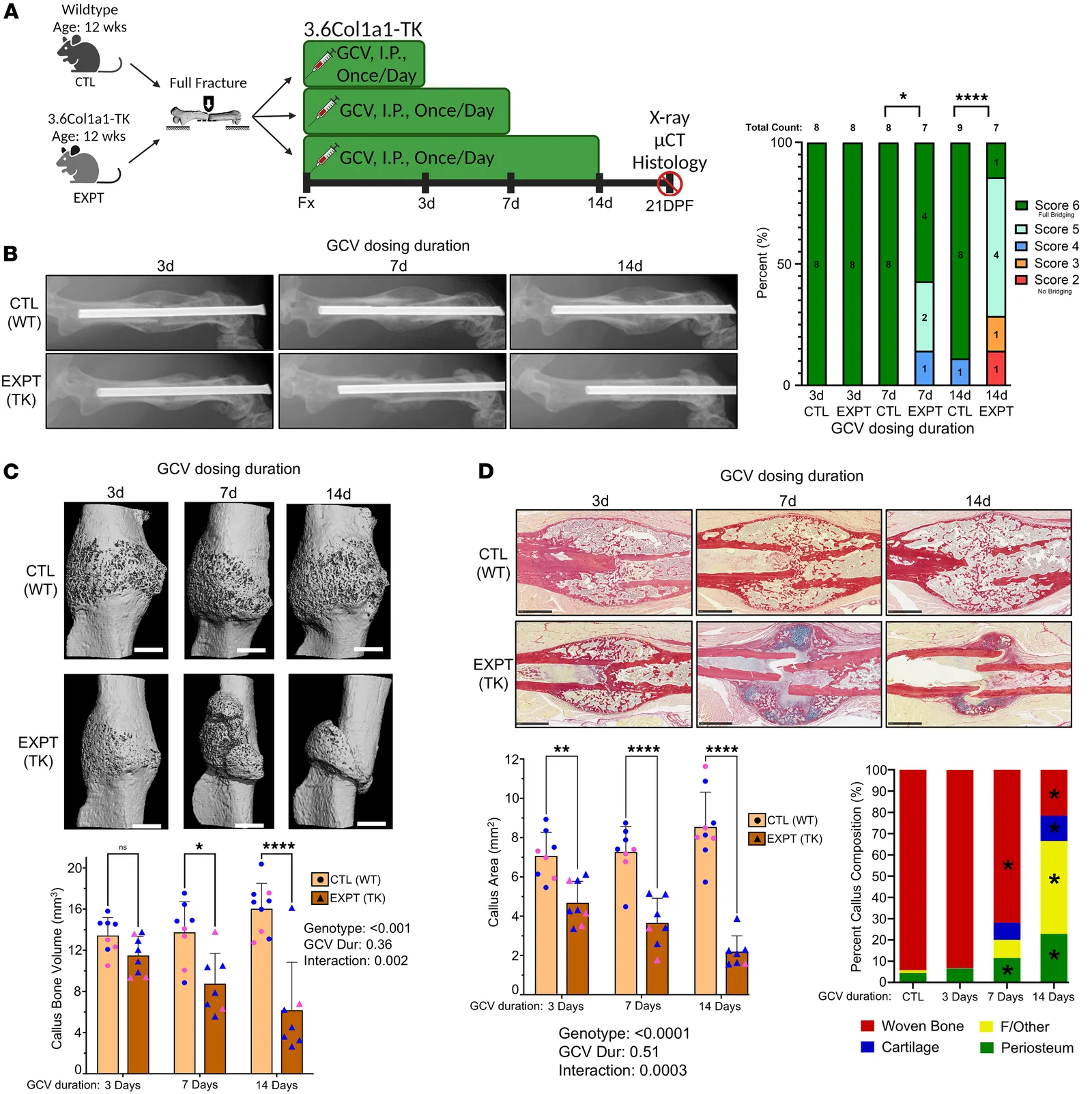
Ablation of proliferating 3.6Col1a1-lineage cells in the first 3–14 days following fracture causes defects in callus formation at 21 days post-fracture. (**A**) Femur fractures were created in 12-week-old wild-type control (CTL) and Col1-TK experimental (EXPT) mice, followed by ganciclovir (GCV) dosing for 3, 7, or 14 days (*n* = 7–9). (**B**) Left: Representative radiographs at 21 days post-fracture (DPF). Right: Quantification of radiographic healing. (Score 2 = neither cortex bridged; 6 = both cortices bridged.) (**C**) 3D micro-CT images at 21 DPF (scale bars: 1 mm). (**D**) Sagittal callus sections were stained with Picrosirius red to visualize collagen-rich bone and Alcian blue for proteoglycan-rich cartilage, and normalized callus composition was determined (scale bars: 1 mm). Graphs depict mean ± SD; individual data points are shown (pink, female; blue, male). Statistical differences were determined by χ^2^ test (**B**) or 2-way ANOVA with Tukey’s post hoc test (**C** and **D**); *P < 0.05, **P < 0.01, ****P < 0.0001; NS: *P* > 0.05.

**Figure 2:**
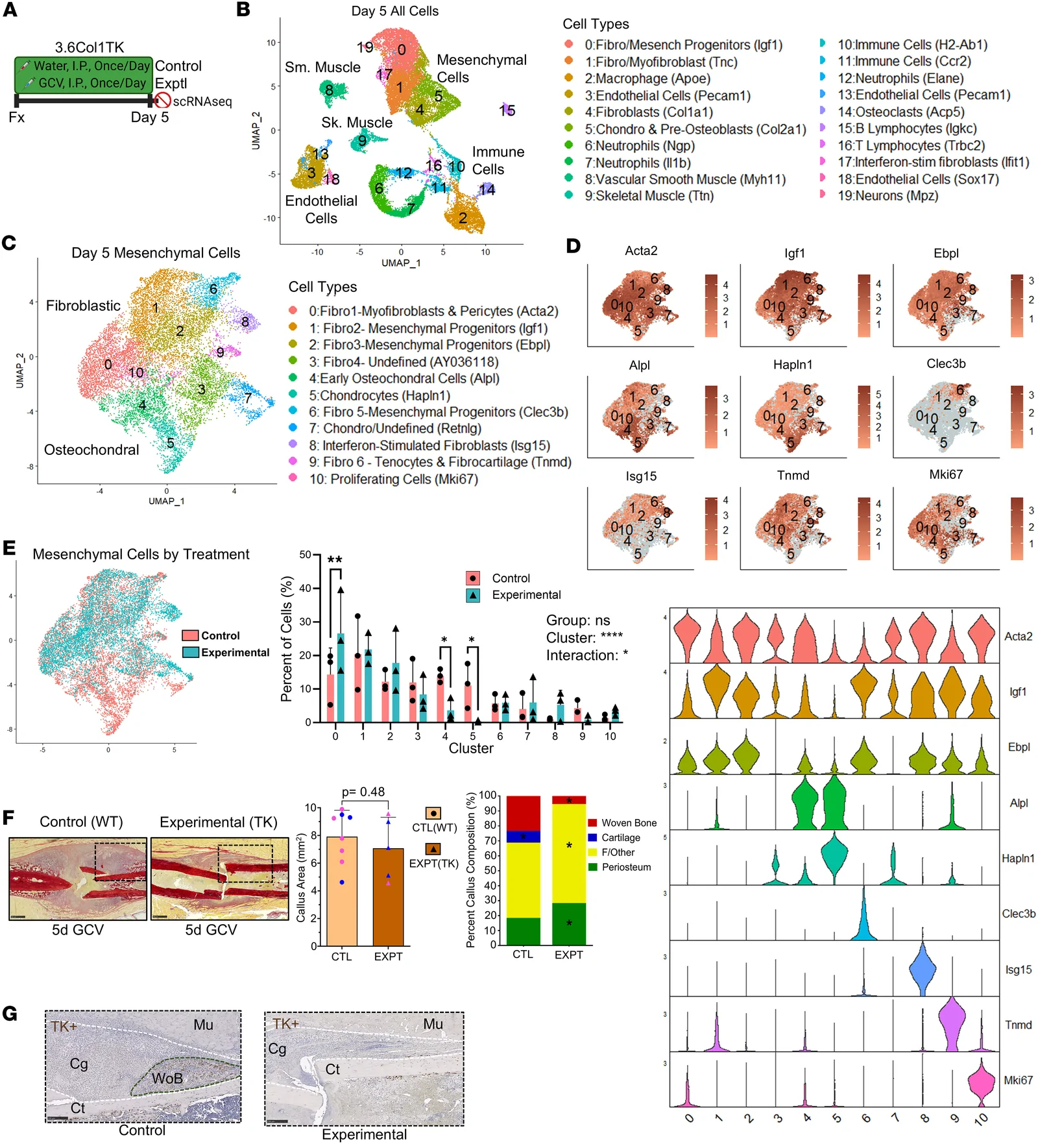
Ablation of proliferating 3.6Col1a1-lineage cells for 5 days after fracture depletes callus osteoblasts and chondrocytes and alters callus composition. (**A**) After femur fracture at 12 weeks of age, Col1-TK mice were treated with water (Control, *n* = 3) or GCV (Exptl, *n* = 3) for 5 days. Cells isolated from whole-fracture callus were subjected to scRNA-seq. (**B**) UMAP plot of total callus cells with annotated cell cluster identities based on canonical gene expression and bioinformatic analysis. (**C**) Mesenchymal cells were selected in silico and reclustered. (**D**) Expression of representative differentially expressed genes for each of 9 clusters projected on UMAPs and displayed as violin plots. (**E**) Mesenchymal cells were identified by treatment group, and the percentage of cells in each cluster was quantified (*n* = 3). (**F**) Histological sections of callus at 5 DPF from Control (*n* = 8; WT + GCV, or Col1-TK + water) and Exptl (*n* = 5; Col1-TK + GCV) were stained with Picrosirius red and Alcian blue (PSRAB) and used to determine total callus area, and percentage area of woven bone (red), cartilage (blue), fibrous (F)/other tissue, and periosteum. (**G**) High-power fields (from **F**) of TK IHC of Control (Col1-TK + water) and Experimental (Col1-TK + GCV) mice (white dashed line, callus periphery; green dashed line, woven bone area; Mu, muscle; Cg, cartilage; Ct, cortical bone; WoB, woven bone). Statistical differences were determined by 2-way ANOVA with Holm-Šidák post hoc test (**E**) or 2-tailed *t* test (**F**); *P < 0.05, **P < 0.01, ****P < 0.0001. Scale bars: PSRAB, 0.5 mm; TK, 1 mm.

**Figure 3:**
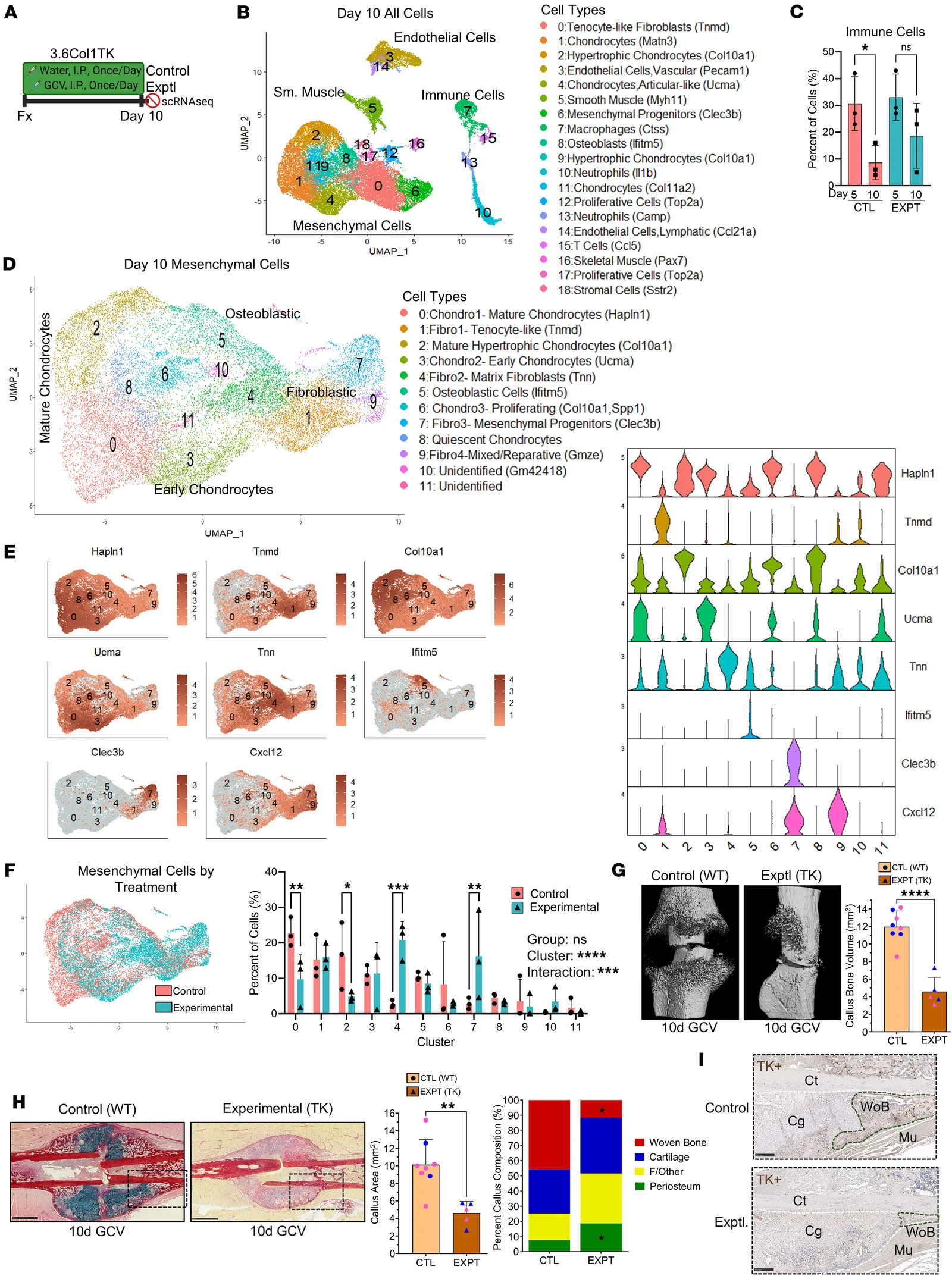
Ablation of proliferating 3.6Col1a1-lineage cells for 10 days after fracture depletes callus osteoblasts and chondrocytes and significantly reduces callus bone volume. (**A**) After femur fracture at 12 weeks of age, Col1-TK mice were treated with water (Control, *n* = 3) or GCV (Exptl, *n* = 3) for 10 days. Cells isolated from whole-fracture callus were subjected to scRNA-seq. (**B**) UMAP plot of total callus cells with annotated cluster identities. (**C**) The number of cells from immune clusters on day 5 (clusters 2, 6, 7, 10, 11, 12, 14, and 16) and day 10 (clusters 7, 10, 13, and 15) normalized to total cell numbers. (**D**) Mesenchymal cells were selected in silico and reclustered. (**E**) Expression of select differentially expressed genes displayed as violin plots and projected on UMAPs. (**F**) Mesenchymal cells were identified by treatment group, and the percentage of cells in each cluster was quantified (*n* = 3). (**G**) Callus bone volume was quantified by micro-CT (*n* = 5–8). (**H**) Histological sections of callus at 10 DPF were analyzed to determine total callus area and callus tissue composition. (**I**) Representative images of TK IHC of Control (Col1-TK + water) and Experimental (Col1-TK + GCV). Statistical differences were determined by 2-way ANOVA with Holm-Šidák post hoc test (**C** and **F**) or 2-tailed *t* test (**G** and **H**). *P < 0.05, **P < 0.01, ***P < 0.001, ****P < 0.0001. Scale bars: PSRAB, 1 mm; TK, 1 mm.

**Figure 4:**
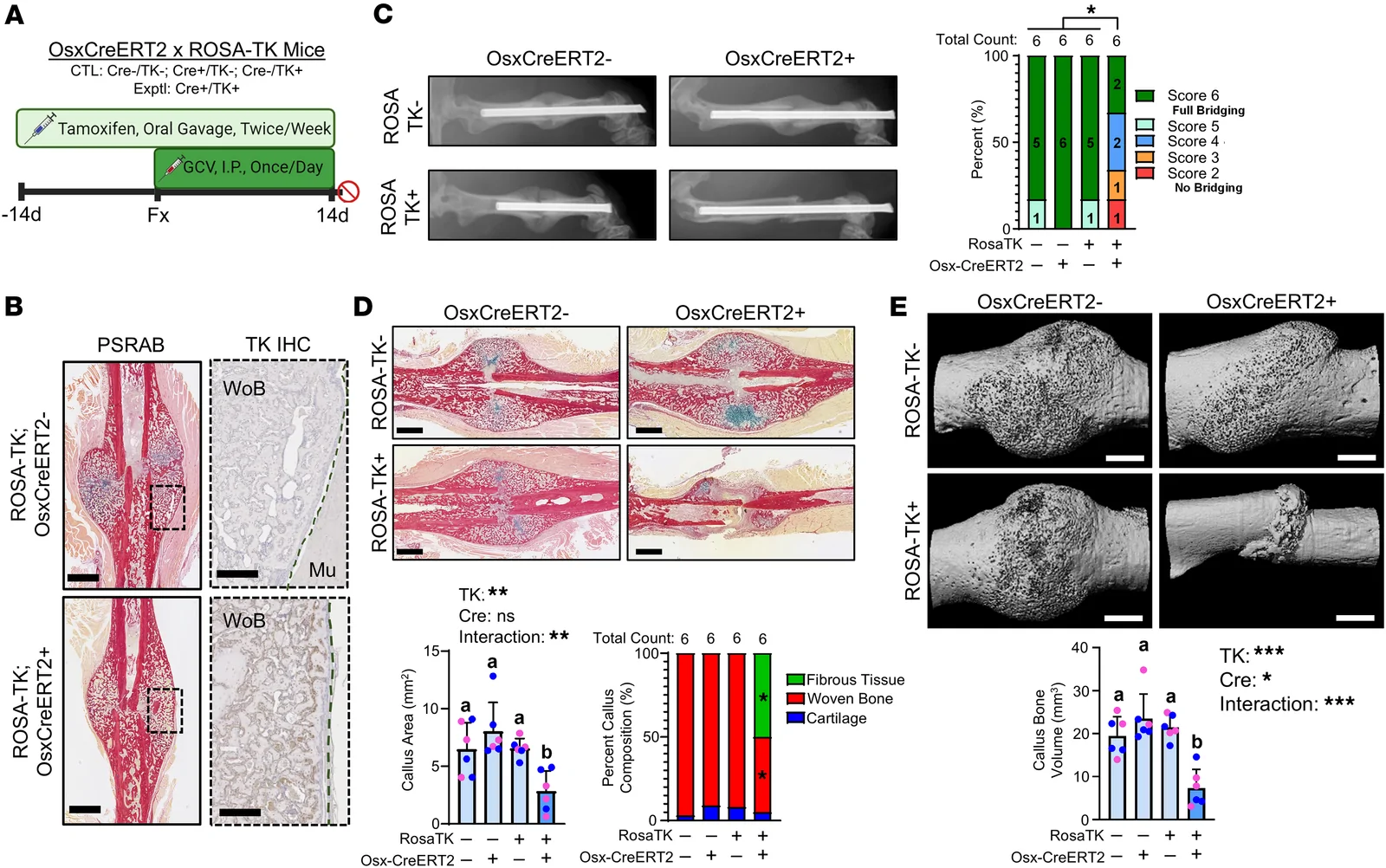
Ablation of proliferating Osterix-expressing cells during fracture healing impairs callus bridging and mineralization. (**A**) Mice were treated with TMX starting at 10 weeks of age, followed by femur fracture at 12 weeks, followed by 2 weeks GCV treatment. Healing was assessed at 14 DPF. Genotype controls were mice lacking either Osx-CreERT2 (Cre^–^) or ROSA-TK (TK^–^) alleles; experimental mice had both alleles (Cre^+^TK^+^) (*n* = 6). (**B**) TK^+^ mice were treated with GCV (Cre^–^) or water (Cre^+^) for TK IHC, which shows robust TK expression in callus woven bone only in Cre^+^ mice (green dashed line, callus periphery; WoB, woven bone; Mu, muscle). (**C**) Left: Representative radiographs. Right: Quantification of radiographic healing. (**D**) Histological analysis of PSRAB-stained sections to assess callus size and composition. (**E**) Callus bone volume was quantified by micro-CT. Statistical differences were determined by χ^2^ test (**C**) or by 2-way ANOVA with Holm-Šidák post hoc test (**D** and **E**). *P < 0.05, **P < 0.01, ***P < 0.001. Groups with different letters are significantly different (*P* < 0.05). Scale bars: TK, 0.25 mm; PSRAB, 1 mm; micro-CT, 1 mm.

**Figure 5:**
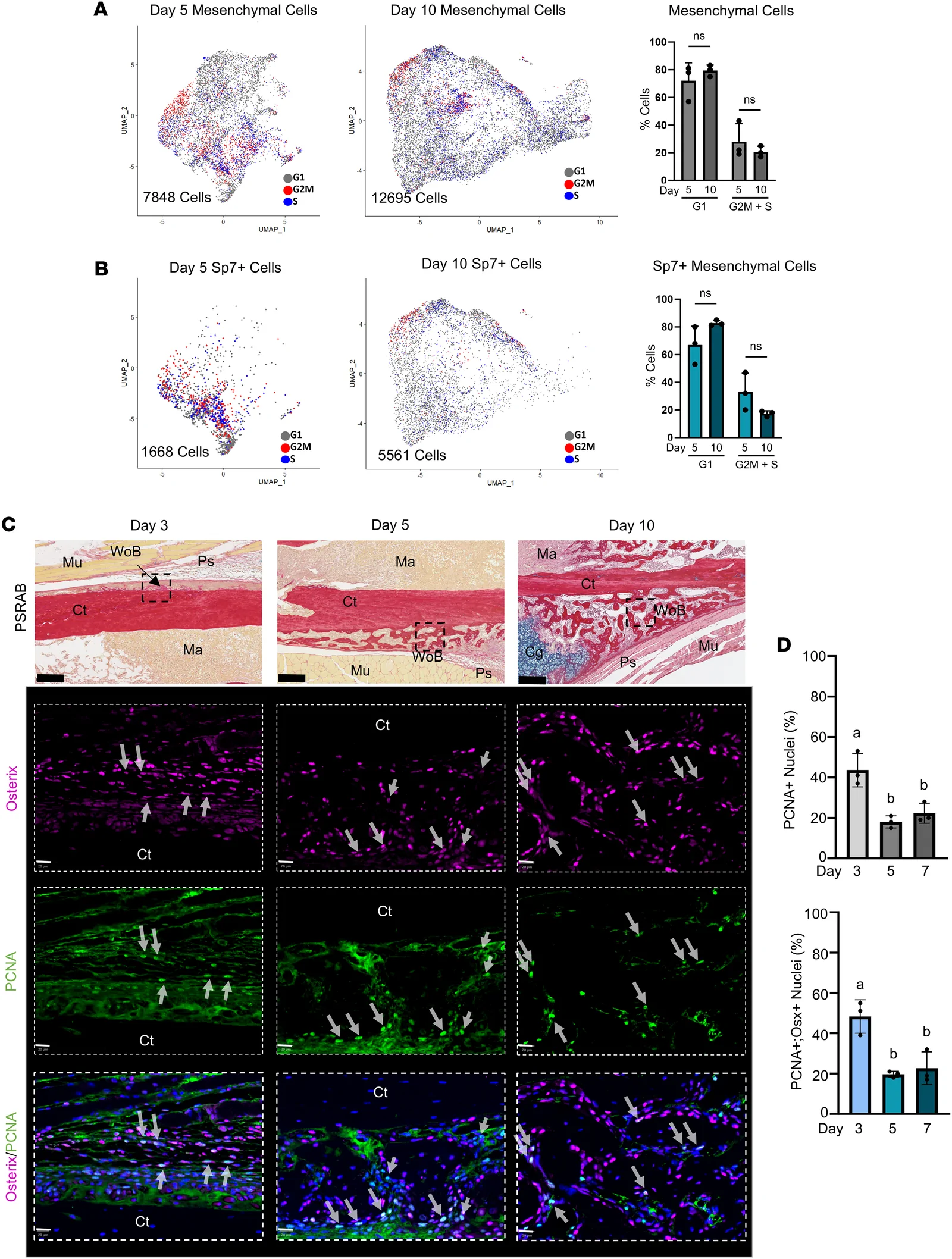
Sp7/Osterix-expressing callus cells proliferate during fracture healing. (**A**) Mesenchymal cells from scRNA-seq analysis at day 5 and day 10 after fracture annotated for cell cycle stage. (**B**) Mesenchymal cells with high *Sp7* gene expression annotated for cell cycle. (**C**) Low-power images of fracture callus sections from Control mice at 3, 5, and 10 DPF stained by PSRAB (dashed box depicts area of woven bone callus; Mu, muscle; Cg, cartilage; Ct, cortical bone; Ps, periosteal surface; WoB, woven bone; Ma, marrow), and high-power images of woven bone regions stained by immunofluorescence for Osterix and the proliferation marker PCNA. Scale bars: PSRAB, 200 mm; IF, 20 mm. Arrows, Osx^+^PCNA^+^ cells. (**D**) Quantification of percentage PCNA^+^ nuclei and Osx^+^PCNA^+^ nuclei determined in entire callus. Groups with different letters are significantly different (*P* < 0.05). *n* = 3; graphs depict mean ± SD. Statistical differences were determined by 2-way ANOVA (**A** and **B**) or 1-way ANOVA (**D**) with Holm-Šidák post hoc test.

**Figure 6:**
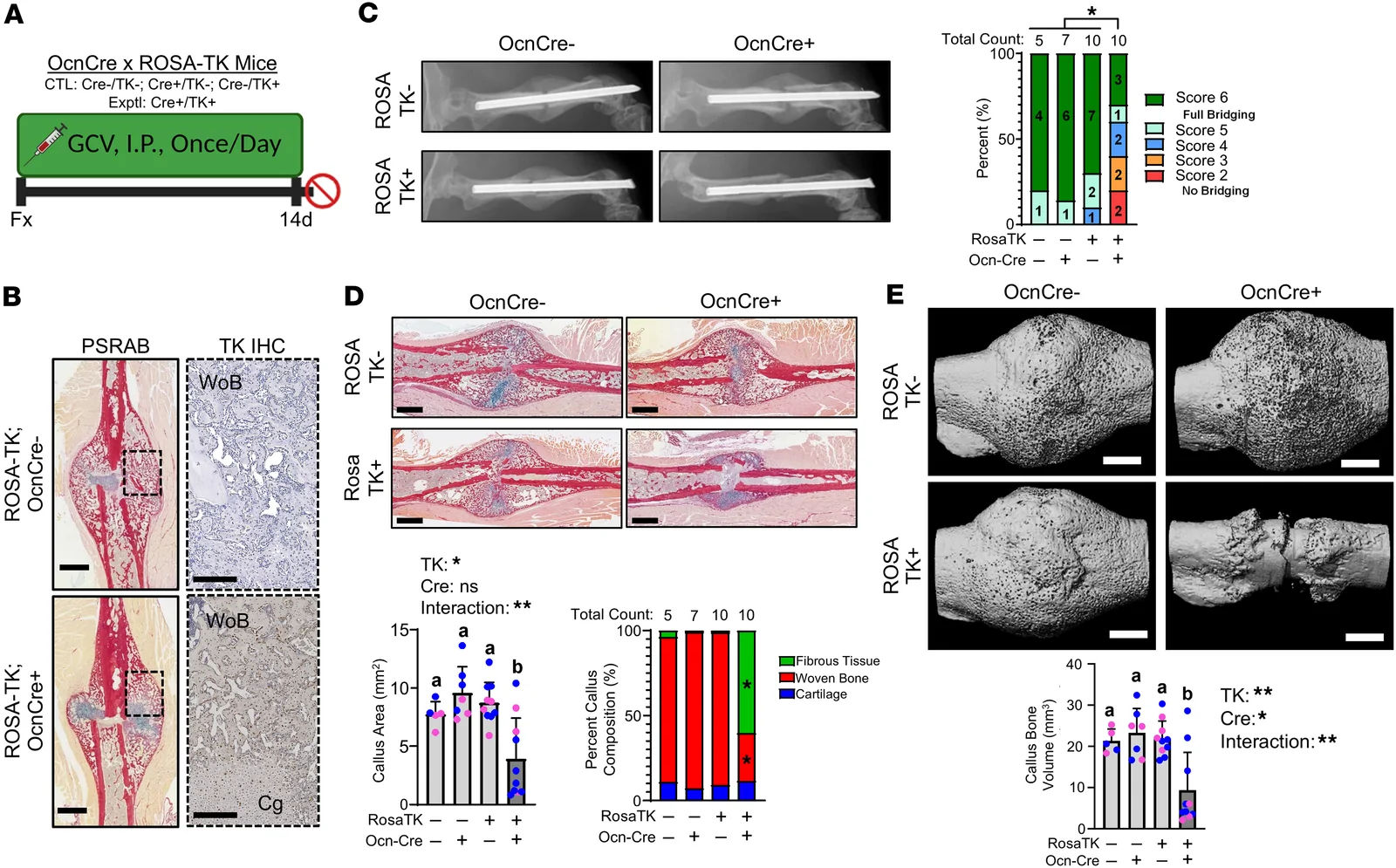
Ablation of proliferating osteocalcin-lineage cells during fracture healing impairs callus formation. (**A**) Femur fracture was performed at 12 weeks of age, followed by 2 weeks of GCV treatment. Healing was assessed 14 days after fracture. Genotype controls included mice lacking either Ocn-Cre or ROSA-TK alleles; experimental mice were Cre^+^TK^+^ (*n* = 5–10). (**B**) TK^+^ mice were treated with GCV (Cre^–^) or water (Cre^+^) for HSV-TK IHC, which shows TK expression in callus woven bone in Cre^+^ mice only. (**C**) Left: Representative radiographs. Right: Quantification of radiographic healing. (**D**) Histological analysis of fracture callus composition on PSRAB-stained slides showed that experimental mice have altered composition, with less woven bone and more fibrous tissue compared with control. (**E**) Callus bone volume quantified by micro-CT. Statistical differences were determined by χ^2^ test (**C**) or 2-way ANOVA with Holm-Šidák post hoc test (**D** and **E**). *P < 0.05, **P < 0.01. Groups with different letters are significantly different (*P* < 0.05). Scale bars: TK, 0.25 mm; PSRAB, 1 mm; micro-CT, 1 mm.

**Figure 7:**
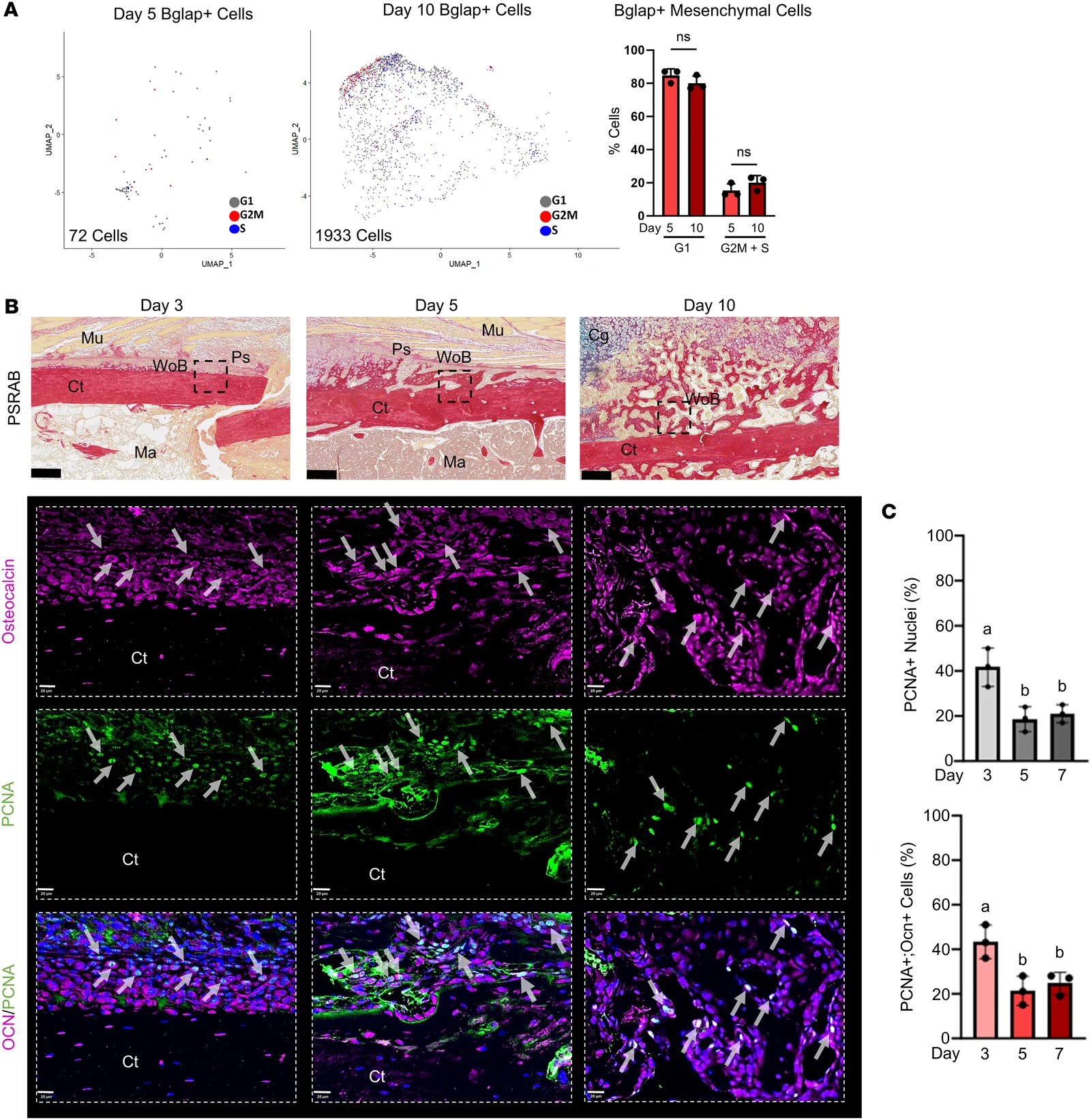
Bglap/osteocalcin-expressing callus cells proliferate during fracture healing. (**A**) From scRNA-seq analysis at 5 and 10 DPF, mesenchymal cells with high *Bglap* expression were annotated for cell cycle stage (*n* = 3). (**B**) Low-power images of fracture callus sections from control mice at 3, 5, and 10 DPF stained by PSRAB (dashed box depicts area of woven bone callus; Mu, muscle; Cg, cartilage; Ct, cortical bone; Ps, periosteal surface; WoB, woven bone; Ma, marrow), and high-power images of woven bone areas stained by immunofluorescence for osteocalcin (Ocn) and the proliferation marker PCNA. Scale bars: PSRAB, 200 mm; IF, 20 mm. Arrows, Ocn^+^PCNA^+^ cells. (**C**) Quantification of percentage PCNA^+^ nuclei and Ocn^+^PCNA^+^ nuclei determined in entire callus. *n* = 3; graphs depict mean ± SD. Groups with different letters are significantly different (*P* < 0.05). Statistical differences were determined by 2-way ANOVA (**A**) or 1-way ANOVA (**C**) with Holm-Šidák post hoc test.

**Figure 8:**
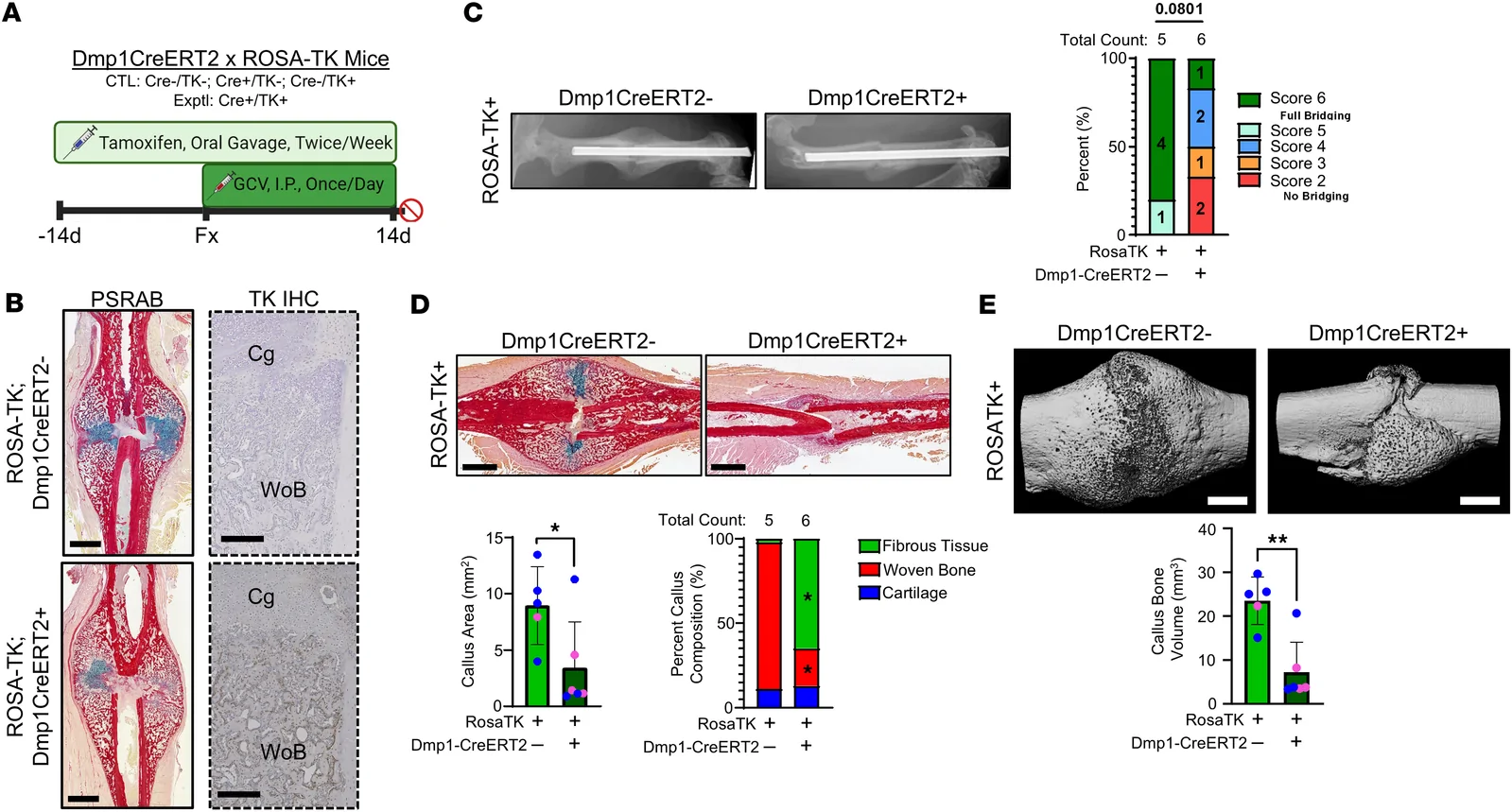
Ablation of proliferating Dmp1-expressing cells during fracture repair impairs callus bridging and mineralization. (**A**) Mice were treated with TMX starting at 10 weeks of age, followed by femur fracture at 12 weeks, followed by 2 weeks of GCV treatment. Healing was assessed 14 days after fracture. Control mice were Cre^–^TK^+^, while experimental mice were Cre^+^TK^+^. (**B**) TK^+^ mice were treated with water for HSV-TK IHC, which shows robust TK expression in callus woven bone only in Cre^+^ mice. (**C**) Fracture callus bridging was quantified based on radiographic images. (**D**) Histological analysis of PSRAB-stained sections was used to assess callus size and composition. (**E**) Callus bone volume was quantified using micro-CT. Statistical differences were determined by χ^2^ test (**C**) or by 2-tailed *t* test (**D** and **E**). *P < 0.05, **P < 0.01. Scale bars: PSRAB, 1 mm; TK IHC, 0.25 mm; micro-CT, 1 mm.

## Version 1 09/08/2026

In-Press Preview

## Version 2 10/08/2026

Electronic publication
