# Supplementary material for: Fracture healing requires proliferation of callus cells at early and mature stages of osteoblast differentiation
Source: JCI Insight. 2026 Sep 8;11(19):e196751. doi: 10.1172/jci.insight.196751 (PMC13726425; doi:10.1172/jci.insight.196751)
Supplement: Supplementary file 1 [file jciinsight-11-196751-s144.pdf]

Gould et al, "Fracture Healing Requires Proliferation of Callus Cells at Early and Mature Stages of Osteoblast Differentiation"

JCI Insight

Supplemental Materials

Supplemental Results and Methods (incl references cited)

Supplemental Figures S1-S14

Supplemental Table S1

Supplemental Results

Single-cell RNAseq of fracture callus at days 5 and 10 post-fracture reveals dynamic mesenchymal cell populations that are altered by ablation of proliferating 3.6Col1a1 lineage cells

We profiled the early callus of Col1-TK mice using single-cell RNA sequencing (scRNAseq) to 1) examine differences between Control and Experimental groups, and 2) characterize the mesenchymal cell populations in the early callus. Following fracture, Col1-TK mice were treated either with water (Control) or GCV (Exptl) for 5 or 10 days (Figs 2A, 3A). Periosteal whole-callus tissue was dissected and enzymatically digested, and the resulting cell samples were profiled using scRNAseq. For analysis, Control and Experimental samples were integrated before clustering to facilitate comparisons between groups, which was our primary objective. Data from a total of 60,705 cells from day 5 and 43,585 cells from day 10 were available for analysis. Unbiased cluster analysis was performed first for all callus cells, and then for the subset of mesenchymal cells. We assigned cell identities to each cluster based on canonical gene expression and bioinformatic analysis of differentially expressed genes. Here we present these results in detail, with reference to recent, relevant publications.

Day 5. Unbiased cluster analysis of all cells identified 20 clusters that contained cell types from five major lineages. The majority (88%) of cells were mesenchymal (*Prrx1*+/*Col1a1*^{Hi}, *Myh11*-; Clusters 0, 1, 4, 5, 17), immune (*Ptprc*/CD45+; Clusters 2, 6, 7, 11, 12, 14, 15, 16) or endothelial (*Pecam1*+; Clusters 3, 13, 18). The remainder were vascular smooth muscle (*Myh11*+; Cluster 8), skeletal muscle (*Myod1*+; Cluster 9) and a small number (0.3%) of neurons (*Mpz*+; Cluster 19) (Fig 2B, Suppl Fig S2A). The skeletal muscle cluster was negative for *Pdgfra* and *Ly6a*, indicating that it did not contain FAP cells (1) and is likely contaminant tissue. Annotations were based on analysis of top DEGs along with examination of gene expression maps (see Table S1 for top 10 DEGs). The presence of these diverse cell types in the fracture callus is consistent with recent reports. Novak et al. (2) identified multiple mesenchymal cell clusters on day 3 after fracture, in addition to endothelial, smooth muscle and skeletal muscle satellite clusters (all CD45-). They reported that most of their callus cells (88%) were CD45+, which is much greater than the 33% of CD45+ cells we observed on day 5. We attribute this difference to the dynamic influx of immune cells in the first three days after fracture, followed by their reduction and the expansion of tissue cells. Wang et al. (3) also reported diverse populations of immune cells in the fracture callus at days 5 and 15. These included neutrophils marked by high expression of

Ngp and *S100a8*, which we observed in our Clusters 6, 7 and 12, macrophages (*Apoe*+, *C1qa/b/c*+, *Csf1r*+, *Arg1*+; Cluster 2) and osteoclasts (*Ctsk*+, *Acp5*+; Cluster 14).

The *Prrx1*+/*Col1a1*^{Hi} mesenchymal clusters had a distinct signature of extracellular matrix (ECM) production, with high expression of other collagens (*Col1a2, Col3a1*) and fibronectin (*Fn1*). (For conciseness, we use "mesenchymal" throughout the paper to refer to mesenchymal cells excluding skeletal muscle). Mesenchymal cells were transcriptionally distinct from muscle and non-mesenchymal callus cells. The only non-mesenchymal cluster that was close to the mesenchymal clusters (in UMAP space) was the small group of neuronal cells (*Mpz*+; Cluster 19). Notably, in the callus of Experimental mice, chondrocytes and pre-osteoblasts (Cluster 5; top DEGs include *Col2a1*, *Col9a3*, *Ibsp, Pth1r*) were significantly depleted compared to control (Suppl Figs S2B). These cells comprised 12% of all callus cells in Control mice, but only 1% in Experimental mice. No other clusters were significantly depleted (or enriched) between treatment groups based on this analysis of all callus cells, which indicates specific ablation of proliferating 3.6Col1a1-lineage osteochondral cells.

To enhance resolution of mesenchymal sub-populations, cells of the original mesenchymal clusters (0, 1, 4, 5, 17) were selected *in silico* for re-clustering. Analysis of this mesenchymal sub-population identified 11 clusters (Fig 2C, Suppl Fig S2C, Table S1). Appropriate to their mesenchymal classification, nearly all cells in these clusters had high expression of multiple collagen and other ECM formation and remodeling transcripts, including *Col1a1*, *Col3a1* and *Fn1* (>99% of cells), and *Mmp2* and *Timp1* (>95% of cells) (Suppl. Fig. S2D), indicating a strong signature for ECM synthesis and remodeling in the early callus (2, 4). Clusters at this timepoint were mostly comprised of a diverse population of fibroblastic cells (Clusters 0, 1, 2, 3, 6, 8, 9; accounting for 77% of cells) as well as chondrocytes and osteoblastic cells (Clusters 4, 5; 16%), and small populations of myeloid cells (Cluster 7; 4%) and indistinct proliferating cells (Cluster 10; 2%).

Of the seven fibroblast clusters, three contiguous ones (6, 1, 2) were labeled as Mesenchymal Progenitors, with Cluster 6 being the least differentiated. Cells in Cluster 6 are similar to the mesenchymal progenitor cells (MPCs) reported by others (3, 5), based on strong and highly specific expression of *Clec3b*, as well as *Cd34*, *Ly6a* and *Pi16* (Fig 2D, Suppl Fig S2C). *Eln* and *Cxcl14* are also DEGs specific to this cluster. Cluster 1 is marked by DEGs *Igf1*, *Abi3bp*, *Sfrp1* and *Ogn*. *Sfrp1* is a negative regulator of Wnt signaling that was expressed in other Day 5 clusters and is not thought to be cell-type specific. However, Wang et al. (3) reported *Sfrp1* expression in a population of "regenerative" MPCs, and it has been described as a regulator of the fibroblast-myoblast transition in pulmonary

fibroblasts following injury (6). Cluster 2 cells are also fibroblastic/progenitor-like, marked by DEGs *Ebpl*, *Scn7a*, *Kif26b* and *Cxcl12*. Of note, we have reported that *Kif26b* plays a role in the differentiation of progenitor cells during the process of osteophyte formation following joint injury (7), suggesting that it may play a similar role in the transition from multipotent progenitors (Cluster 6) to osteochondral cells (Cluster 4, 5). *Cxcl12* is strongly expressed in Clusters 1, 2 and 6, suggesting a population of Osteo-CAR-like cells in periosteal fracture callus (Suppl Fig S2E). Although *Cxcl12* is expressed by periosteal cells (8), it is more classically associated with bone marrow perivascular cells that can contribute to osteoblasts formation during skeletal regeneration (9).

Other fibroblast clusters were more differentiated. Cluster 0 is a large (21% of mesenchymal cells) subpopulation with a myofibroblastic signature, based on DEGs including canonical myofibroblast genes *Acta2*, *Actg2*, *Tmp2* and *Tagln2* (Fig 2D, Suppl Fig S2C) (10, 11). The presence of myofibroblasts in the early fracture callus is consistent with an earlier report (12). Moreover, Cluster 0 has a profile similar to the (myofibroblast-like) proliferative periosteal progenitor cells (PPCs) described in the early callus by Yao et al. (5); these cells were recently re-labeled as "regenerative MPCs" (3). Other DEGs of note in Cluster 0 are *Col5a3* and *Lrrc15*. Collagen type V transcripts are strongly upregulated by cardiac fibroblasts following ischemic injury and Col V regulates the size of the fibrotic scar (13). In fracture callus, *Lrrc15* is expressed by regenerative MPCs (3). Cluster 9 is a small population (2.6%) of tenocyte-like fibroblasts (*Tnmd*+). Nookaew et al. have reported a *Tnmd*-expressing cluster of cells harvested from the periosteum of intact bone (14). Moreover, tendon stem/progenitor cells (TSPCs) express *Tnmd* (15), suggesting that cluster 9 is a population of progenitor cells with a tenocyte signature. Lastly, Cluster 8 cells are identified as interferon-stimulated fibroblasts; they express *Col1a1*, *Col3a1* and *Fn1*, but their top DEGs are all interferon related, including *Isg15*, *Bst2*, *Ifit1* and *Stat1* (Suppl Fig S2C). Cluster 3 expresses matrix genes associated with fibroblasts but has low gene features and was not defined.

The most differentiated mesenchymal cells were in Clusters 4 and 5. We annotated Cluster 4 as early osteochondral cells, based on DEGs *Alpl*, *Sp7*, *Pth1r and Inhba*. Notably, *Inhba* expression in the day 5 callus is reported to mark proliferative periosteal progenitors (PPCs) as well as chondrocytes (5). Consistent with this, we find that *Inhba* is most abundant in the PPC-like Cluster 0 as well as the adjacent osteochondral Cluster 4. A signature consistent with mature osteoblasts is also seen in a small subset of Cluster 4 cells, based on the DEGs *Ibsp* and the expression of *Bglap/Bglap2*. Cluster 5 was made up of early and mature chondrocytes, based on cartilage matrix DEGs *Hapln1*, *Col2a1*, *Snorc* and

Matn3. A subset of cells in this cluster also express the hypertrophic chondrocyte markers *Col10a1* and *Ihh*.

When viewed as a whole, many of the mesenchymal cells fall on a differentiation trajectory starting from the *Clec3b+* progenitor-like cells of Cluster 6, through several stages of fibroblasts (Clusters 1, 2 and 0) to chondrocytes and osteoblastic cells (Clusters 4, 5). (Clusters 8, 9, 3 and 7 are on different paths. Clusters 8 and 9 are minor fibroblastic populations, while Clusters 3 and 7 are undefined fibroblasts and chondrocytes.

Regarding the effects of experimental treatment (GCV), within the 11 mesenchymal clusters only the osteochondral cells of Clusters 4 and 5 were significantly depleted in Experimental mice compared to Control (Fig 2E). In Control mice, cells from these two clusters comprised 25% of all mesenchymal cells, whereas in Experimental mice they comprised only 4%. On the other hand, the myofibroblastic cells of Cluster 0 were significantly enriched in the Experimental fracture callus (CTL: 14%, EXPT: 27%). We also observed that the majority (80%) of Cluster 8 cells (interferon-stimulated fibroblasts) were from experimental mice. Importantly, the abundance of mesenchymal progenitor cells (clusters 6, 1, 2) was not affected by experimental treatment, indicating no depletion of MPCs. To confirm that GCV treatment of Col1-TK mice targets proliferating cells, we counted the number of cells in the cell cycle (S or G2/M phases) in Control and Experimental groups (Suppl Fig S2F). The greatest reductions in proliferating cells with GCV treatment was observed in Clusters 4 and 5, consistent with the significant depletion of cells in these clusters and confirming the targeted ablation of proliferating osteochondral cells. Taken together, these data indicate that committed osteochondral cells, but not their progenitors, are specifically depleted by GCV treatment of Col1-TK mice, and perhaps as a consequence there is a relative increase in the number of myofibroblastic/PPCs. We speculate that the latter represents compensation whereby progenitor cells continue to proliferate under persistent signals driving osteochondral cells to repair the fracture.

Day 10. Unbiased cluster analysis of all cells in the fracture callus on day 10 identified cell types of five major lineages. Like day 5, the majority (90%) of cells were in clusters identified as mesenchymal (*Prrx1*+/*Col1a1*^{Hi}, *Myh11*-; Clusters 0, 1, 2, 4, 6, 8, 9, 11), immune (*Ptprc*/CD45+; Clusters 7, 10, 13, 15) or endothelial (*Pecam1*+; Clusters 3, 14) Fig 3B, Suppl S3A). Also present were smooth muscle (*Myh11*+; Cluster 5) and skeletal muscle cells (*Pax7*+; Cluster 16); 3% of cells were indistinct proliferative cells (Clusters 12, 17, 18). From days 5 to 10, callus cell composition shifted with a large

increase in mesenchymal cells (from 46 to 69%) reflecting maturation of callus ECM. There was a corresponding decrease in immune cells (from 33 to 12%), reflecting attenuation of the initial immune response, with fewer neutrophils and macrophages.

Notably, callus osteochondral cells were significantly depleted by GCV treatment. Specifically, cells in closely related Clusters 1 (chondrocytes; top DEGs *Hapln1, Matn3, Col2a1, Col9a1*) and Cluster 2 (hypertrophic chondrocytes; top DEGs *Mmp13*, *Col10a1*, *Spp1*, *Ihh*) comprised 33% of all cells in Control mice, but only 9% in Exptl mice (Suppl Fig S3B). The opposite shift was seen in cells from Clusters 5 (smooth muscle cells; DEGs *Acta2*, *Tagln*/SM22a, *Myh11*, *Myl9*) and 6 (mesenchymal progenitors; DEGs *Clec3b*, *Mfap5*, *Igf1*, *Pi16*), which were relatively enriched in Experimental mice compared to Control.

To refine the analysis of mesenchymal sub-populations, cells of the original mesenchymal clusters (0, 1, 2, 4, 6, 8, 9, 11) were selected *in silico* for re-clustering. Analysis of this sub-population identified 11 contiguous clusters (and one outlier containing 0.6% of all cells, which was excluded from further analysis). We assigned cell identities as described above (Fig 3C-D, Suppl Fig S3C, Table S1). Like day 5, collagen and other ECM-related transcripts were broadly expressed on day 10, with >99% of cells expressing *Col1a1*, *Col3a1* and *Fn1,* and >90% expressing *Mmp2 and Timp1*, consistent with a dynamic ECM environment (Suppl Fig S3I). At the same time, more than 90% of mesenchymal cells expressed *Col2a1* and *Acan*, indicating a strong chondrogenic signature (Fig. S3J). In fact, the most notable change in the mesenchymal populations of days 5 versus 10 was the increased proportion of cells in chondrocytes clusters (0, 2, 3, 6, 8); this increased from 16% (day 5) to 55% (day 10). This coincided with a comparable decline in cells in fibroblastic clusters (1, 4, 7, 9), from 77% (day 5) to 34% (day 10). Thus, from days 5 to 10 after fracture there is a dramatic shift from an early fibrogenic callus to an endochondral one.

The four fibroblast clusters of day 10 were contiguous, with Cluster 7 being the most progenitor-like based on DEGs *Clec3b*, *Pi16* and *Ly6a* (Suppl Fig S3C). This cluster is highly similar to day 5/Cluster 6, with each comprising 6% of callus mesenchymal cells; the comparable proportion of progenitor-like cells on days 5 and 10 suggests maintenance of a progenitor pool during the early phases of callus formation. Adjacent to cluster 7 are clusters 1 and 9. Cluster 9 is a small (2.3% of cells) population of mixed/reparative fibroblasts (representative DEGs *Gzme, Cxcl12, Cxcl5, Sfrp1*). Notably, *Cxcl5* and *Sfrp1* were markers of the regenerative MPCs identified by Wang et al. (3). Cluster 1 is a large (16% of cells) population of tenocyte-like fibroblasts (*Tnmd*, *Abi3bp*, *Aspn*, *Ctsk*) that bridges the

UMAP space between progenitor-like cells (clusters 7, 9) and more differentiated fibroblasts (cluster 4). This cluster is much larger than the *Tnmd*+ cluster 9 from day 5 (2.6% of cells), indicating an expansion of this population. The significance of a population of cells in fracture callus that express the tenocyte marker *Tnmd* is unclear, although tenomodulin is expressed by tendon stem/progenitor cells (TSPCs), which are reported to have chondrogenic and osteogenic potential (15, 16). Cluster 4 appears to be a transitional fibroblastic cluster with a signature of matrix producing genes consistent with activated fibroblasts (DEGs *Ecm1, Tnn, Postn, Inhba*). This population has several marker genes (DEGs *Tnn*, *Postn*, *Aspn*) in common with the Osteo-X cells shown by Nookaew et al. (14) to be present on the periosteum of intact mouse long bones. Intriguingly, Osteo-X, like our Cluster 4, was closely associated with a cluster of tenocyte-like cells on one side, and pre-osteoblasts on the other, suggesting that cells on the periosteum of intact bone share a common differentiation path as cells in periosteal fracture callus.

Adjacent to Cluster 4 is the osteoblastic Cluster 5 (representative DEGs *Ifitm5*, *Serpinb6b, Creb3l1*, *Sp7*). A subset of cells in Cluster 4 also express mature osteoblastic genes *Dmp1* and *Bglap*. Notably, *Ifitm5* and *Serpinb6b* are highly specific Cluster 5. The specificity of *Ifitm5* for this cluster of callus cells is of clinical relevance, as mutations in *IFITM5* cause a range of osteogenesis imperfecta phenotypes (17-19). Moreover, mice that express mutant *Ifitm5* in osteochondral progenitor cells have impaired endochondral ossification (20), suggesting that this gene may play a role in endochondral fracture repair. On the other hand, a role for *Serpinb6b* in bone has not been reported. *Creb3l1/*OASIS is a transcription factor that directly regulates *Col1a1* expression (21), and CREB3L1 mutations are reported to also cause osteogenesis imperfecta. Of note, many cells with an osteoblastic signature (*Sp7*+/*Bglap*+/*Ibsp*+) fell outside of Cluster 5, indicating that the transcriptome of osteoblastic cells in the day 10 callus is heterogeneous.

The six clusters labeled as chondrocytes illustrate the diversity of this cell type in the day 10 fracture callus. While all share strong expression of classical markers *Col2a1*, *Acan* and *Alpl* (Suppl Fig S3J), they are marked by different DEGs. Cluster 2, which is adjacent to the osteoblastic Cluster 5, has the signature of mature hypertrophic chondrocytes based on DEGs *Col10a1, Mmp13* and *Ihh* (Suppl Fig S3C). Cluster 6 appears to be a proliferating chondrocyte population, with high expression of *Col10a1* and *Spp1*; 50% of cells in this cluster are in the G2M or S phase of the cell cycle. Cluster 0 is a large cluster marked by DEGs *Hapln1*, *Matn3*, *Col2a1* and *Col9a1*, all canonical chondrocyte genes. Cluster 3 is marked by DEGs *Ucma* and *Cnmd*. *Ucma* is expressed in chondrocytes in the developing skeleton and linked to osteogenic differentiation (22).

Regarding the targeting of proliferating 3.6Col1a1-lineage cells, the mature and hypertrophic chondrocytes in Clusters 0 and 2 were significantly depleted in callus from Experimental mice. In Control mice, cells from these two clusters comprised 39% of all mesenchymal cells, whereas in Experimental mice they comprised only 15% (Fig 3F). Notably, the proportion of cells in the osteoblastic Cluster 5 did not differ between groups. Because osteoblastic cells were in several clusters, we examined the number of cells co-expressing the canonical osteoblastic genes *Sp7*, *Bglap* and *Ibsp*, regardless of cluster. The proportion of these cells was reduced from 11% of mesenchymal cells in Control mice, to 3.8% in Experimental mice, consistent with the targeted depletion of osteoblasts. We next counted the number of cells for each cluster that were in the cell cycle (S or G2/M phases) in Control and Experimental groups (Suppl Fig S3D). The greatest reductions in proliferating cells with GCV treatment was observed in Clusters 0, 2, 5 and 6, comprised of mature and hypertrophic chondrocytes and osteoblasts, which is consistent with the selective ablation of proliferating osteochondral cells.

Finally, in contrast to the targeted reduction of osteochondral cells in Experimental mice, some of the day 10 fibroblast populations were significantly increased. The proportion of cells in Cluster 4 increased from 2.5% of cells in Control to 21% of cells in Experimental, indicating an expansion of this pool of transitional fibroblasts. In addition, the *Clec3b*+ progenitors of Cluster 7 increased from 2.6% in Control to 11.1% in Experimental. These findings suggest a compensatory increase in early osteochondral progenitors occurs when expansion of more mature osteochondral cells is blocked. In summary, scRNAseq indicates that ablation of proliferating 3.6Col1a1-lineage cells in the first 10 DPF leads to targeted depletion of osteochondral cells, and enrichment of less differentiated fibroblastic cells.

Blocking gap junction-mediated intercellular communication (GJIC) does not rescue impaired fracture healing in Osx-CreERT2, Ocn-Cre, or Dmp1-CreERT2; ROSA-TK mice

A feature of the TK/GCV model that has been noted is a bystander effect (23, 24). This occurs when a TK-expressing cell is linked to other cells via gap junctions, which facilitates the passage of the toxic mono-phosphorylated GCV (mediated only by TK enzymatic activity) to neighboring cells that may not express TK. This modified GCV can then be further phosphorylated and lead to apoptosis in a proliferating cell that never expressed TK. To test whether this by-stander effect contributes to the impaired fracture healing seen in ROSA-TK mice, we treated Osx-CreERT2; ROSA-TK, Ocn-Cre; ROSA-TK, and Dmp1-CreERT2; ROSA-TK mice and their respective controls with the gap junction

blocker Carbenoxolone (CBX) once daily (along with GCV) following full femur fracture. Based on microCT analysis at 14 DPF, Cre+/TK+ mice had significantly diminished callus bone volume compared to Cre-/TK+ control mice, and this was not different in CBX-treated mice than in mice that were not treated with CBX (Suppl Fig S11). Thus, inhibiting gap junction communication did not rescue impaired fracture healing in any of the ROSA-TK models. This result indicates that the impaired healing observed when early and mature proliferating osteoblasts are ablated is not due to a gap junction-mediated intercellular bystander effect but is due to the ablation of actively proliferating Osterix-, Osteocalcin-, or Dmp1-expressing cells during fracture healing.

Ablation of proliferating Adq-expressing cells modestly affects fracture healing

Fracture callus is predominantly derived from periosteal skeletal progenitors, albeit with some contribution from bone marrow cells (25). Adiponectin (Adq) is expressed in osteoprogenitor cells in bone marrow, but is not expressed by periosteal cells (8). Therefore, we evaluated fracture healing in Adq-CreERT2; ROSA-TK mice as a negative control, i.e., with the hypothesis that fracture healing would be minimally affected. To activate Cre, mice were dosed with TMX 2 weeks prior to fracture. Cre-/TK+ (control) and Cre+/TK+ (exptl) mice were treated with TMX and GCV and fracture healing was evaluated at 14 days post-fracture (Suppl Fig 12A).

Radiographic analysis indicated that 80% of control mice had callus bridging of both cortices and 90% had bridged at least one cortex, compared to 38% and 75%, respectively, in Cre+/TK+ mice, suggesting delayed bridging in the latter (Suppl Fig 12B). Histologically, fracture calluses from Cre+/TK+ mice were marginally smaller than Cre-/TK+ controls (-25%, $p=0.11$) although callus composition was proportionately normal in Cre+/TK+ mice (Suppl Fig 10C). MicroCT analysis showed that callus bone volume was marginally less in Cre+/TK+ mice than in Cre- controls (-20%, $p=0.14$), while callus BMD was not affected (Suppl Fig 12D). In comparison, Osx-CreERT2;ROSA-TK mice have 65% lower callus bone volume compared to their controls ($p<0.0001$; Fig 4E). These results indicate a modest effect on fracture callus formation in mice wherein proliferating adiponectin-expressing cells were ablated, consistent with targeting of bone marrow skeletal progenitors but not periosteal progenitors. This finding supports that Cre+/TK+ mice treated with GCV do not inherently have impaired fracture healing.

Supplemental Methods

Chemicals and Reagents: Tamoxifen (TMX, T5648), corn oil (C8267), and Ganciclovir (GCV, G2536) were from Millipore Sigma. Goat Anti-Rabbit Immunoglobulins/HRP (affinity isolated, P0448) was

from Agilent Dako. Diaminobenzidine (DAB) chromogen (SK-4105) and Hematoxylin QS Counterstain (H-3404) were from Vector Laboratories. ReadyProbes Mouse-on-Mouse IgG Blocking Solution (R37621), Goat anti-Mouse IgG (H+L), Superclonal™ Recombinant Secondary Antibody, Alexa Fluor™ 488 (A28175), Goat anti-Rabbit IgG (H+L) Highly Cross-Adsorbed Secondary Antibody, Alexa Fluor™ 546 (A11035), and ProLong™ Glass Antifade Mountant with NucBlue™ Stain (P36981) were from ThermoFisher. Anti-Osteocalcin (A93876) and Anti-Osterix (22552) were from Abcam. Anti-PCNA (2586) was from Cell Signaling Technology. 22μm sterile filters (09-720-004) were from Fisher Scientific. Collagenase Type II (L5004176) was from Worthington. Pronase (53702), Trypan blue (T8154), and High Glucose DMEM (D6429) were from Sigma. LIVE/DEAD™ Viability/Cytotoxicity Kit (L3224) was from Invitrogen. 40μm SureStrain™ Premium Cell Strainers (C4040) were from MTCBio. MS Columns (130-042-201) were from Miltenyi Biotec. The Corded Dremel (5EEU5) was from Grainger. The Dremel bit (2901A247) and the tungsten guide wire (3775K37) were from McMaster Carr. Hypodermic tubing (304H24TW) was from Microgroup. 4-0 nylon sutures (1854G) were from Ethicon. Anti-Thymidine Kinase Antibody was a gift from William Summers (Yale).

Col1-TK Mice: Breeders were rederived from cryopreserved embryos gifted by Drs. O'Brien and Jilka (26) (UAMS). A total of 90 mice, age 12-wks were used. Transgenic 3.6Col1a1-TK (Col1-TK) mice were used to target proliferating (pre)osteoblasts, wherein expression of herpes simplex virus thymidine kinase (TK) in early osteoblasts is driven by the 3.6 kilobase rat *Col1a1* promoter (26, 27). Heterozygous female Col1-TK mice were bred to wild-type males to generate wild-type (WT) controls or Col1-TK (TK) experimental mice of both sexes. Mice were administered ganciclovir (GCV, 8 mg/kg i.p., 1/d) starting on the day of fracture and were euthanized at 5-, 10-, or 21-days post-fracture.

Fracture Model: An established model of mid-diaphyseal femur fracture was used (27, 28). Briefly, following administration of an analgesic (Buprenorphine SR, 1 mg/kg, s.c), mice were anesthetized (1-3% isofluorane) and a 5 mm incision was made above the knee, exposing the distal femoral condyles. The medullary canal was reamed out, and a 1.5-inch-long tungsten guide wire was inserted into the marrow space. To create a mid-point femur fracture, a transverse force was applied using a three-point bending set-up (DynaMight 8841; Instron). A 24-gauge, 1-inch hypodermic stainless-steel tube was inserted over the guidewire to stabilize the fracture. The guidewire was removed, the tube clipped to length, and the incision closed using 4-0 nylon suture. Pin placement was confirmed with radiography

immediately after surgery. This model heals via secondary fracture healing, with intramembranous bone formation at the periphery and endochondral bone formation within the fracture gap.

Cell Isolation for Single Cell RNA Sequencing:

For scRNAseq, Col1-TK mice (n=3/time/treatment) were fractured and treated with either water (Control) or GCV (Exptl) for 5- or 10-days post-fracture. Following euthanasia, the fracture site was exposed, and total callus tissue scraped away from cortical bone using a fresh scalpel. Tissue was minced in 1 mL of 22 mm sterile-filtered digestion buffer comprised of 1663 U/mL Collagenase Type II and 1814 PUK/mL Pronase in High Glucose DMEM with 8% FBS. After digestion for 1 hr at 37ºC, cells were pelleted and re-suspended in 0.01% BSA in sterile PBS. Cells were filtered through a 40 mm filter, and dead cells were removed using an MS Column. Trypan Blue-stained cells were counted and viability estimated using a Live/Dead kit. Twelve samples (one per mouse, no pooling) comprising 40,000 cells/sample with 70% viability were sent for sequencing.

Single Cell RNA Sequencing and Analysis:

Twelve single cell suspensions were transferred to the Genome Technology Access Center at Washington University for library preparation using the 10X Genomics Chromium Single Cell 3' Reagent Kit (v3.1). Sequencing was done in a single lane of Illumina Novaseq (avg. 8,700 cells per sample; range: 4170-16,323). A mean of 74,692 reads per cell were sequenced, and mapped to a median of 2,770 genes per cell, with overall coverage of 61% of the transcriptome. An average of 23,140 genes were detected per sample. Following standard normalization and filtering, data from 104,740 cells (Day 5: Control 32,322 cells; GCV 28,383; Day 10: Control 23,278; GCV: 20,307) were available for analysis.

Secondary analysis was done using Seurat Version 4.4.0 in R (29). All cells from each time point, independent of treatment, were subjected to unbiased clustering and were visualized on UMAP plots. For cluster annotation, we used several computational tools. First, the top 50 differentially expressed genes (DEGs) from each cluster were entered into Comprehensive Multi-Omics Platform for Biological Interpretation (COMPBIO, Washington University) to identify the top biological themes and components for each cluster. Further analysis was performed by using ChatGPT5, following a validated approach that was recently applied to analysis of fracture callus (3, 30). We used the prompt "Identify cell type within musculoskeletal tissue using the following markers [list of top 10-20 DEGs]". Results of these two approaches were synthesized along with recent data from the literature (2, 3, 5, 14) to select cell cluster names and representative genes. Following annotation, clusters comprised of predominantly

mesenchymal lineage cells were digitally selected and re-clustered for better cell identity resolution. The top 50 DEGs from the mesenchymal-only clusters were also input into COMPBIO for annotation. Seurat in R was used for cell subsetting. Threshold cutoffs for *Sp7*/Osterix and *Bglap*/Osteocalcin used were 90% of the mean gene expression of mesenchymal cells only from control water-treated mice from each time point. Cell cycle stage was determined based on canonical gene expression profiles as described (31). Cells are assigned to either G1 phase (stagnant, functional phase), S Phase (actively replicating DNA in preparation for cell division), or G2M phase (preparing for and going through mitosis). Cells assigned to either the S or G2M phase are judged to be actively proliferating.

Generation and Validation of a ROSA-TK Mouse Line:

To generate a Cre-inducible mouse (ROSA26-LSL-HSV-TK, or 'ROSA-TK') for targeted ablation of proliferating cells, we utilized a truncated herpes simplex virus thymidine kinase allele (HSV-deltaTK, Addgene 114273) to circumvent male sterility caused by the full sequence TK while retaining enzymatic function (32). The HSV-deltaTK knock-in targeting vector contained a CAG promoter, followed by floxed stop cassette (LSL), the HSV-deltaTK allele, a P2A self-cleaving site, and finally an mCherry allele (Suppl Fig S4). The plasmid was synthesized by the Genome Engineering and Stem Cell Core (Washington University). The floxed stop cassette allows for tissue-specific expression of HSV-deltaTK and mCherry upon Cre-mediated excision. The P2A site allows for bicistronic expression of the HSV-deltaTK and mCherry genes. ROSA26 specific TALENs were designed using the ZiFit targeter (http://zifit.partners.org) and ROSA26 TALENs binding sites as follows:

1) 5' ROSA26 TALEN binding site: 5' TCCCTCGTGATCTGCAACTCC 3'
2) 3' ROSA26 TALEN binding site: 5' GGGCGGGAGTCTTCTGGGCA 3'

The TALEN kit used for TALE assembly was a gift from Keith Joung (Addgene kit # 1000000017). DNA fragments encoding ROSA TALEN repeat arrays were cloned into plasmid pJDS71. ROSA TALENs plasmids were linearized for *in vitro* transcription with EcoRI and TALENs RNA was synthesized using the mMessage mMachine T7 Ultra kit (Ambion) and purified with Megaclear columns (Ambion). The knock-in cassette was introduced into the mouse genome via pronuclear injection of *in vitro* transcribed TALENs RNA and ROSA donor DNA into C57BL/6J;CBA hybrid oocytes which were then implanted into hosts dams (Mouse Genetics Core, Washington University). Six offspring with correctly targeted alleles were identified by long-range PCR using ROSA specific primers external to the homology arms (not shown). These six potential founders (F0 generation) were bred to WT C57Bl/6 mice. Presence of the mutant allele was demonstrated in the six potential founders

(F0) using standard PCR (Suppl Fig S5A-B). F1 pups were screened to confirm transmission of the mutant allele from founders to offspring (Suppl Fig S5C).

Two confirmed ROSA- TK founders (354-7 and 356-4) were crossed to tamoxifen-inducible Osx-CreERT2 mice and validation was assessed in F1 mice by three methods. First, we confirmed tamoxifen-dependent excision of the stop cassette only in Cre+ mice (Suppl Fig S5D-E). Second, we used IHC to confirm Cre-dependent TK protein expression in trabecular osteoblasts only in Osx-CreERT2+; ROSA-TK+ mice, supporting that excision of the stop cassette is sufficient to drive TK expression in the targeted cells (Suppl Fig S5F). Third, we demonstrated nonunion fractures only in Cre+/TK+ mice (Fig 4, Suppl Fig 6). These two founders were used to establish the ROSA-TK lines, and all mice for subsequent studies were derived therefrom. Each founder line was kept separate to ensure reproducibility of phenotypes independent of founder. (Note that we did not detect mCherry fluorescence on frozen sections, but confirmed mCherry expression using IHC (not shown).)

Maintenance of Cre Lines and Generation of Cre;TK Mice:

Osx-CreERT2 (from Dr. Kronenberg (33), MGI:4829803), Dmp1-CreERT2 (from Dr. Divieti Pajevic (34), MGI:5792965) and Ocn-Cre (Jackson Laboratory, #19509) mice were acquired and maintained by breeding Cre-positive males to wild type C57Bl/6 females (Jackson Laboratory, #000664). Cre-positive males were bred with ROSA-TK females to generate control (Cre-;TK-, Cre-;TK+, Cre+;TK-) and experimental (Cre+;TK+) littermates. When possible, Cre+;TK+ males were bred to Cre-;TK+ females to generate control and experimental littermates with homozygous TK+/+ genotypes.

Genotyping:

For 3.6ColTK mice, genotyping was done by Transnetyx using toe biopsies for the absence or presence of the puromycin selection cassette:

- Fwd: 5' –GCGGTGTTCGCCGAGAT– 3'; Rev: 5' –GAGGCCTTCCATCTGTTGCT– 3'

For ROSA-TK mice, primers targeting the wild-type ROSA allele or the CAG promoter were used to distinguish wild-type and mutant (TK+) alleles:

- Wild-type: For: 5' – GTTATCAGTAAGGGAGCTGCAGTGGAGTAG – 3'; Rev: 5' – CCGAAAATCTGTGGGAAGTCTTGTCCCTCC – 3'
- TK+: For: 5' - GTTATCAGTAAGGGAGCTGCAGTGGAGTAG - 3'; Rev: 5' - CTCCACCCATTGACGTCAATGGAAAGTCCC - 3'

Following establishment of the ROSA-TK line, genotyping was done by Transnetyx using toe biopsies for the wild-type ROSA allele or the inclusion of the mCherry (TK+) sequence:

- Wild-type: For: 5' – TTCCCTCGTGATCTGCAACTC – 3'; Rev: 5' – CTTTAAGCCTGCCCAGAAGACT – 3'
- mCherry: For: 5' – AGCGCGTGATGAACTTCGA – 3'; Rev: 5' – GCGCAGCTTCACCTTGTAGAT – 3'

For Stop cassette genotyping in Osx-CreERT2; ROSA-TK mice, mice were given three doses of TMX across 5 days. Tail snips were taken prior to and after full TMX treatment for genotyping to visualize excision of the stop cassette:

- Fwd: 5' – GAGGGCCTTCGTGCGTC – 3'; Rev – 5' CATCGGCTCGGGTACGTAGA – 3'

For Cre allele genotyping, genotyping was done by Transnetyx using toe biopsies:

- Osx-CreERT2 and Dmp1-CreERT2: Fwd: 5' - TGCGCCTGCTGGAAGAT -3'; Rev: 5' – GGTTGGCAGCTCTCATGTCT – 3'
- Ocn-Cre: Fwd: 5' – TTAATCCATATTGGCAGAACGAAAACG – 3'; Rev: 5' – CAGGCTAAGTGCCTTCTCTACA – 3'

Radiographic Evaluation:

Lateral radiographs were taken weekly to confirm proper positioning of the intramedullary pin and to monitor healing (Faxitron UltraFocus100). Radiographs from 14 or 21 weeks post-fracture were blindly scored using the modified RUST (radiographic union score for tibial fracture) method adapted for mouse femurs (35). From the lateral projection, we scored the anterior and posterior cortices on a 4-point scale (1=fracture line visible, no callus formation; 2=fracture line visible, visible callus; 3=fracture line visible, callus fully bridged; 4=fracture line not visible, callus fully bridged) and summed the two scores (possible range 2 to 8). Because the fracture lines were still visible on both cortices at these timepoints, the maximum score in our study was 6. Mice were excluded if the fixation pin displaced during healing.

Micro-Computed Tomography:

Fractured and intact contralateral femurs were fixed in 10% non-buffered formalin for 16 hours, rinsed with PBS and stored in 70% ethanol. Femurs were scanned *ex vivo* using microCT (VivaCT 40, Scanco, 10.5μm voxel size, 55kV, 145μA, 300ms integration time). Conventional analysis of a 600-slice ROI centered on the mid-point of the fracture line was done, as described (27). A single lower threshold (200 per mille, contour peel 2) was used to determine bone volume (BV, mm^3), tissue volume (TV, mm^3),

volumetric bone mineral density (vBMD, mg HA/cm^3), and tissue mineral density (TMD, mg HA/cm^3), inclusive of callus bone and cortical bone. Cortical parameters from the mid-diaphysis of intact limbs were similarly analyzed; the population mean (dotted line) and standard deviation (grey shading) are shown overlaying data from fractured limbs. To isolate callus bone in fractured femurs, a two-threshold method was used: 150 and 460 per mille (contour peel of 0) to segment total bone and cortical bone, respectively.

Cortical bone volume (CBV_{460}) was subtracted from total bone volume (TBV_{150}) to yield callus bone volume (mm^3). Following microCT, femurs were decalcified and processed for histology.

Histology and Analysis of Callus Composition:

Femurs were decalcified in 14% EDTA, pH 7.0 for 2 weeks, with EDTA changed every 3-4 days. Femurs were embedded in paraffin wax and 5 µm thick, longitudinal sagittal sections through the middle of the fracture callus were mounted on glass slides. Slides were deparaffinized in xylenes, rehydrated in graded ethanols and stained by hematoxylin and eosin (H&E), and picrosirius red and alcian blue (PSRAB) to visualize collagen-rich woven bone and proteoglycan-rich cartilage, respectively. Slides were imaged at 20× on a Nanozoomer slide scanner (Hamamatsu Photonics). Callus composition was quantified in NDP View 2 software (Hamamatsu) by drawing custom contours around different tissue components (see example in Suppl Fig S1C). Total callus area was the area of both sides of the periosteal callus, excluding any intact cortical bone or bone marrow. Cartilage was identified by Alcian blue stained areas and woven bone was identified by Picrosirius red stained areas. Fibrous tissue was identified as a mix of the two stains that did not morphologically resemble either cartilage or bone.

Thymidine Kinase (TK) Immunohistochemistry:

Immunohistochemistry was used to visualize HSV-TK expression in the callus region of histological sections. Following deparaffinization, endogenous peroxidase activity was blocked using 3% hydrogen peroxide. Sections were blocked with 10% goat sera + 1% bovine serum albumin for 1 hr at room temperature (RT). Anti-TK antibody (1:1000, gift from William Summers (36)) was diluted in 2% goat sera and incubated overnight at 4C. Anti-rabbit HRP (1:500) was diluted in 2% goat sera and incubated for 1 hr at RT. All samples were incubated with diaminobenzidine (DAB) chromogen for 30 sec and rinsed in water to stop the reaction. Sections were counterstained with hematoxylin for 30 sec and imaged at 20× as above.

Immunohistochemistry DAB Quantification:

Following immunohistochemistry where DAB chromogen was used, particularly for PCNA and TK staining, the FIJI package of ImageJ (NIH) was used for quantification of DAB signal. Quantification and analysis were performed for all TK-positive mice, including control (H2O-treated) and experimental (GCV-treated) mice at 5- and 10-days post fracture (DPF). High magnification fields (15x) of each corner of the fracture callus (top right, top left, bottom right, & bottom left) were cropped for both TK and PCNA images for each sample. The tissues observed in these fields consisted mainly of woven bone in the fracture callus and varying amounts of cartilage, periosteum, muscle, fibrous tissue, and cortical bone depending on group and sample. Representative images for each group are shown in Figure S2H and S3G. An example of the workflow for the quantification is shown in Figure S14. The high magnification images were imported into FIJI, where the image processing option "Colour Deconvolution" was performed using H DAB vectors to obtain the DAB channel image. A threshold of 0-100 (255 max) was then applied to the DAB channel image, based on what visually appeared to be true positive DAB signal. This outputs a black and white image, where white indicates positive signal. The black and white images were used to count the number of cells using the "Analyze Particles" option under analyze in FIJI. The constraint for cell area size was set to 20-infinity pixel^2 and the cell circularity was set to 0.20-1.00 to count only cells and ignore background artifact. The total number of cells counted for all 4 quadrants of the fracture callus was summed for each sample and subsequently used for the statistical analysis in GraphPad Prism. Due to an uneven number of samples between groups, Welch's t test was used.

Co-Immunofluorescence:

Antigen retrieval was done with boiling 10mM citrate buffer and overnight incubation at 60C. Sections were permeabilized with 0.3% Triton-X 100, blocked with 10% goat serum for 1 hr at RT, and endogenous mouse IgG was blocked with Mouse-on-Mouse IgG Blocking Solution for 1 hr at RT. Sections were stained with either anti-PCNA (1:500) and anti-Osterix (1:150) or Anti-PCNA and anti-Osteocalcin (1:500) diluted in 1.5% goat serum overnight at 4C. 1:200 anti-mouse 488 (A28175) and 1:200 anti-rabbit 546 were diluted in 1.5% goat serum and incubated at RT for 1.5 hr. Sections were mounted and coverslipped with Prolong Glass with DAPI. PCNA is expressed during the S phase of the cell cycle where it interacts with DNA polymerase complexes to increase processivity by holding DNA polymerase onto the DNA strand (37). When compared to the incorporation of BrdU in rat fracture

model as a marker for DNA synthesis and cell proliferation, PCNA staining closely mimicked BrdU incorporation, supporting its use in detecting proliferating cells (38).

Co-Immunofluorescence Imaging and Quantification:

Fluorescent slides were imaged at 20× (Nanozoomer). QuPATH v0.4.4 (39) was used to quantify PCNA+ osteoblasts. Briefly, each half of the callus was manually contoured and Positive Cell Detection on the DAPI channel was used to identify the total number of nuclei. Single Measurement Classifiers were made for the PCNA and Osterix (Osx) signals individually, with thresholds based on maximum nuclear intensities for each channel. A Composite Measurement Classifier was created from the individual classifiers to identify PCNA-;Osx-, PCNA+;Osx-, PCNA-;Osx+, and PCNA+;Osx+ nuclei. Percent of proliferating osterix-expressing cells was calculated as the number of PCNA+;Osx+ cells divided by the total number of Osx+ nuclei. Similarly for Osteocalcin (Ocn)-stained sections, Positive Cell Detection on the DAPI channel was used to identify the total number of nuclei and the cell body was identified using cell expansion of 3 μm for 3-day and 4 μm for 5- and 10-day post-fracture samples. Osteocalcin thresholding was based on maximum cell intensity. Percent proliferating Ocn-expressing cells was calculated as the number of PCNA+;Ocn+ cells divided by the total number of Ocn+ cells. The two halves of each callus were averaged together to determine the rate of osteoblast proliferation throughout the entire callus (n=3 mice/timepoint).

Statistics:

Experimenters were blinded to animal genotype and treatment during data collection and analysis. Analyses were carried out in GraphPad Prism 9.0. Data were tested for normality prior to analysis. Data were compared with Chi-Squared tests, unpaired two-tailed t-tests, one-way ANOVA with Holm-Sidak post-hoc correction, two-way ANOVA with Holm-Sidak post-hoc correction, or non-parametric Kruskal-Wallis multiple comparison tests, as indicated in figure legends. ANOVA tables are provided as a supplemental excel file for main variable effects. A p-value of <0.05 was used as a threshold for statistical significance. Graphs show means with error bars indicating SD.

References Cited in Supplement

1. Leinroth AP, et al. Identification of distinct non-myogenic skeletal-muscle-resident mesenchymal cell populations. *Cell Rep.* 2022;39(6):110785.

2. Novak S, et al. Endothelial to mesenchymal Notch signaling regulates skeletal repair. *JCI Insight.* 2024;9(12).
3. Wang H, et al. Integrating spatial and single-cell transcriptomics to characterize mouse long bone fracture healing process. *Commun Biol.* 2025;8(1):887.
4. Miedel EL, et al. Type III collagen modulates fracture callus bone formation and early remodeling. *J Orthop Res.* 2015;33(5):675-84.
5. Yao L, et al. Activin A marks a novel progenitor cell population during fracture healing and reveals a therapeutic strategy. *Elife.* 2023;12.
6. Mayr CH, et al. Sfrp1 inhibits lung fibroblast invasion during transition to injury-induced myofibroblasts. *Eur Respir J.* 2024;63(2).
7. Yan M, et al. KIF26B Silencing Prevents Osseous Transdifferentiation of Progenitor/Stem Cells and Attenuates Ectopic Calcification in a Murine Model. *J Bone Miner Res.* 2022;37(2):349-68.
8. Jeffery EC, et al. Bone marrow and periosteal skeletal stem/progenitor cells make distinct contributions to bone maintenance and repair. *Cell stem cell.* 2022;29(11):1547-61 e6.
9. Matsushita Y, et al. A Wnt-mediated transformation of the bone marrow stromal cell identity orchestrates skeletal regeneration. *Nat Commun.* 2020;11(1):332.
10. Pakshir P, et al. The myofibroblast at a glance. *Journal of cell science.* 2020;133(13).
11. Lopez-Antona I, et al. Dynamic regulation of myofibroblast phenotype in cellular senescence. *Aging Cell.* 2022;21(4):e13580.
12. McDonald SJ, et al. Transient expression of myofibroblast-like cells in rat rib fracture callus. *Acta orthopaedica.* 2012;83(1):93-8.
13. Yokota T, et al. Type V Collagen in Scar Tissue Regulates the Size of Scar after Heart Injury. *Cell.* 2020;182(3):545-62 e23.
14. Nookaew I, et al. Refining the identity of mesenchymal cell types associated with murine periosteal and endosteal bone. *J Biol Chem.* 2024;300(4):107158.
15. Bi Y, et al. Identification of tendon stem/progenitor cells and the role of the extracellular matrix in their niche. *Nat Med.* 2007;13(10):1219-27.
16. Asai S, et al. Tendon progenitor cells in injured tendons have strong chondrogenic potential: the CD105-negative subpopulation induces chondrogenic degeneration. *Stem cells.* 2014;32(12):3266-77.
17. Rauch F, et al. Osteogenesis imperfecta type V: marked phenotypic variability despite the presence of the IFITM5 c.-14C>T mutation in all patients. *J Med Genet.* 2013;50(1):21-4.

18. Farber CR, et al. A novel IFITM5 mutation in severe atypical osteogenesis imperfecta type VI impairs osteoblast production of pigment epithelium-derived factor. *J Bone Miner Res.* 2014;29(6):1402-11.
19. Rodriguez Celin M, et al. Uncommon IFITM5 mutation associated with severe skeletal deformity in osteogenesis imperfecta. *Ann Hum Genet.* 2018;82(6):477-81.
20. Marom R, et al. The IFITM5 mutation in osteogenesis imperfecta type V is associated with an ERK/SOX9-dependent osteoprogenitor differentiation defect. *J Clin Invest.* 2024;134(15).
21. Murakami T, et al. Signalling mediated by the endoplasmic reticulum stress transducer OASIS is involved in bone formation. *Nature cell biology.* 2009;11(10):1205-11.
22. Surmann-Schmitt C, et al. Ucma, a novel secreted cartilage-specific protein with implications in osteogenesis. *J Biol Chem.* 2008;283(11):7082-93.
23. Mesnil M, et al. Bystander effect in herpes simplex virus-thymidine kinase/ganciclovir cancer gene therapy: role of gap-junctional intercellular communication. *Cancer research.* 2000;60(15):3989-99.
24. Andrade-Rozental AF, et al. Gap junctions: the "kiss of death" and the "kiss of life". *Brain Res Brain Res Rev.* 2000;32(1):308-15.
25. Colnot C. Skeletal cell fate decisions within periosteum and bone marrow during bone regeneration. *J Bone Miner Res.* 2009;24(2):274-82.
26. Jilka RL, et al. Intermittent PTH stimulates periosteal bone formation by actions on post-mitotic preosteoblasts. *Bone.* 2009;44(2):275-86.
27. Hixon KR, et al. Ablation of Proliferating Osteoblast Lineage Cells After Fracture Leads to Atrophic Nonunion in a Mouse Model. *J Bone Miner Res.* 2021;36(11):2243-57.
28. McBride-Gagyi SH, et al. Bmp2 conditional knockout in osteoblasts and endothelial cells does not impair bone formation after injury or mechanical loading in adult mice. *Bone.* 2015;81:533-43.
29. Hao Y, et al. Integrated analysis of multimodal single-cell data. *Cell.* 2021;184(13):3573-87 e29.
30. Hou W, et al. Assessing GPT-4 for cell type annotation in single-cell RNA-seq analysis. *Nat Methods.* 2024;21(8):1462-5.
31. Tirosh I, et al. Dissecting the multicellular ecosystem of metastatic melanoma by single-cell RNA-seq. *Science.* 2016;352(6282):189-96.
32. Salomon B, et al. A truncated herpes simplex virus thymidine kinase phosphorylates thymidine and nucleoside analogs and does not cause sterility in transgenic mice. *Mol Cell Biol.* 1995;15(10):5322-8.

33. Maes C, et al. Osteoblast precursors, but not mature osteoblasts, move into developing and fractured bones along with invading blood vessels. *Dev Cell.* 2010;19(2):329-44.

34. Powell WF, Jr., et al. Targeted ablation of the PTH/PTHrP receptor in osteocytes impairs bone structure and homeostatic calcemic responses. *J Endocrinol.* 2011;209(1):21-32.

35. Alentado VJ, et al. Validation of the modified radiographic union score for tibia fractures (mRUST) in murine femoral fractures. *Frontiers in endocrinology.* 2022;13:911058.

36. Smiley WR, et al. Establishment of parameters for optimal transduction efficiency and antitumor effects with purified high-titer HSV-TK retroviral vector in established solid tumors. *Hum Gene Ther.* 1997;8(8):965-77.

37. Kelman Z. PCNA: structure, functions and interactions. *Oncogene.* 1997;14(6):629-40.

38. Wildemann B, et al. Cell proliferation and differentiation during fracture healing are influenced by locally applied IGF-I and TGF-beta1: comparison of two proliferation markers, PCNA and BrdU. *J Biomed Mater Res B Appl Biomater.* 2003;65(1):150-6.

39. Bankhead P, et al. QuPath: Open source software for digital pathology image analysis. *Sci Rep.* 2017;7(1):16878.

Supplemental Figure S1. Col1-TK mice (This figure supplements main Figure 1.)

A. Radiographic scoring

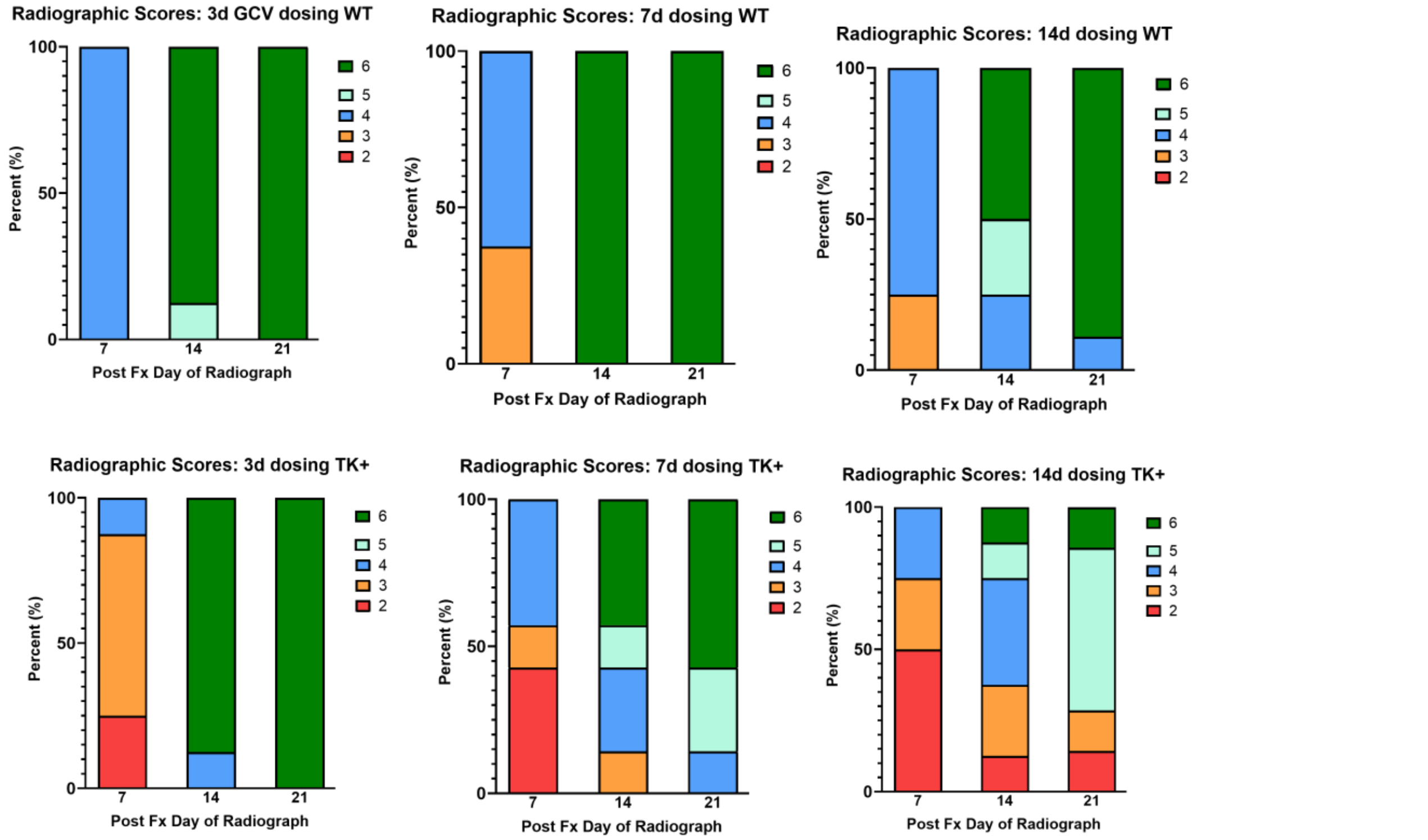

B. MicroCT parameters at 21 DPF. (ROI includes callus and cortical bone.)

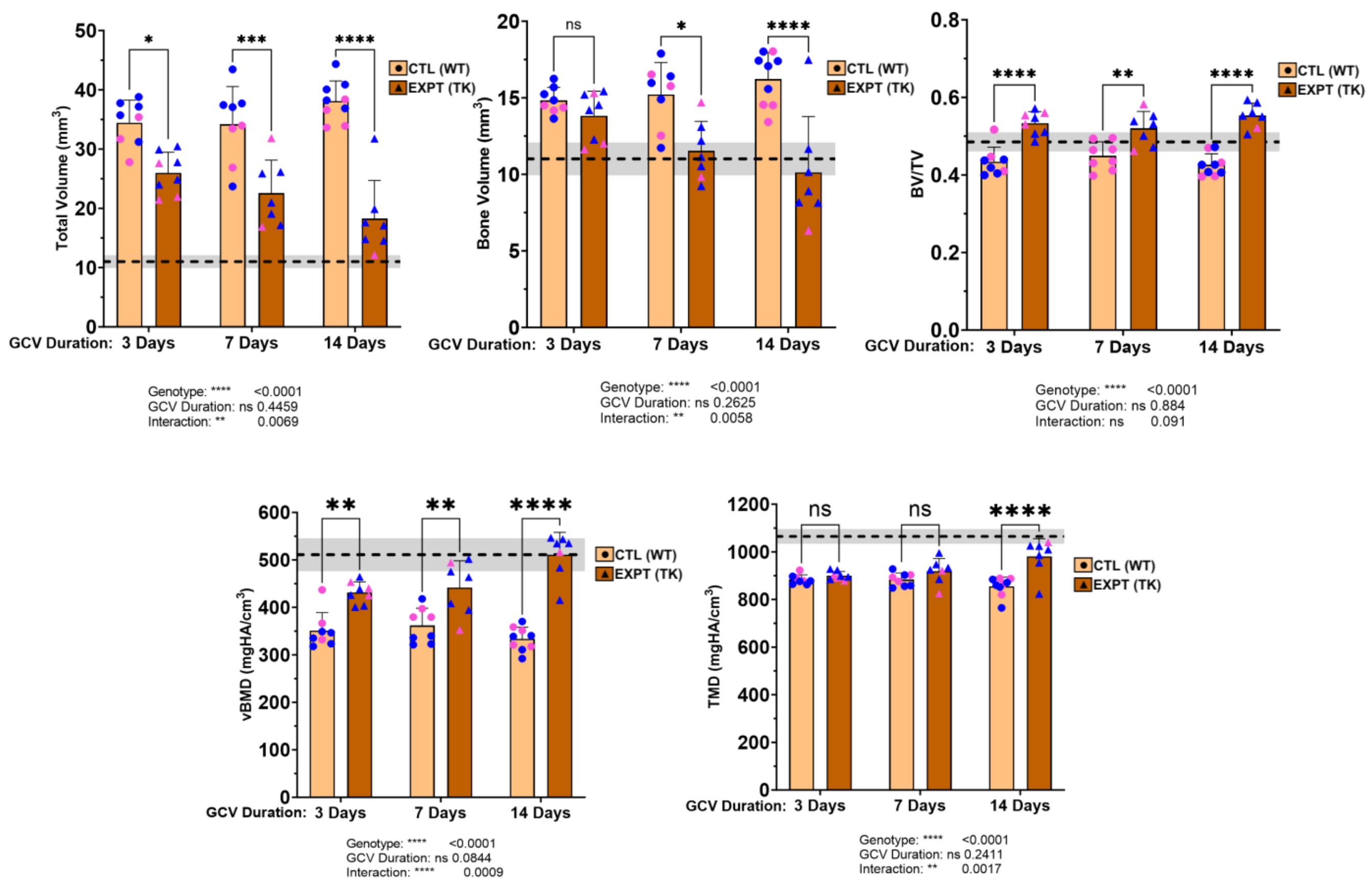

Figure S1. (A) Radiographic scoring for Col1TK mice by dosing duration at 7, 14 and 21 days post-fracture (DPF) (n=7-9). (Score 2=neither cortex bridged; 6=both cortices bridged). **(B)** microCT parameters from the callus region at 21 DPF (incl. callus + cortical bone). (Dashed lines indicate avg. value of contralateral intact femurs; shading denotes +/- SD). (Graphs in B depict mean ± SD; statistical differences determined by Two-Way ANOVA)

Supplemental Figure S1. (cont.) Col1-TK mice.

C. Histological callus composition at 21 DPF

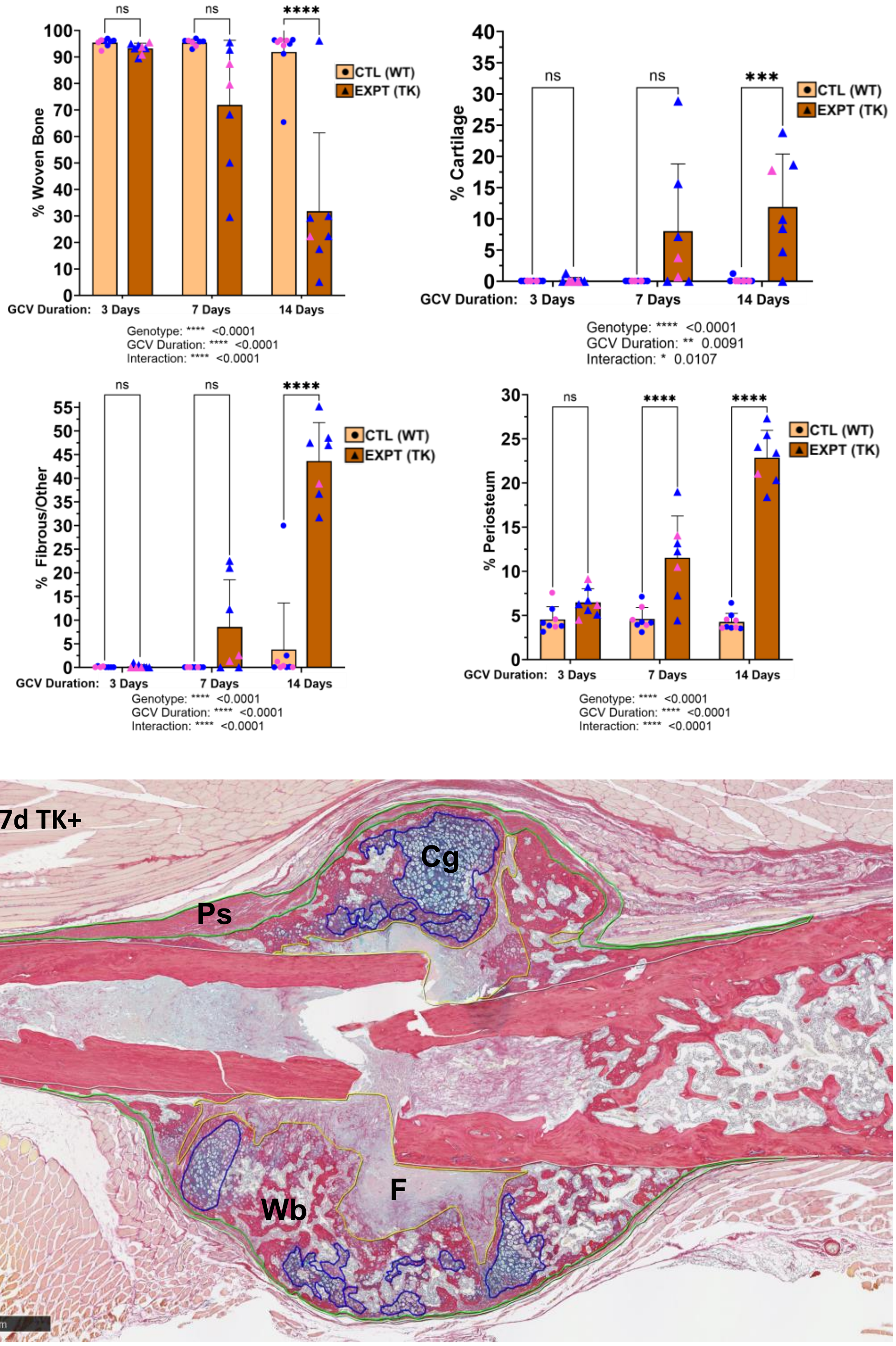

Figure S1. (C) Histological callus composition at 21 DPF was determined from sections stained by picrosirius red and alcian blue (PSRAB). Image is representative section from Col1-TK mouse treated for 7 days with GCV. (Cg: cartilage, Wb: woven bone, F: fibrous tissue, Ps: periosteum.) (Graphs depict mean ± SD; statistical differences determined by Fisher’s Exact Test)

Supplemental Figure S1. (cont) Col1-TK mice.

D. Hematoxylin & Eosin staining at 21DPF

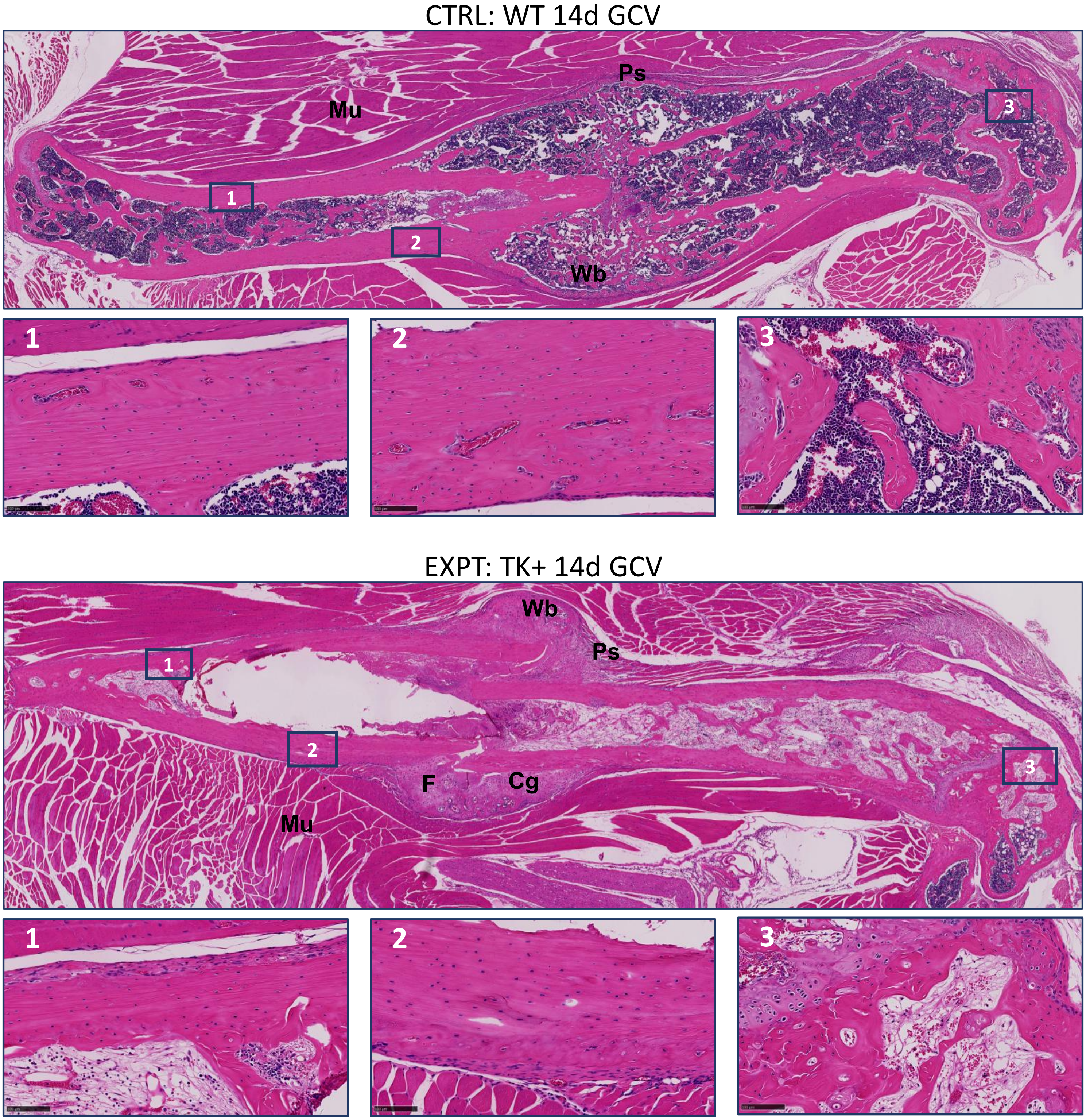

Figure S1. (D) Representative Hematoxylin & Eosin (H&E) stained slides for control (top) and experimental (bottom) groups receiving GCV for 14 days at 21DPF. H&E staining shows no off-target effect from GCV administration, with osteocytes present all along distal and proximal cortical bone and near fracture callus. (Cg: cartilage, Wb: woven bone, F: fibrous tissue, Ps: periosteum, Mu: muscle)

Supplemental Figure S2. Col1-TK Day 5 (This figure supplements main Figure 2.)

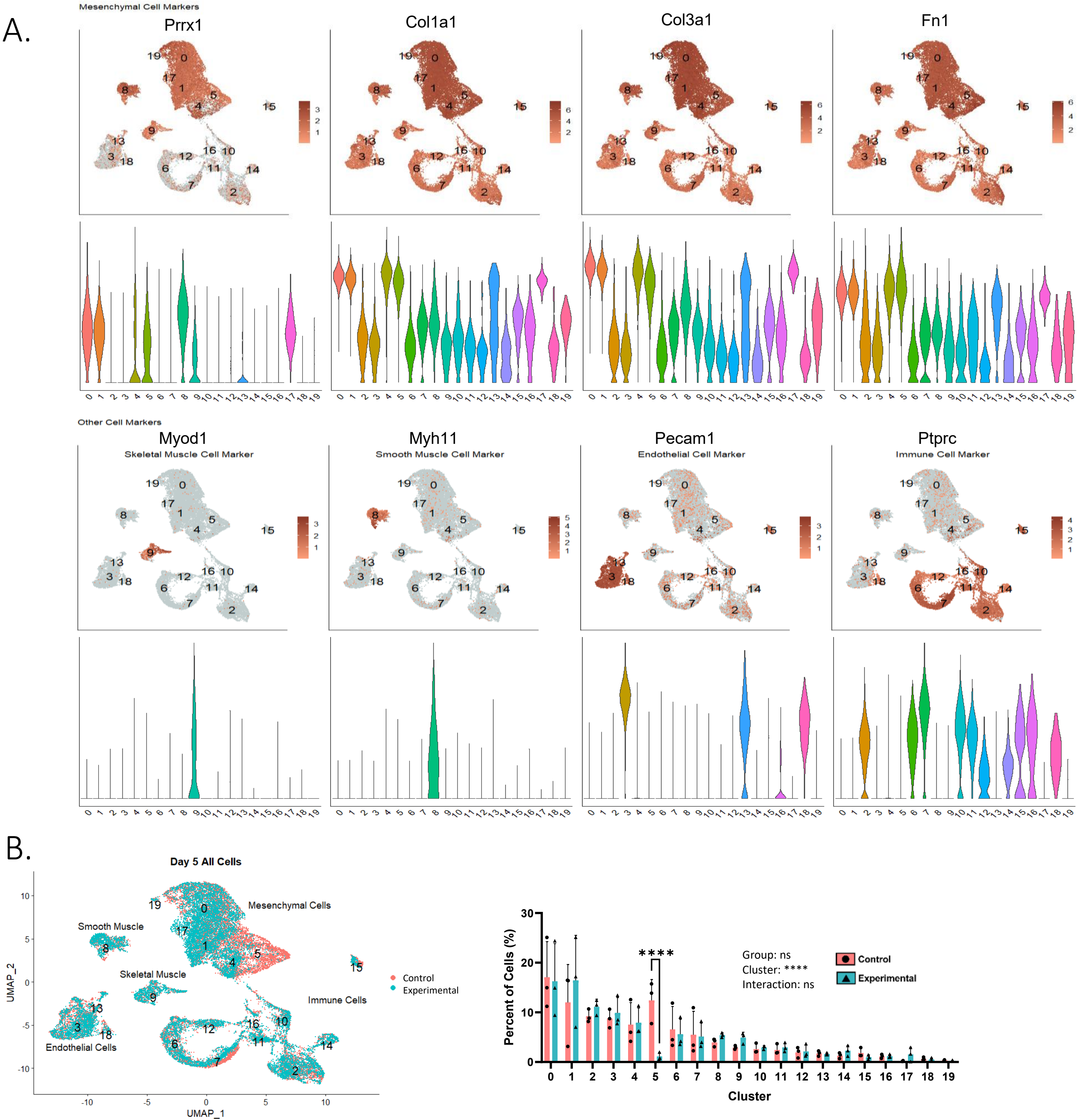

Supplemental Figure S2: Ablating proliferating 3.6Col1a1-lineage cells during the first 5 days of healing alters fracture callus mesenchymal cell and tissue composition.
(A) Expression of representative marker genes of mesenchymal cells (top row) and other cell types (bottom row) projected on UMAPs and displayed as violin plots including all callus cells. **(B)** UMAP showing cells colored by group (Control, Exptl). Percent of cells in each cluster by group was quantified (number of cells in cluster/total number cells from that callus) for each replicate callus and plotted (n=3). (Two-Way ANOVA with Holm-Sidak Post Hoc test; **** $p<0.001$)

Supplemental Figure S2. (cont.) Col1-TK mice.

C. Representative DEGs for Mesenchymal Clusters at Day 5

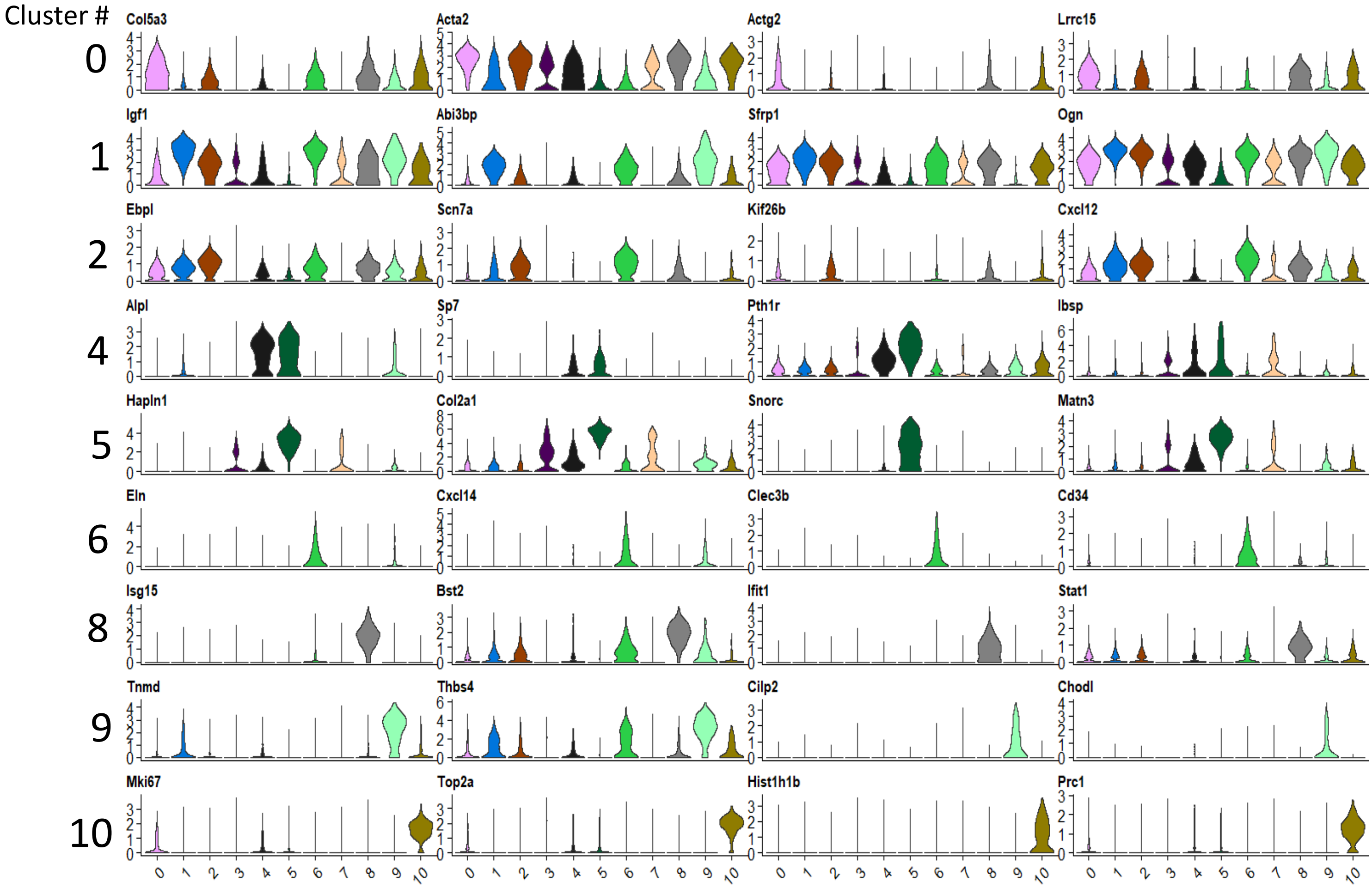

D. Extracellular Matrix Formation and Remodeling Gene Expression at Day 5

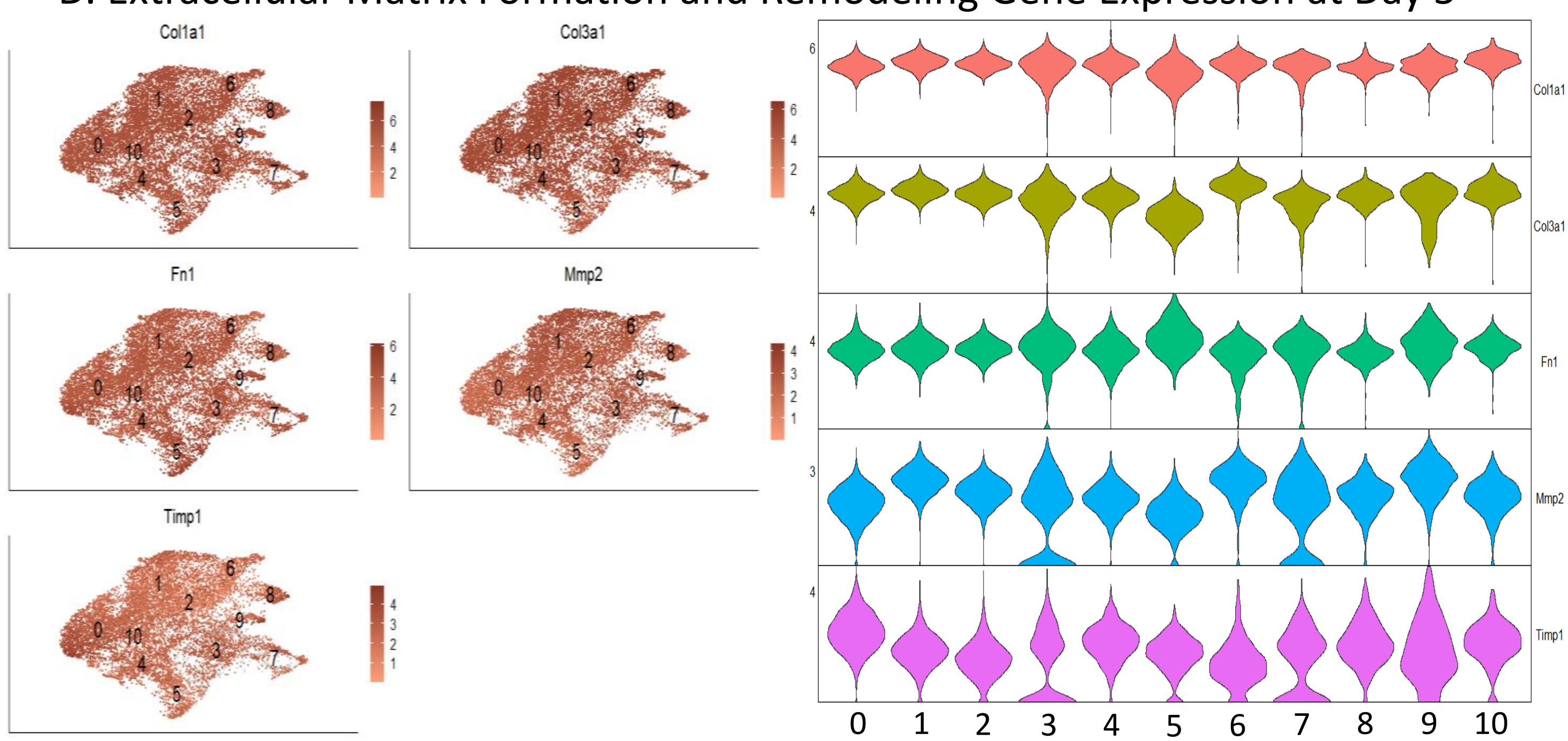

Figure S2. (C) Violin plots of four representative DEGs for each mesenchymal cluster. These and other top 50 DEGs were used to annotate cell types for each cluster. (Clusters 3 and 7 are not included because those were poorly defined.) **(D)** UMAPs and violin plots of gene expression for ECM-related genes.

Supplemental Figure S2. (cont.) Col1-TK mice

E. Cxcl12 Expression at Day 5

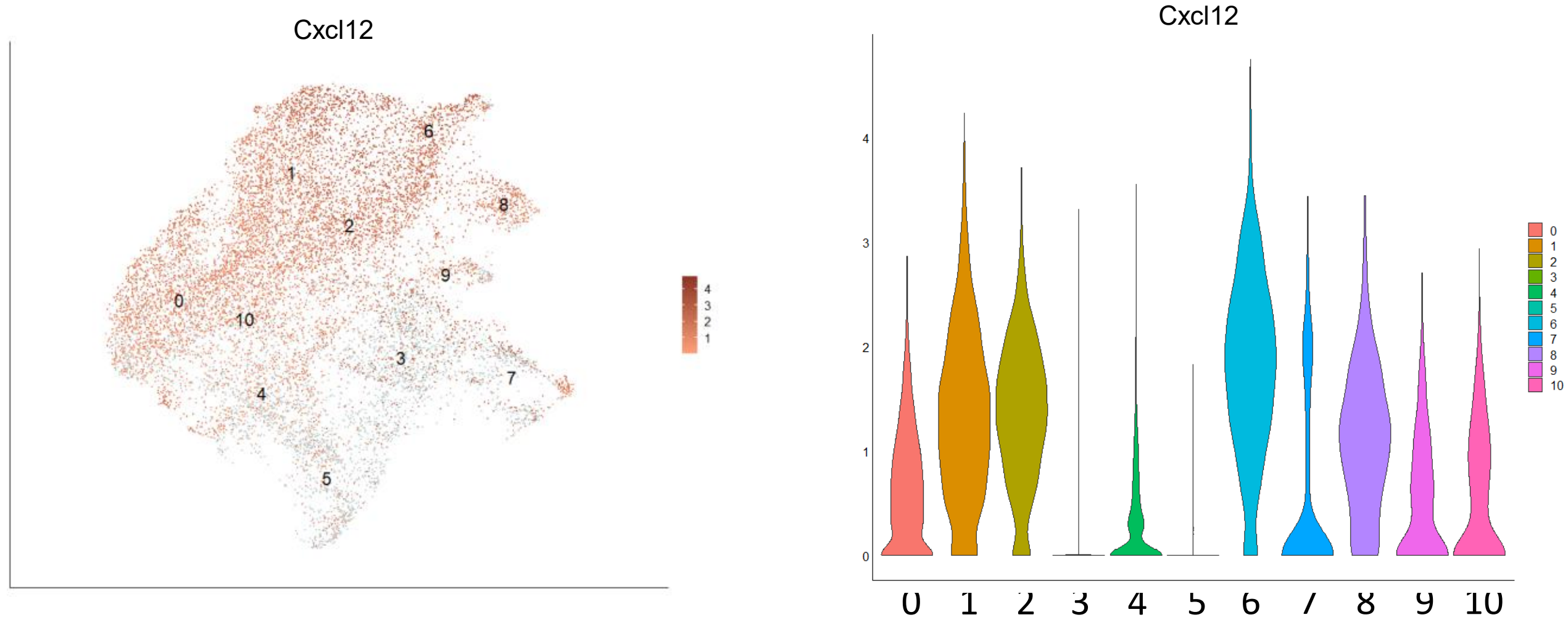

F.

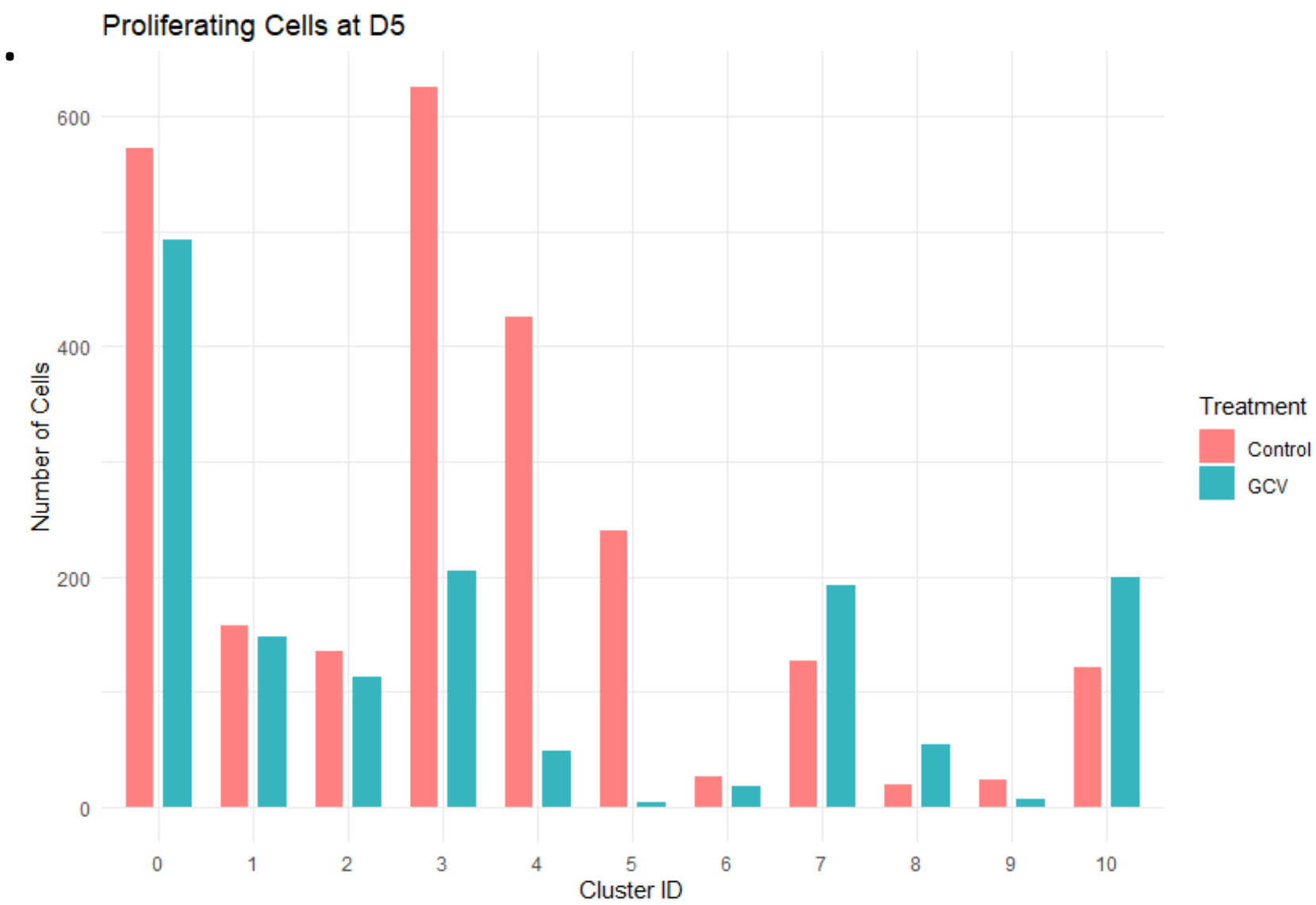

G.

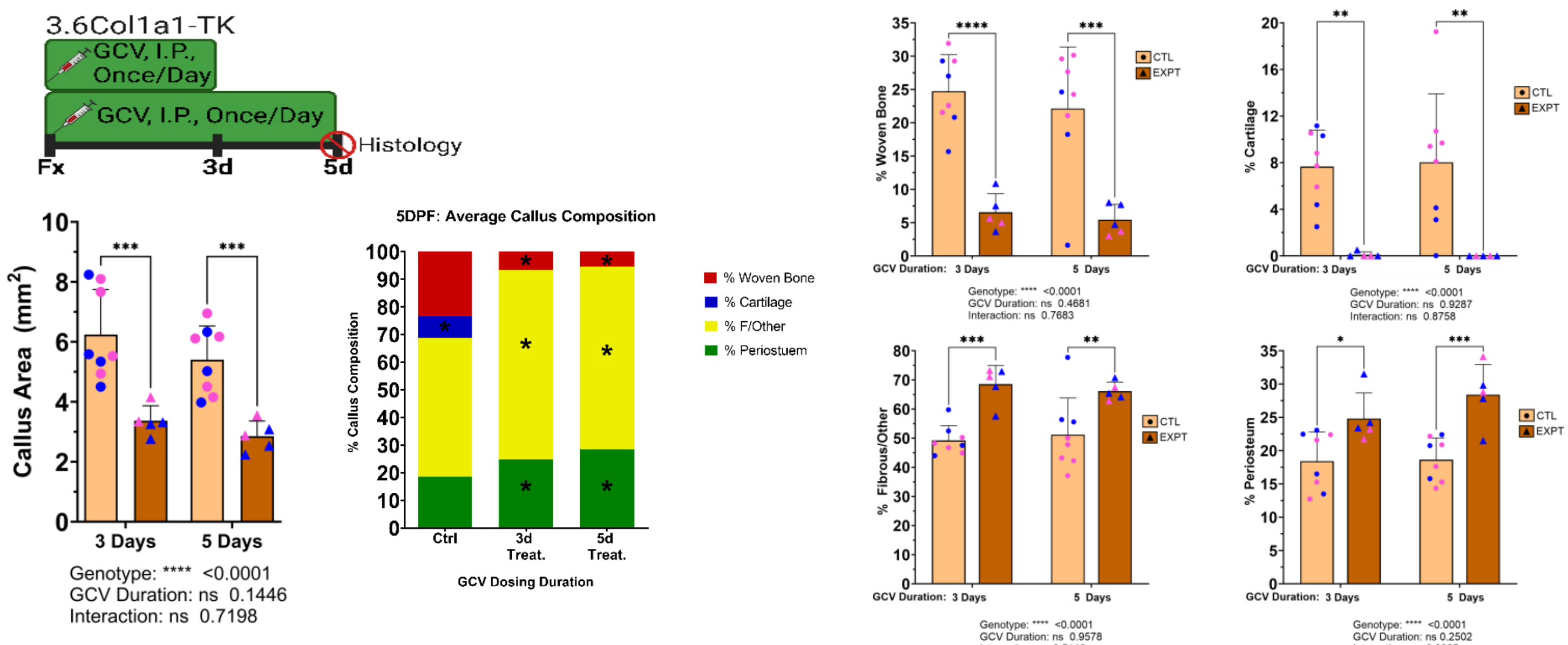

Figure S2. (E) Cxcl12 gene expression was relatively high in MPC clusters 1, 2 and 6. **(F)** The number of proliferating cells (in G2/M or S phase) shown for each cluster by treatment. **(G)** Dosing scheme for 5 DPF (top left). Histological callus area and composition at 5 DPF, breakdown by tissue type (right). Analysis performed on PSRAB stained slides as shown in Fig. S1C. (Graphs depict mean±SD; statistical differences were determined by Two-Way ANOVA with Holm-Sidak post-hoc test. *p<0.05, **<0.01, ***<0.005, ****<0.0001)

Supplemental Figure S2H (cont.) Col1-TK mice. PCNA & TK IHC at 5DPF

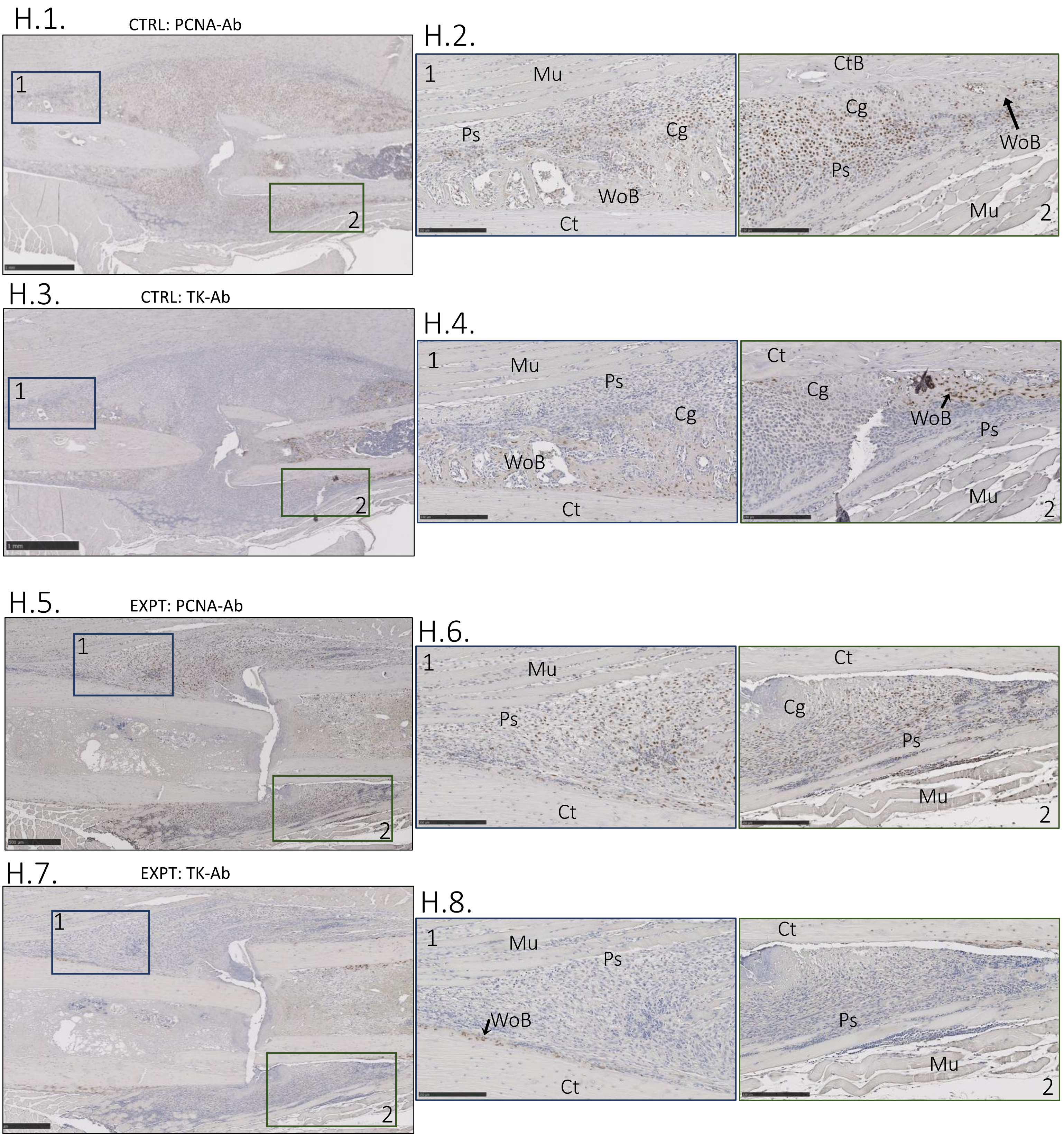

Figure S2H: Ablation of proliferating 3.6Col1a1-lineage cells significantly reduces TK+ cells without affecting overall proliferation in the fracture callus

Images of entire callus region stained by immunohistochemistry for PCNA **(H.1,H.5)** and TK **(H.3,H.7)** expression (serial sections) of representative experimental and control samples at 5 days post fracture. (scale bar = 500 µm, 1 mm) **(H.2,4,6,8)** High magnification fields from respective left side image, used for DAB quantification and showing expression at different fracture callus tissues: woven bone (WoB), cartilage (Cg), periosteum (Ps), muscle (Mu), and cortical bone (Ct). (scale bar = 250 µm).

Supplemental Figure S2I (cont.) Col1-TK mice 5-Days Post Fracture: IHC Quantification

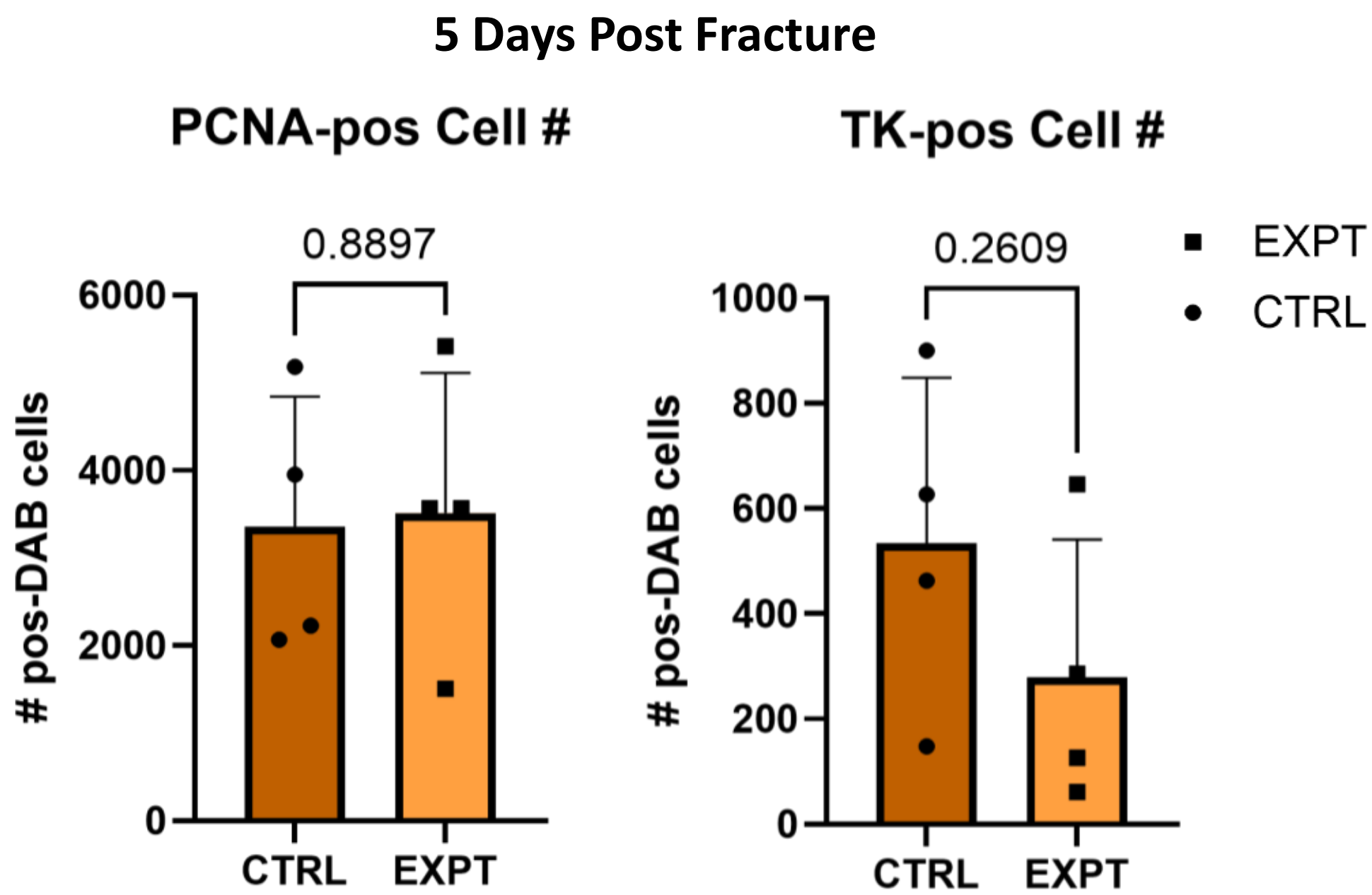

Figure S2I: Ablation of proliferating 3.6Col1a1-lineage cells reduces TK+ cells without affecting overall proliferation in the fracture callus.

Average total number of cells expressing PCNA (left) and TK (right) based on threshold for DAB-positive signal at 5 days post fracture. Similar PCNA-expression in control and experimental mice shows little impact from ablation of proliferating 3.6Col1a1-lineage cells. Trending, but not significant, lower numbers of TK-positive cells found in experimental mice compared to control mice. (Graphs depict mean±SD; statistical differences were determined by Welch's t test)

Supplemental Figure S3. Col1-TK Day 10 (This figure supplements main Figure 3.)

A.

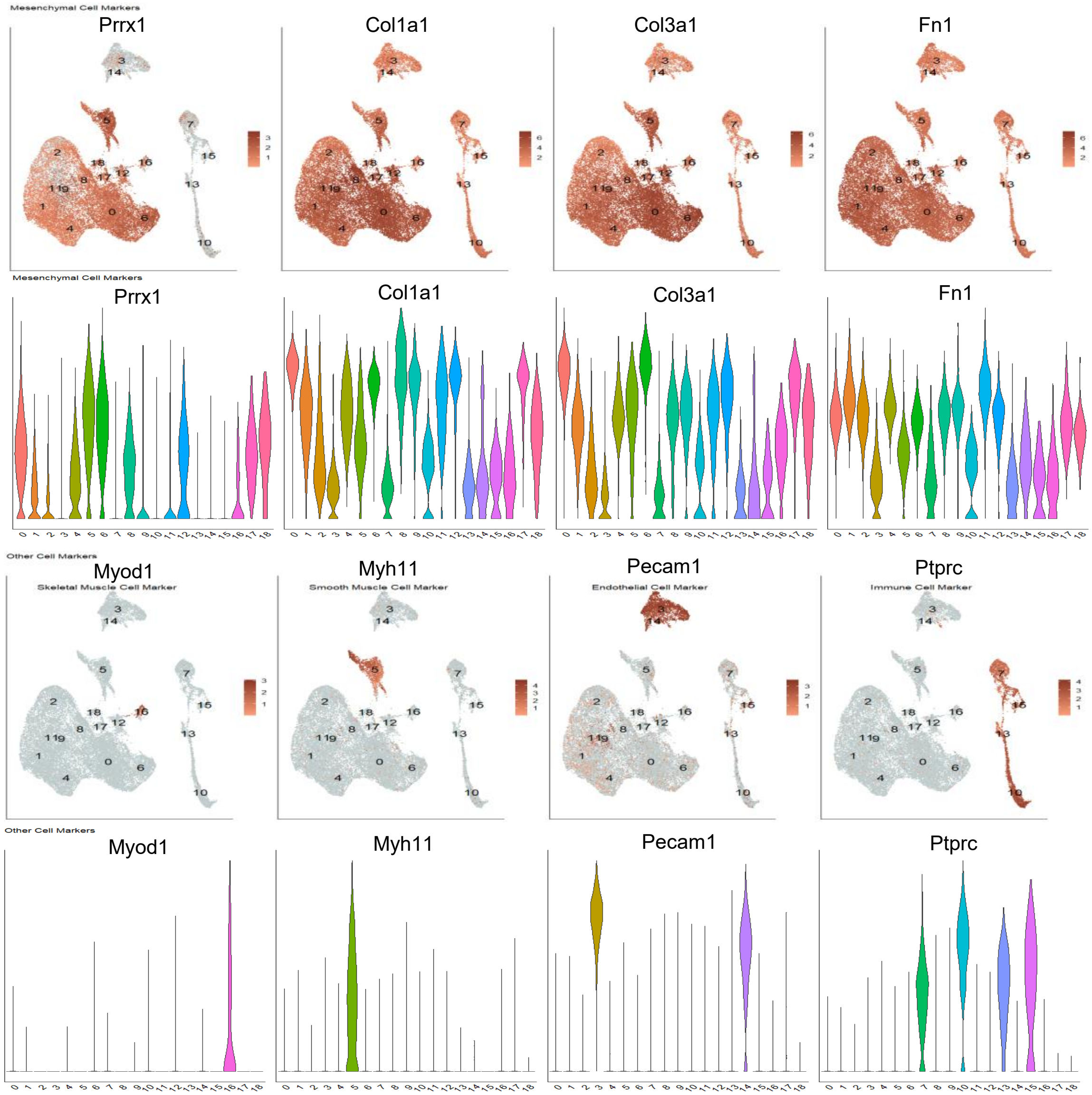

B.

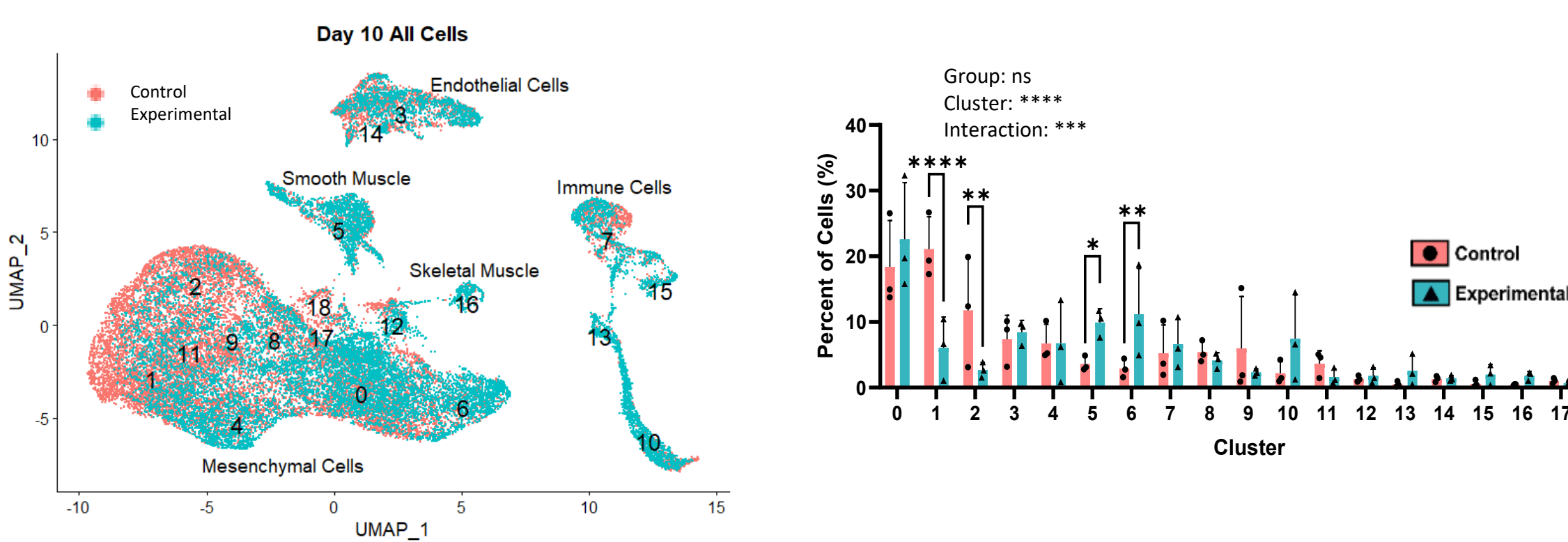

Supplemental Figure S3: Ablating proliferating 3.6Col1a1-expressing cells during the first 10 days of healing significantly alters fracture callus mesenchymal cell and tissue composition.

(A) Expression of representative marker genes for mesenchymal cells (top row) and other cell types (bottom row) projected on UMAPs and displayed as violin plots including all callus cells. **(B)** UMAP showing cells colored by group (Control, Exptl). Percent of cells in each cluster by group was quantified (number of cells in cluster/total number cells from that callus) for each replicate callus and plotted (n=3). (Two-Way ANOVA with Holm-Sidak Post Hoc test; *p<0.05, **<0.01, ***<0.005, ****<0.0001)

Supplemental Figure S3. (cont) Col1-TK mice

C. Canonical Marker Genes for Mesenchymal Clusters at Day 10

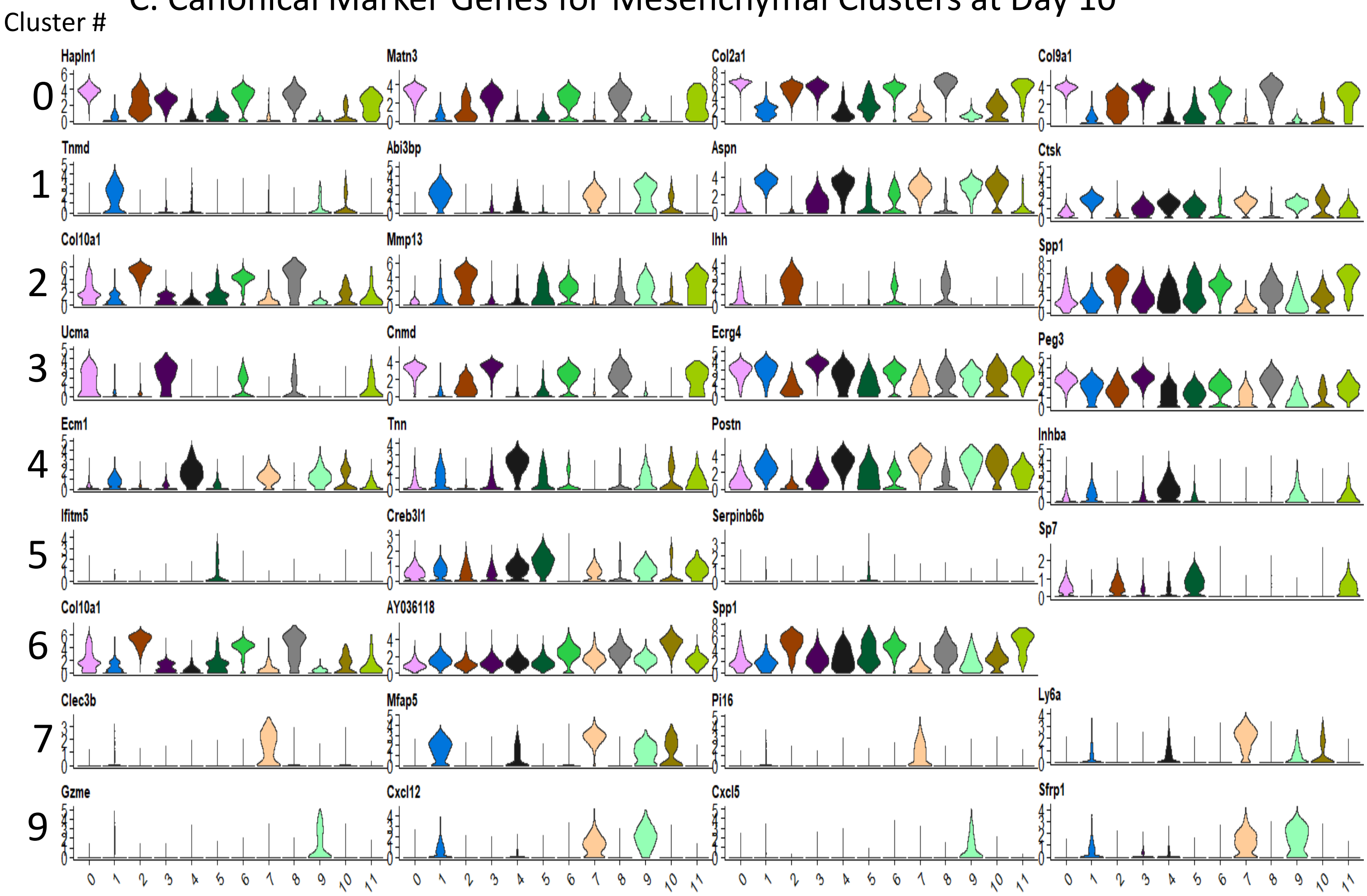

D.

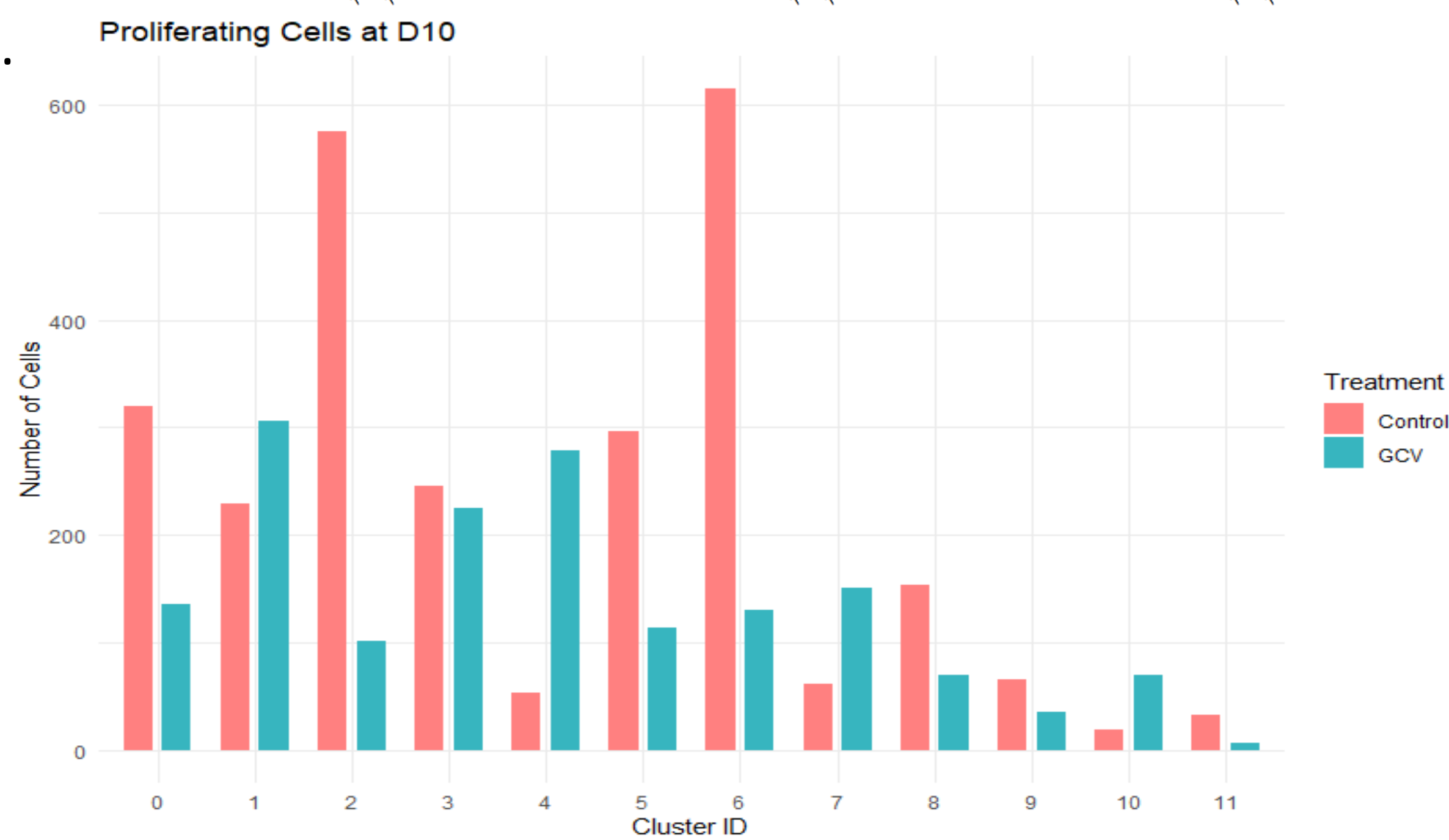

Figure S3. (C) Violin plots of four representative DEGs for each mesenchymal cluster. These and other top 50 DEGs were used to annotate cell types for each cluster. (Clusters 8, 10 and 11 are not included because those were poorly defined.) **(D)** The number of proliferating cells (in G2/M or S phase) shown for each cluster by treatment.

Supplemental Figure S3. (cont.) Col1-TK Day 10.

E.

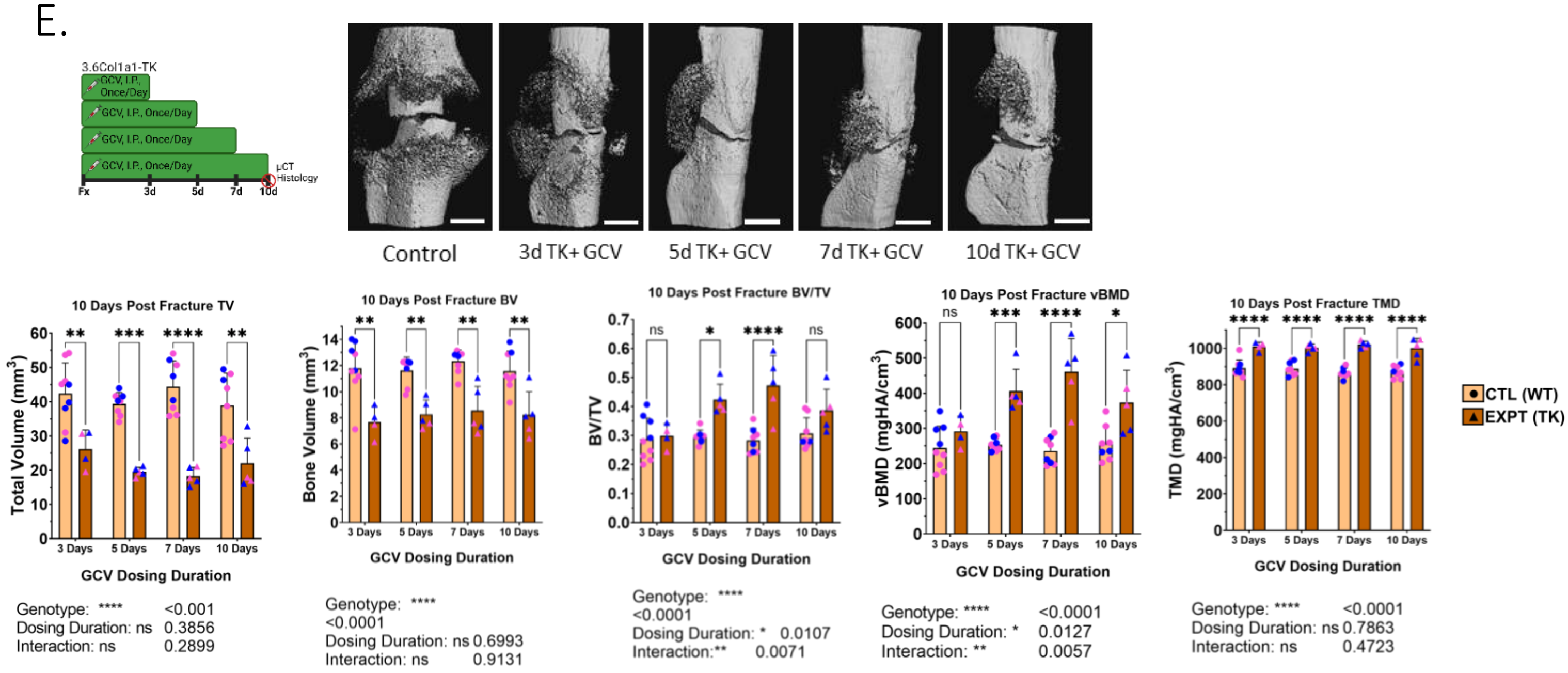

F.

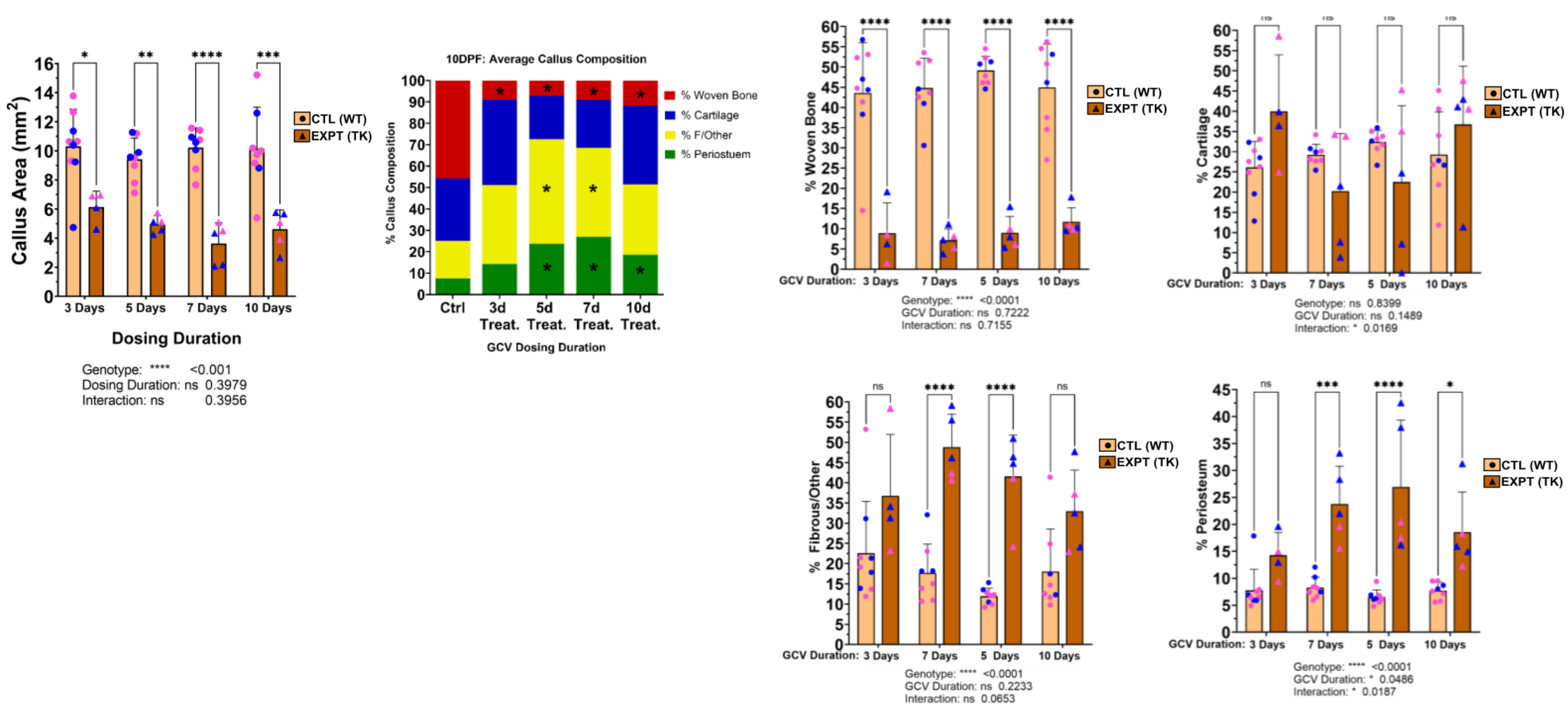

Figure S3. (E) Dosing scheme for 10 DPF (top left), microCT representative reconstructions (top right) and graphs of parameters (bottom) analyzed from the callus region at 10 DPF (includes callus + cortical bone). **(F)** Histological analysis of callus area and composition at 10 DPF determined from sections stained by PSRAB and breakdown by tissue type. (Graphs depict mean ± SD; statistical differences in E determined by Two-Way ANOVA with Holm-Sidak Post-Hoc test, and in F by Fisher's Exact Test)

Supplemental Figure S3 (cont.) Col1-TK mice. PCNA & TK IHC at 10 DPF

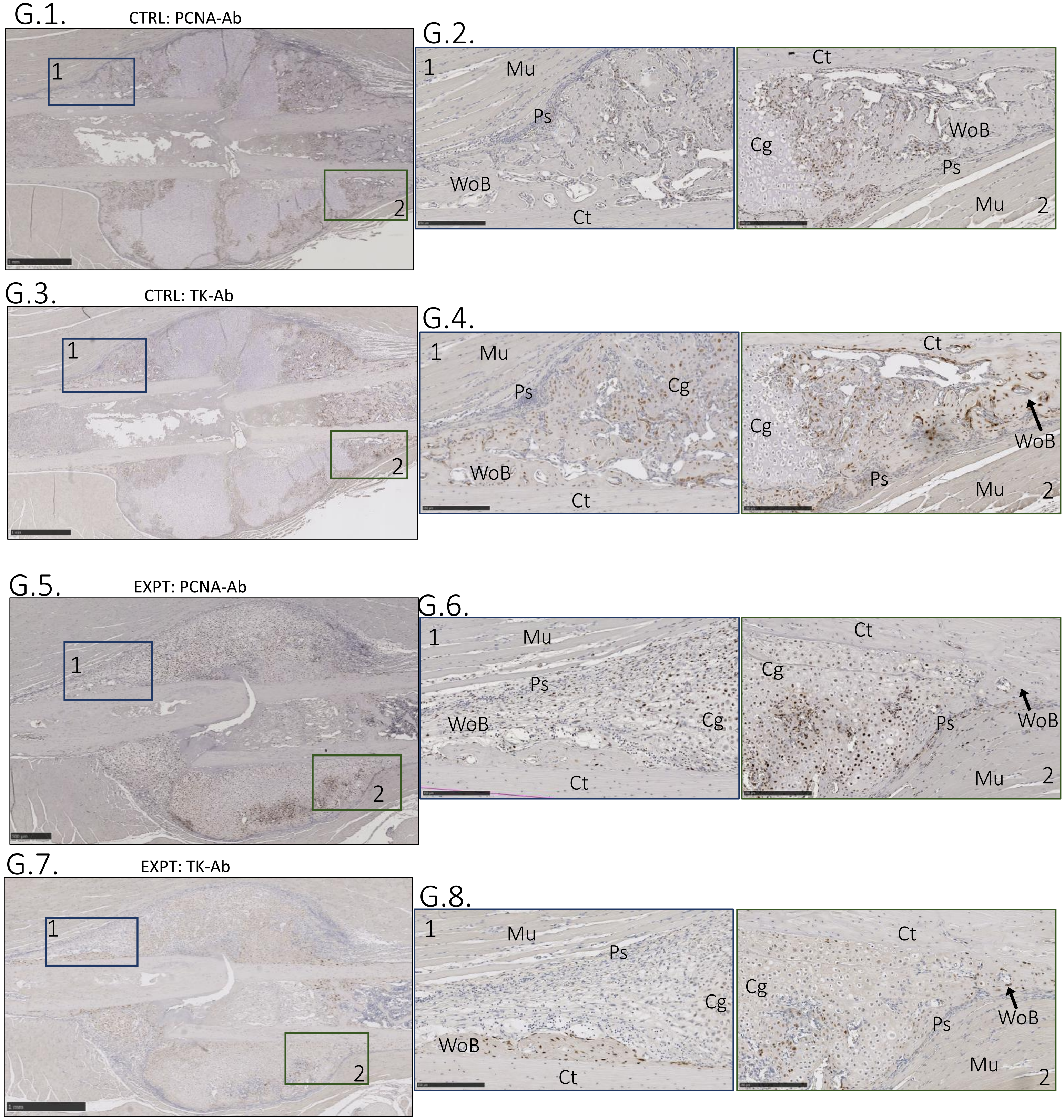

Supplemental Figure S3G: Ablation of proliferating 3.6Col1a1-lineage cells significantly reduces TK+ cells without affecting overall proliferation in the fracture callus at 10 DPF.
Representative images of immunohistochemistry for PCNA **(G.1,G.5)** and TK **(G.3,G.7)** expression in an experimental and control sample, respectively, at 10 days post fracture. (scale bar = 500 µm, 1 mm) **(G.2,4,6,8)** High magnification fields from respective left side image, used for DAB quantification and showing fracture callus tissues: woven bone (WoB), cartilage (Cg), periosteum (Ps), muscle (Mu), and cortical bone (Ct). (scale bar = 250 µm).

Supplemental Figure S3H. Col1-TK mice, 10-Days Post Fracture: IHC Quantification

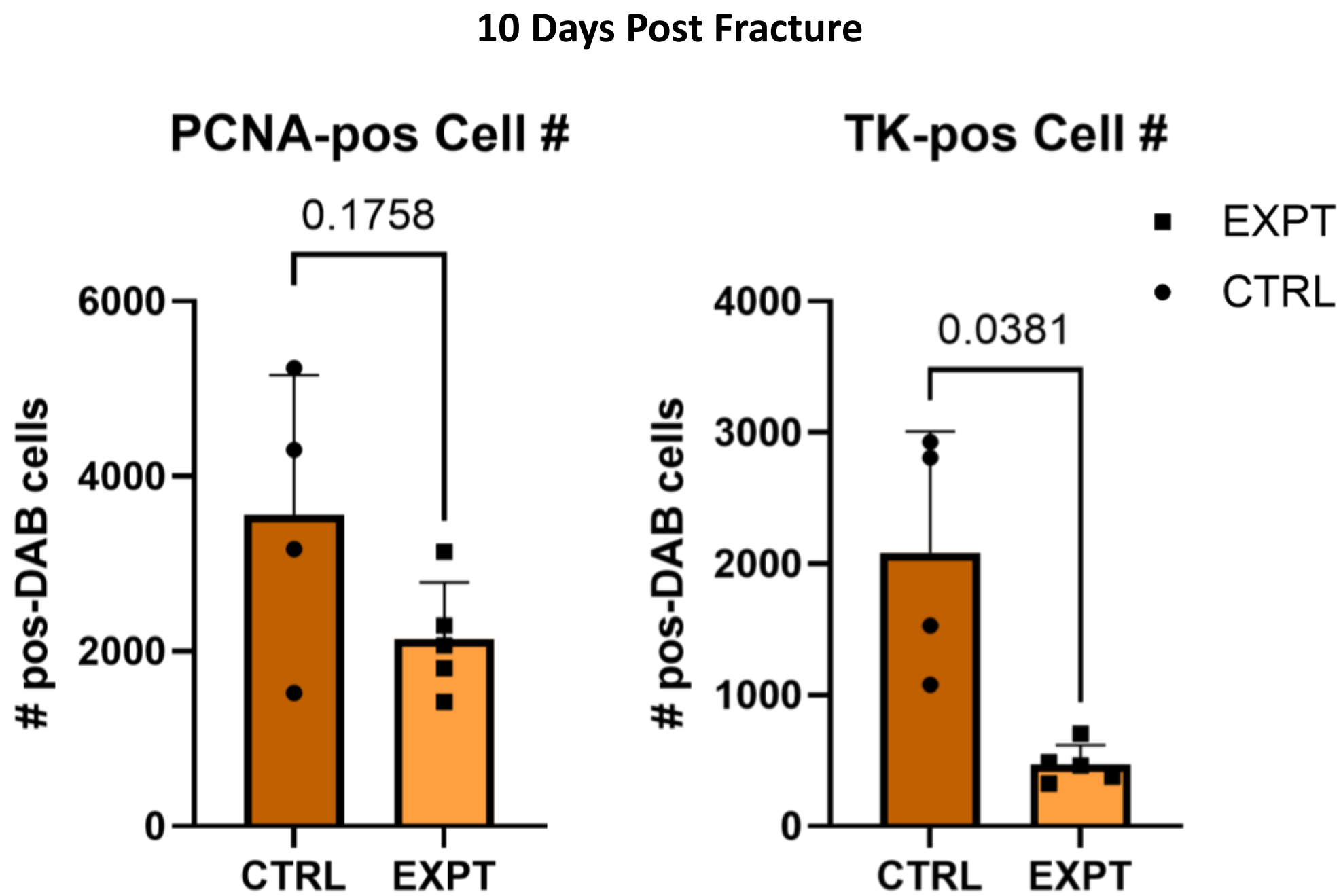

Supplemental Figure 3H: Ablation of proliferating 3.6Col1a1-lineage cells significantly reduces TK+ cells without affecting overall proliferation in the fracture callus.
Average total number of cells expressing PCNA (left) and TK (right) based on threshold for DAB-positive signal at 10 days post fracture. A non-significant trend of fewer PCNA-expressing cells observed in experimental mice. Significantly fewer TK-positive cells found in experimental mice compared to control mice following ablation of 3.6Col1a1-lineage cells. (Graphs depict mean±SD; statistical differences were determined by Welch's t test)

Supplemental Figure S3. (cont) Col1-TK mice

I. Extracellular Matrix Formation and Remodeling Gene Expression at Day 10

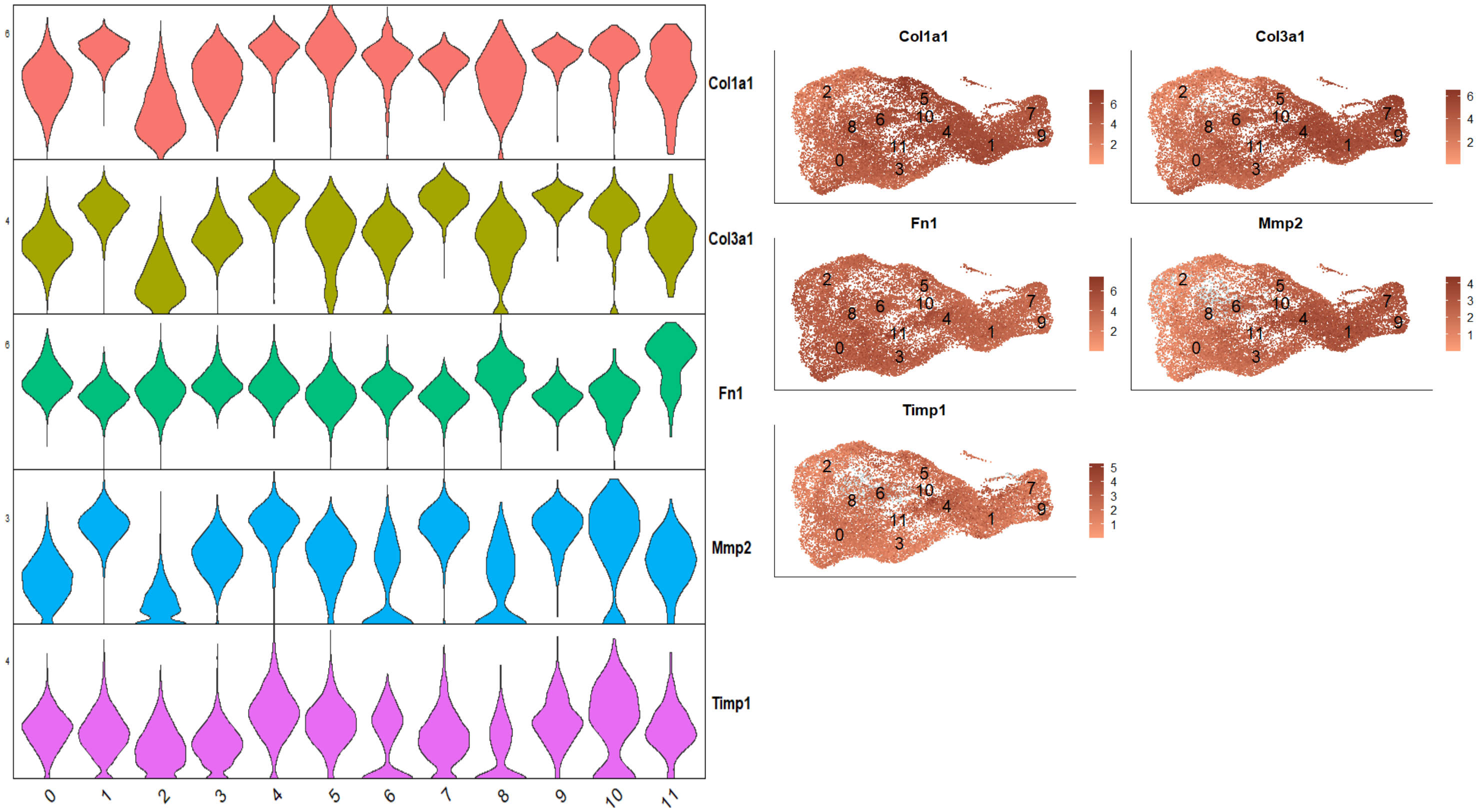

Figure S3. (I) UMAPs and violin plots of gene expression for ECM-related genes.

Supplemental Figure S3. (cont) Col1-TK mice.

J. Chondrogenic Gene Expression at Day 10

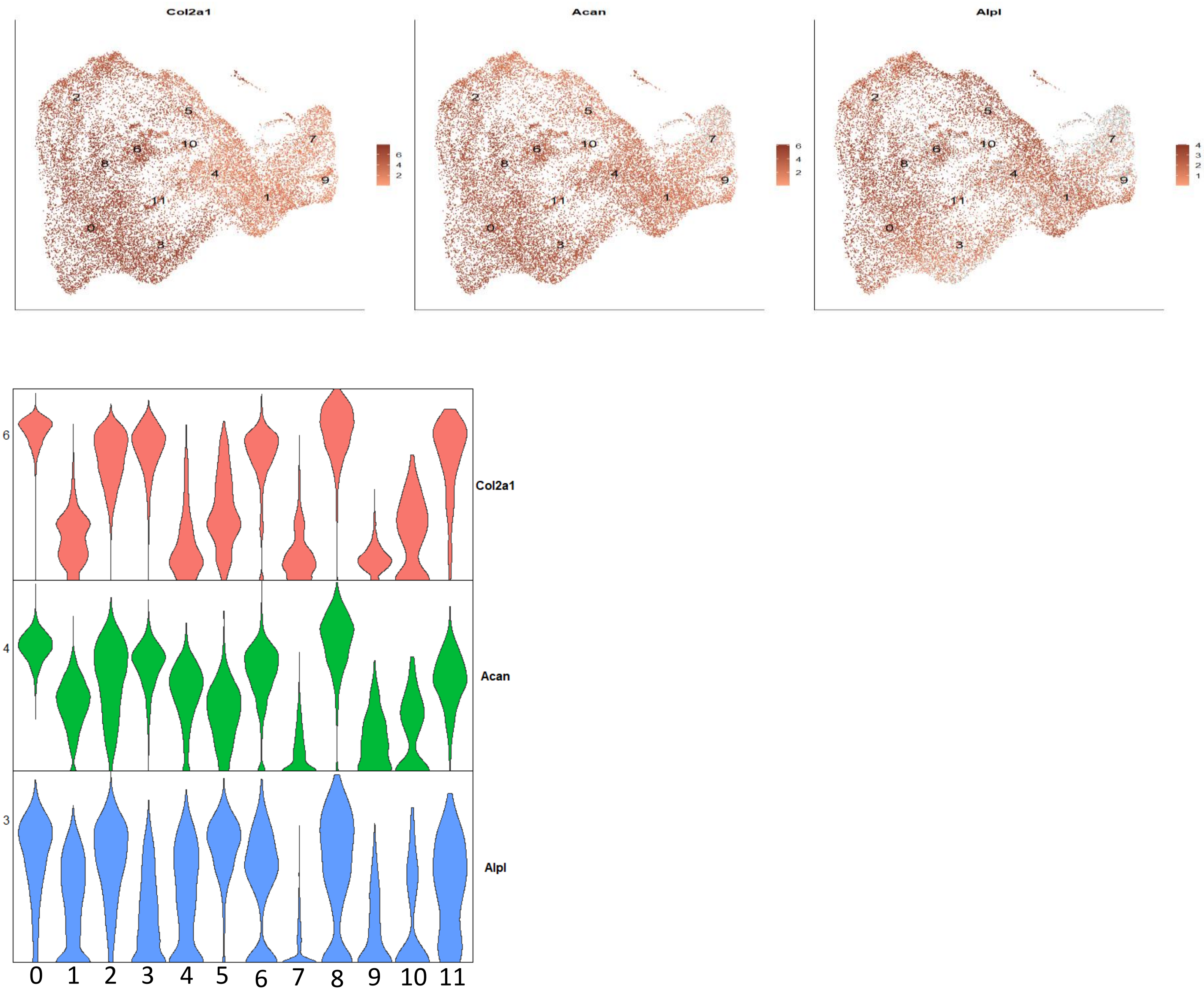

Supplemental Figure S3. (J) UMAPs and violin plots of gene expression showing broad expression of chondrocyte-related genes on day 10.

Supplemental Figure S4

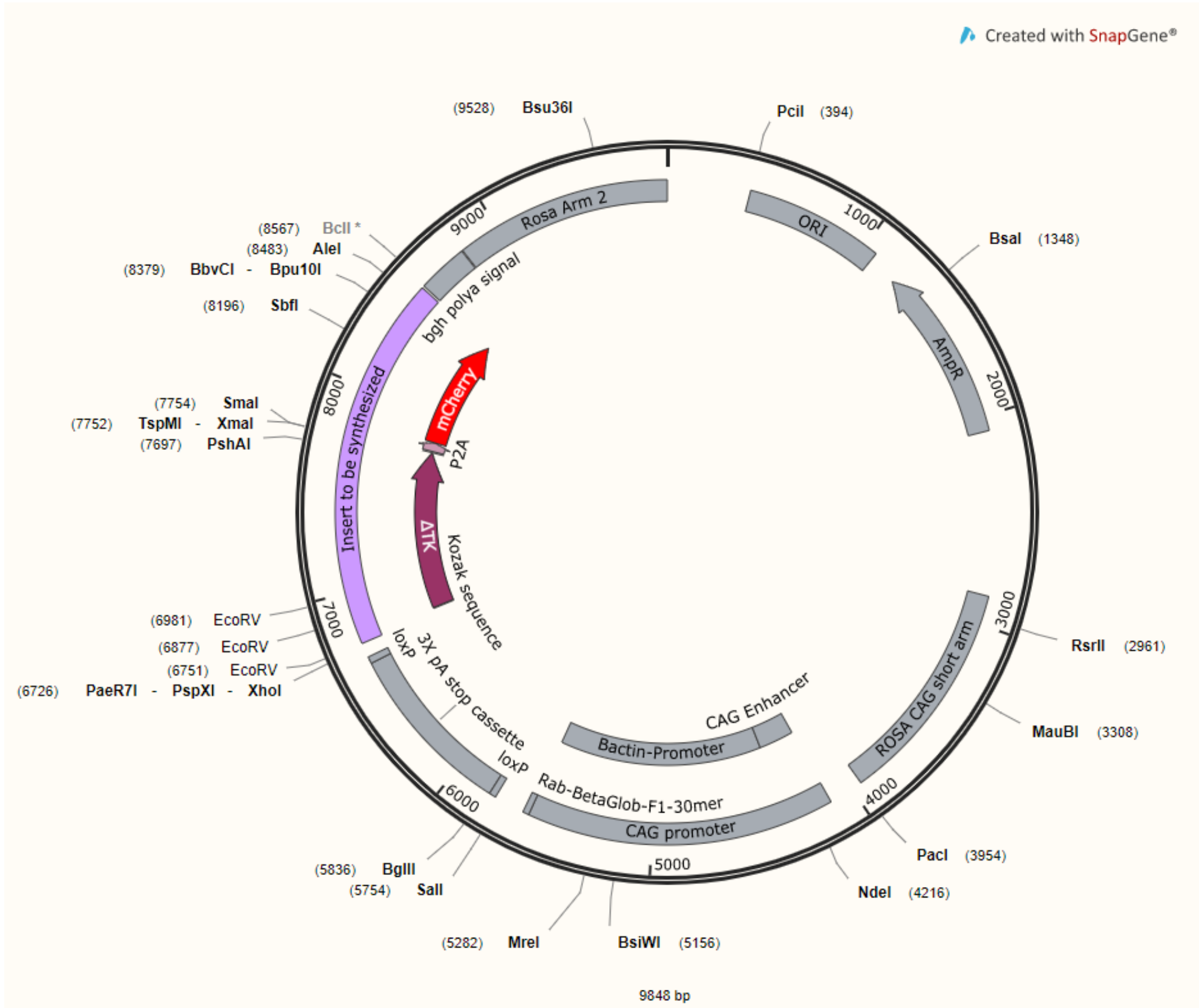

Supplemental Figure S4: Targeting plasmid used to create ROSA-TK founders.
Targeting plasmid that contains a CAG promoter, floxed stop cassette, the HSV-deltaTK allele, a P2A self-cleaving site, and an mCherry allele.

Supplemental Figure S5

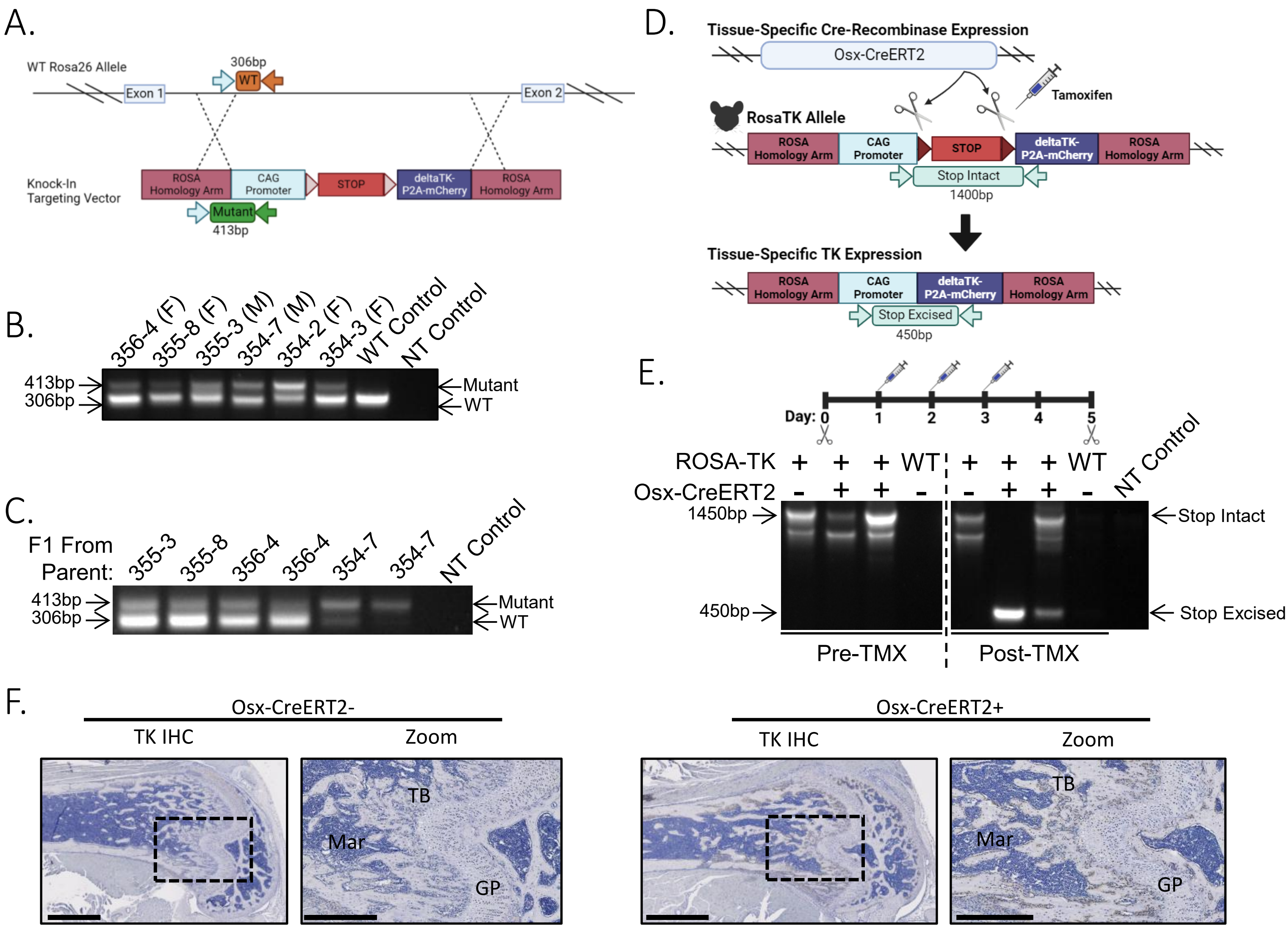

Supplemental Figure S5: Generation of a novel ROSA-TK mouse line to drive tissue-specific TK expression using Cre-recombinase.
(A) Schema of plasmid used to recombine the targeting vector into the ROSA26 allele. Genotyping primers to confirm proper recombination utilized a forward primer targeting the ROSA26 homology arm and reverse primers targeting either the wild-type ROSA26 allele or the mutant CAG promoter. **(B)** Six heterozygous founders were identified using the above-described primers and were then bred with wild-type B6 mice. **(C)** Progeny from each of the six founders maintained the mutant allele. **(D)** Genotyping scheme to evaluate stop cassette excision in response to tamoxifen (TMX) activation of inducible Osx-CreERT2. Genotyping primers spanning either the intact stop cassette or the CAG promoter and TK gene delineate the intact versus excised stop cassette. **(E)** Tail snips from Osx-CreERT2;ROSA-TK mice prior to TMX treatment shows only intact stop cassettes. After three TMX treatments, a second tail snip from the same mice shows stop cassette excision only in mice that have both ROSA-TK and Osx-CreERT2 alleles. Mice lacking Osx-CreERT2 show retention of the stop cassette and mice without the ROSA-TK allele show no PCR amplification of either the intact or excised stop cassette products. **(F)** IHC for TK shows that TMX induces TK expression in osteoblasts lining the trabecular bone in the growth plate in 5-week-old Osx-CreERT2;ROSA-TK mice but not Osx-CreERT2-(neg); ROSA-TK mice. Scale bar = 1 mm in TK IHC, 0.5 mm in Zoom.

Supplemental Figure S6. OsxCreERT2; ROSA-TK mice. (Supplement to manuscript Figure 4.)

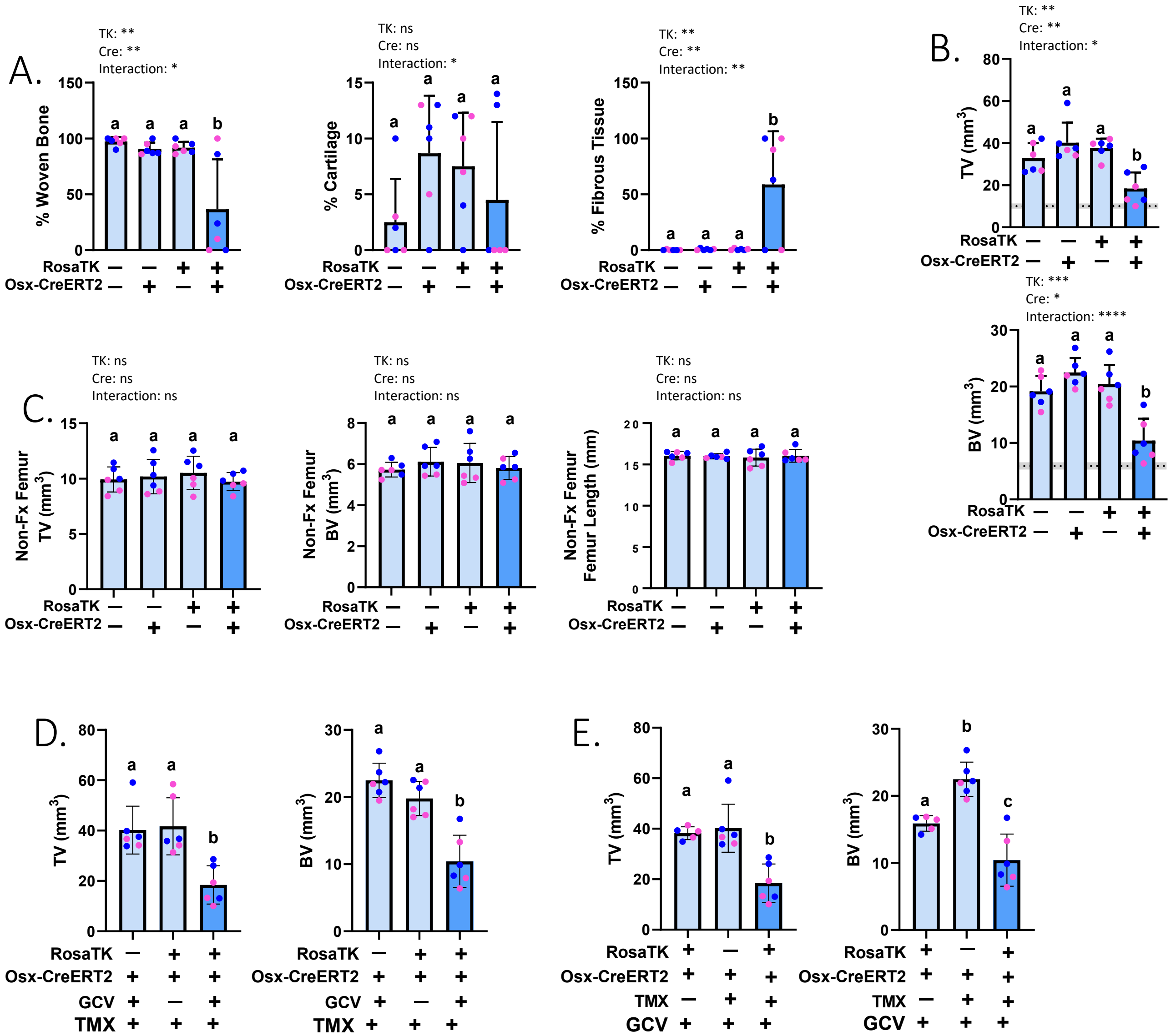

Supplemental Figure S6: Ablation of proliferating Osterix-expressing cells significantly impairs fracture callus mineralization without affecting the contralateral non-fractured limb.
(A) Histological analysis of callus. Ablation of proliferating Osterix-expressing cells for 14 days post-fracture significantly reduces the amount of woven bone and significantly increases the amount of fibrous tissue within the fracture callus, without affecting %cartilage. These data are summarized in the stacked bar graph in Figure 4D. **(B)** microCT analysis of the callus region (includes callus + cortical bone). Ablation of proliferating Osterix-expressing cells for 14 days post-fracture significantly reduces Total Volume (TV) and Bone Volume (BV) at the site of fracture callus. (Dashed lines indicate avg. value of contralateral intact femurs; shading denotes +/- SD). **(C)** microCT analysis of non-fractured bones. Treatment with GCV does not significantly change TV, BV, or femur length in the contralateral, non-fractured femurs. (All mice in A-C were dosed with GCV.) **(D)** Impairments in fracture callus size and bone volume are only seen in mice that are Cre+;TK+ and treated with GCV, even if all received TMX treatment. **(E)** Impairments in fracture callus size and bone volume are only seen in mice that are Cre+;TK+ and are treated with TMX, even if all received GCV treatment. (The third bar of graphs in panels D and E show identical data, also shown in the fourth bar of panel B.) (Graphs depict mean±SD and statistical differences were determined by (A-C) a Two-Way ANOVA with Holm-Sidak Post-Hoc test or (D & E) Kruskal-Wallis test.)

Supplemental Figure S7. Co-Immunofluorescence. (Supplement to manuscript Figure 5.)

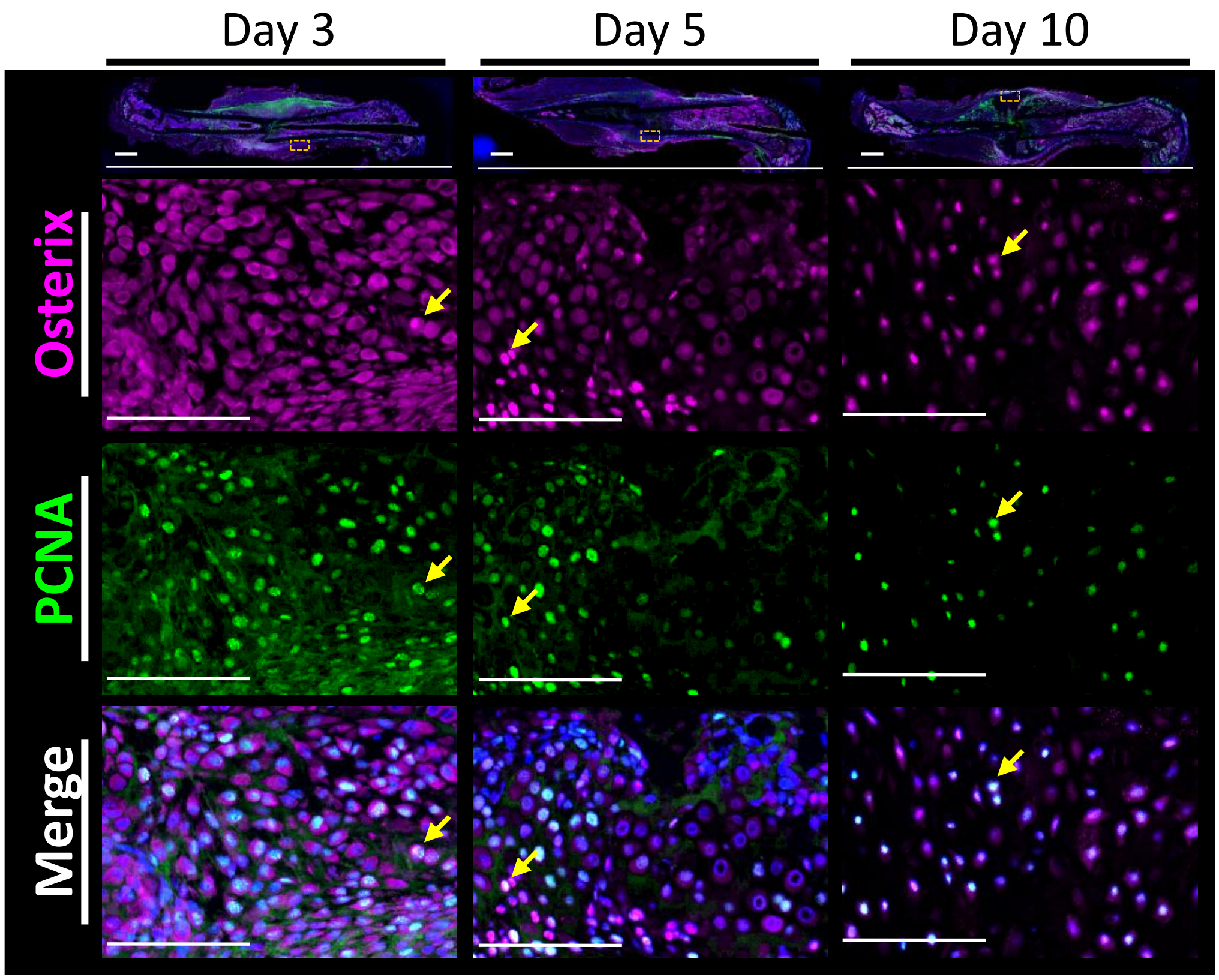

Supplemental Figure S7: Sp7/Osterix-expressing cells in cartilage regions proliferate during fracture healing.

Representative fracture callus sections from Control mice at 3, 5 and 10 days post-fracture stained by immunofluorescence for Osterix and proliferation marker PCNA. Top row: low-power images showing entire femur. Bottom rows: higher power images of chondrocyte/cartilage region of callus (region outlined by dashed yellow box above). (Scale bars: Low-Power=1 mm, High-power=0.1 mm; Yellow Arrows=Osx+;PCNA+ cells.)

Supplemental Figure S8. Ocn-Cre;ROSA-TK mice – 14 DPF. (Supplement to manuscript Figure 6.)

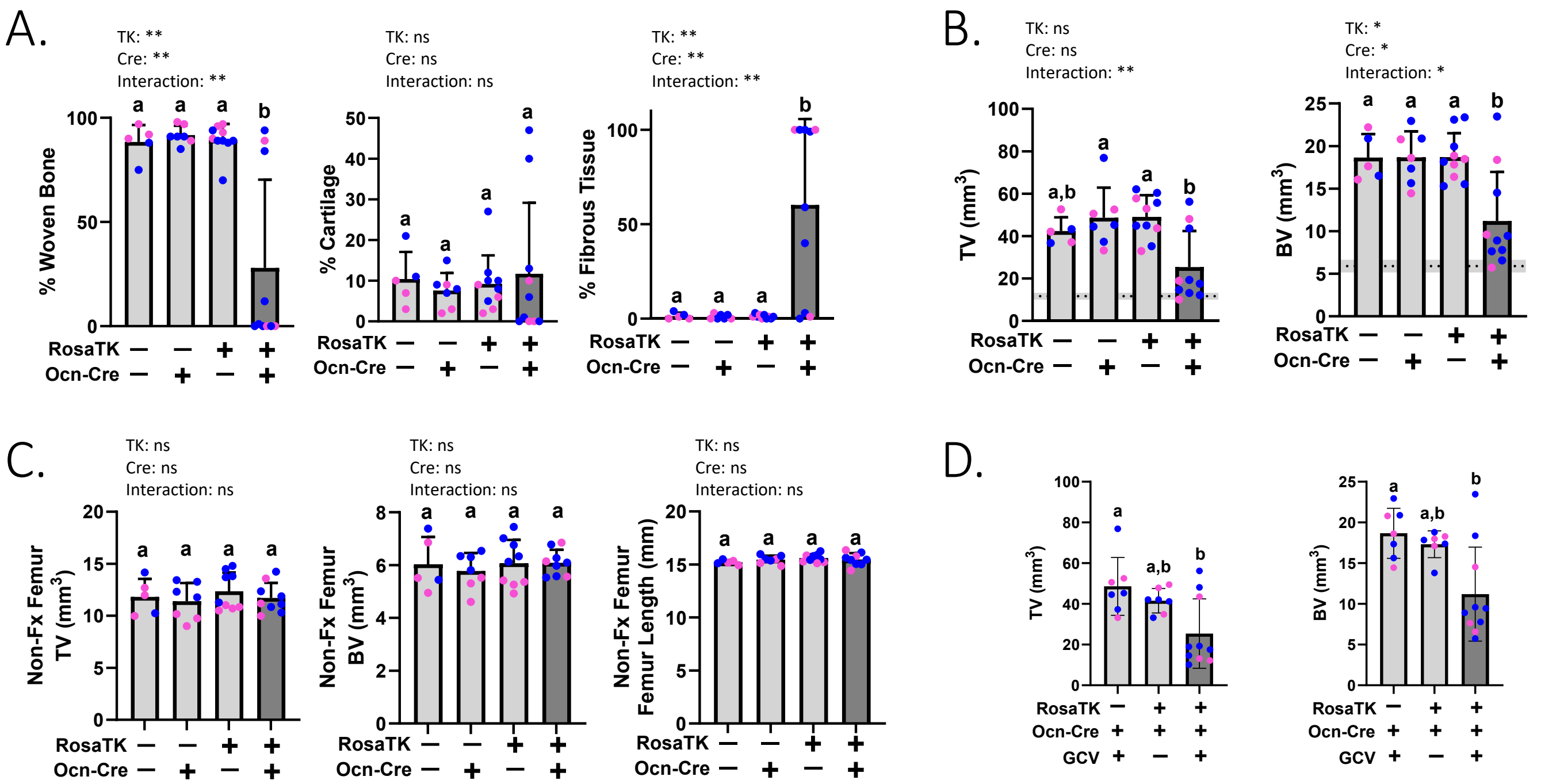

Supplemental Figure S8: Ablation of proliferating Osteocalcin-lineage cells significantly impairs fracture callus mineralization without affecting the contralateral non-fractured limb.
(A) Fracture callus composition was determined from histology. TK+/Ocn-Cre+ mice have reduced woven bone and increased fibrous tissue within the fracture callus without affecting cartilage composition. These data are summarized in the stacked bar graph in Figure 6D. **(B)** MicroCT analysis of callus region (includes callus + cortical bone). TK+/OcnCre+ mice have reduced Total Volume (TV) and Bone Volume (BV). (Dashed lines indicate avg. value of contralateral intact femurs; shading denotes +/- SD). **(C)** MicroCT of intact femurs. Genotype does not alter TV, BV, or Femur Length in the contralateral, non-fractured femurs from the same cohort of mice. (All mice in A-C were dosed with GCV.) **(D)** Impairments in fracture callus morphology are only seen in mice that are Cre+;TK+ and treated with GCV. (Graphs depict mean±SD and statistical differences were determined by a two-Way ANOVA with Holm-Sidak Post-Hoc test.)

Supplemental Figure S8E. PSRAB and TK IHC staining at 14DPF

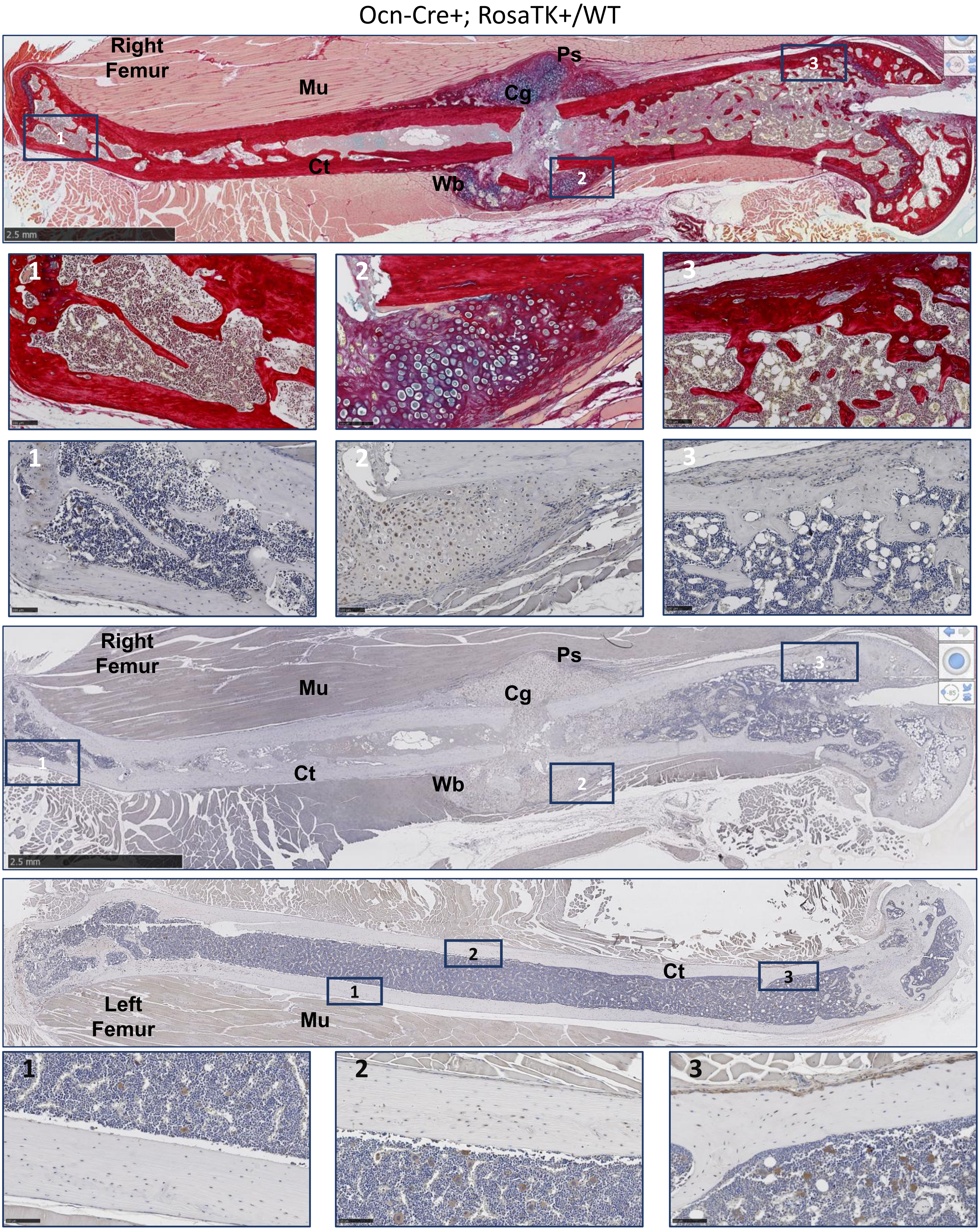

Figure S8E. Representative full bone and high magnification fields of Picrosirius Red and Alcian Blue (PSRAB) and TK-IHC stained slides for an experimental sample expressing Ocn-Cre;RosaTK alleles. Bottom whole bone image and high magnification fields show intact left femur of same sample. Staining shows no off-target effect from GCV administration, with normal morphology along distal and proximal femur and near fracture callus. TK-positive signal illustrates effectiveness of model in which only cells both actively proliferating and expressing TK gene were ablated. (Cg: cartilage, Wb: woven bone, F: fibrous tissue, Ps: periosteum, Mu: muscle)

Supplemental Figure S9. Ocn-Cre;ROSA-TK mice – Additional timepoints.
(Supplement to manuscript Figure 6.)

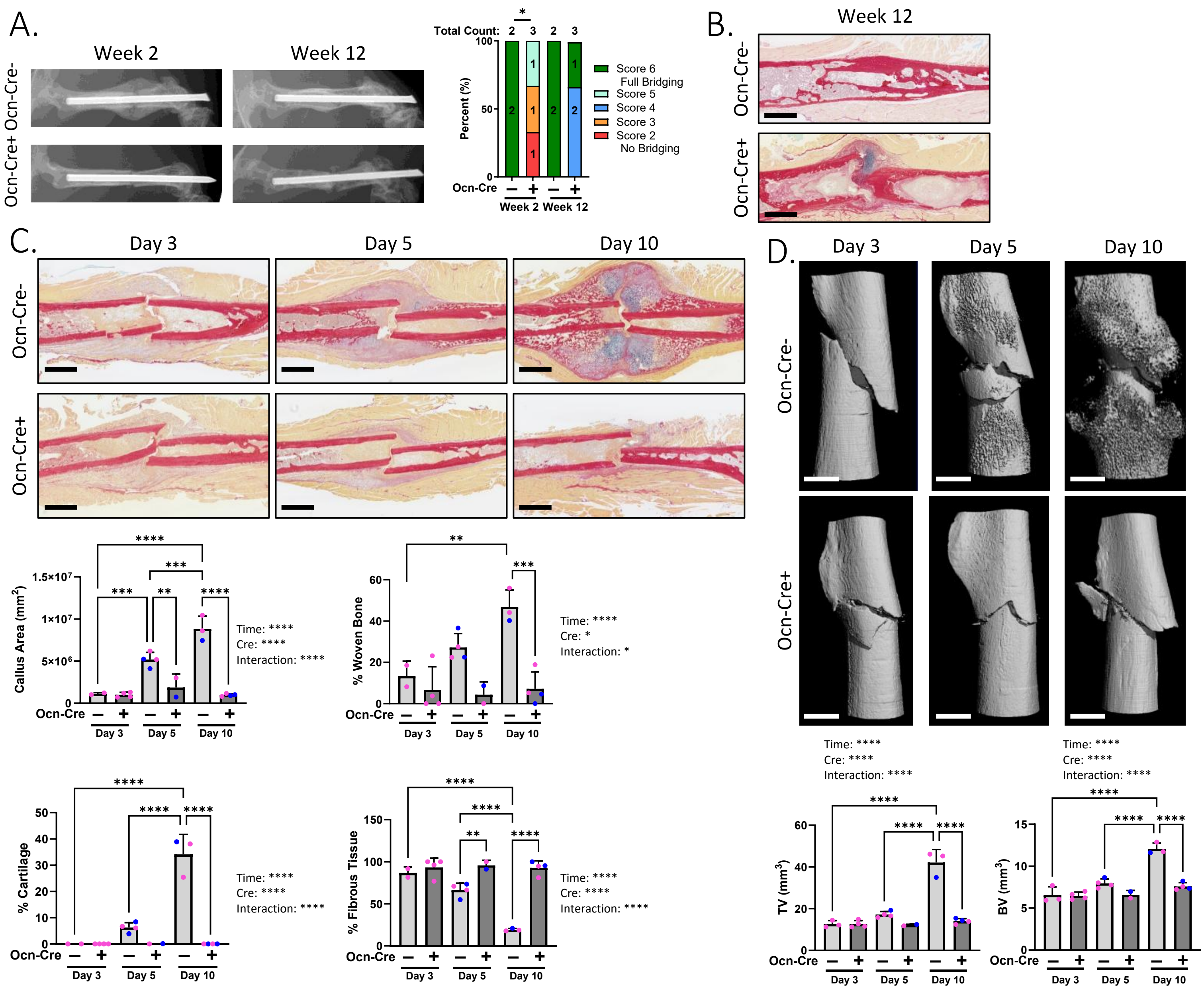

Supplemental Figure S9: Ablation of proliferating Osteocalcin-lineage cells significantly impairs fracture callus healing in long- and short-term.
(A) Ocn-Cre;ROSA-TK mice were treated with GCV for 2 weeks post-fracture (as depicted in Fig 6A), then GCV was withdrawn for the following 10 weeks. X-ray scoring shows that Ocn-Cre- mice fully bridge and remodel by 12 weeks post-fracture, whereas Ocn-Cre+ mice have significantly impaired bridging at 2 weeks post-fracture that does not fully recover by 12 weeks. **(B)** PSRAB staining of representative samples shows that Ocn-Cre- mice 12 weeks post-fracture have bony healing at the fracture site, whereas Ocn-Cre+ mice still have cartilaginous and fibrotic calluses 12 weeks post-fracture. **(C)** Ocn-Cre;ROSA-TK mice were treated with GCV for 3-, 5-, or 10-days post-fracture and euthanized on the last day of treatment indicated. PSRAB staining and analysis shows early changes in callus composition. **(D)** microCT analysis of Ocn-Cre;RosaTK mice treated with GCV for 3-, 5-, or 10-days post-fracture and euthanized on the last day of treatment indicated shows that control Cre- mice increase callus mineralization over time, whereas Cre+ experimental mice do not create a mineralized callus. (Graphs depict mean±SD; statistical differences were determined by (A) Chi-Square test, or (C-D) two-Way ANOVA with Holm-Sidak Post-Hoc test.)

Supplemental Figure S10. Osteocalcin & PCNA Immunofluoresence.
(Supplement to manuscript Figure 7.)

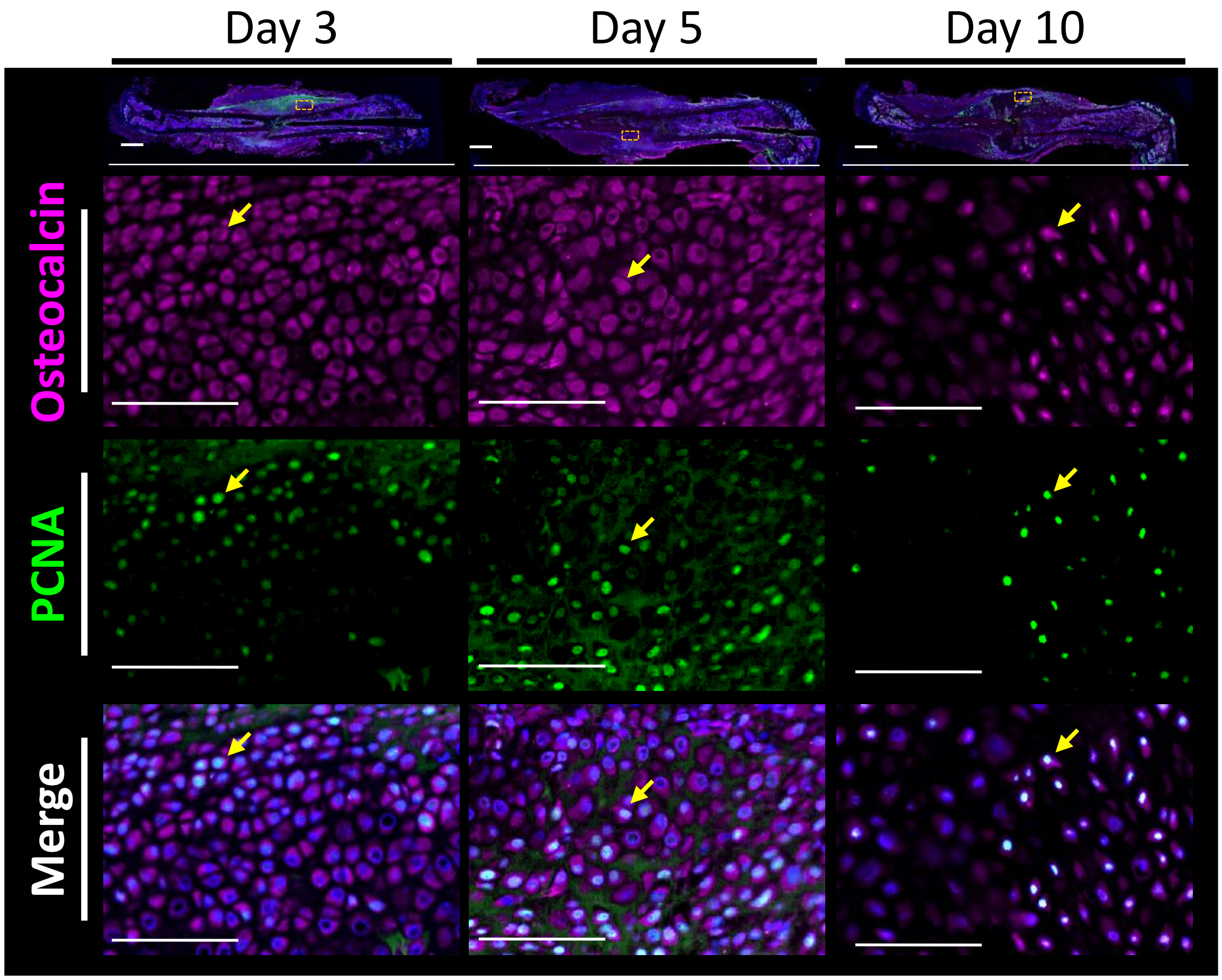

Suppl Figure S10. Bglap/Osteocalcin-expressing callus cells in cartilage regions proliferate during fracture healing.
Representative callus sections from Control mice at days 3, 5 and 10 post-fracture stained by immunofluorescence for Ocn and PCNA. Top row: low-power images showing entire femur. Bottom rows: higher power images of chondrocyte/cartilage region of callus (region outlined by dashed yellow box above). (Scale bars: Low-Power=1 mm, High-power=0.1 mm; Yellow Arrows=Osx+;PCNA+ cells.)

Supplemental Figure S11. Gap Junction Inhibition.

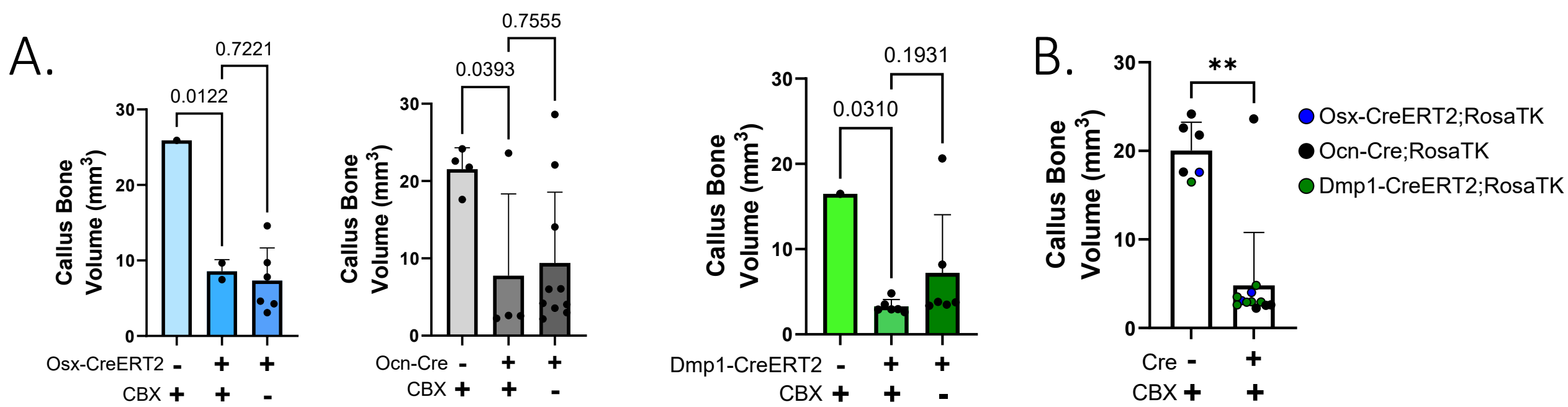

Supplemental Figure S11: Treatment with gap junction inhibitor does not rescue impaired fracture healing in Osx-CreERT2, Ocn-Cre, or Dmp1-CreERT2;ROSA-TK mice 2 weeks post-fracture.
(A) Cre;ROSA-TK mice were treated with GCV and also treated with carbenoxolone (CBX) to block gap junction intercellular communication. In three lines of mice receiving CBX, callus bone volume measured by microCT was significantly less in Cre+ experimental vs. Cre- control. Callus bone volume in Cre+/CBX+ mice was not different from experimental Cre+ mice that did not receive CBX (the latter data also shown in Figs. 4E, 6E, and 8E). **(B)** When data from each Cre line were aggregated to overcome low sample size, Cre+ mice had impaired callus mineralization compared to Cre- mice, both treated with CBX. (Graphs depict mean±SD; statistical differences were determined by One-Way ANOVA with Fisher's LSD Post-Hoc (A) or Mann-Whitney U test (B).)

Supplemental Figure S12. Adq-CreERT2;ROSA-TK mice

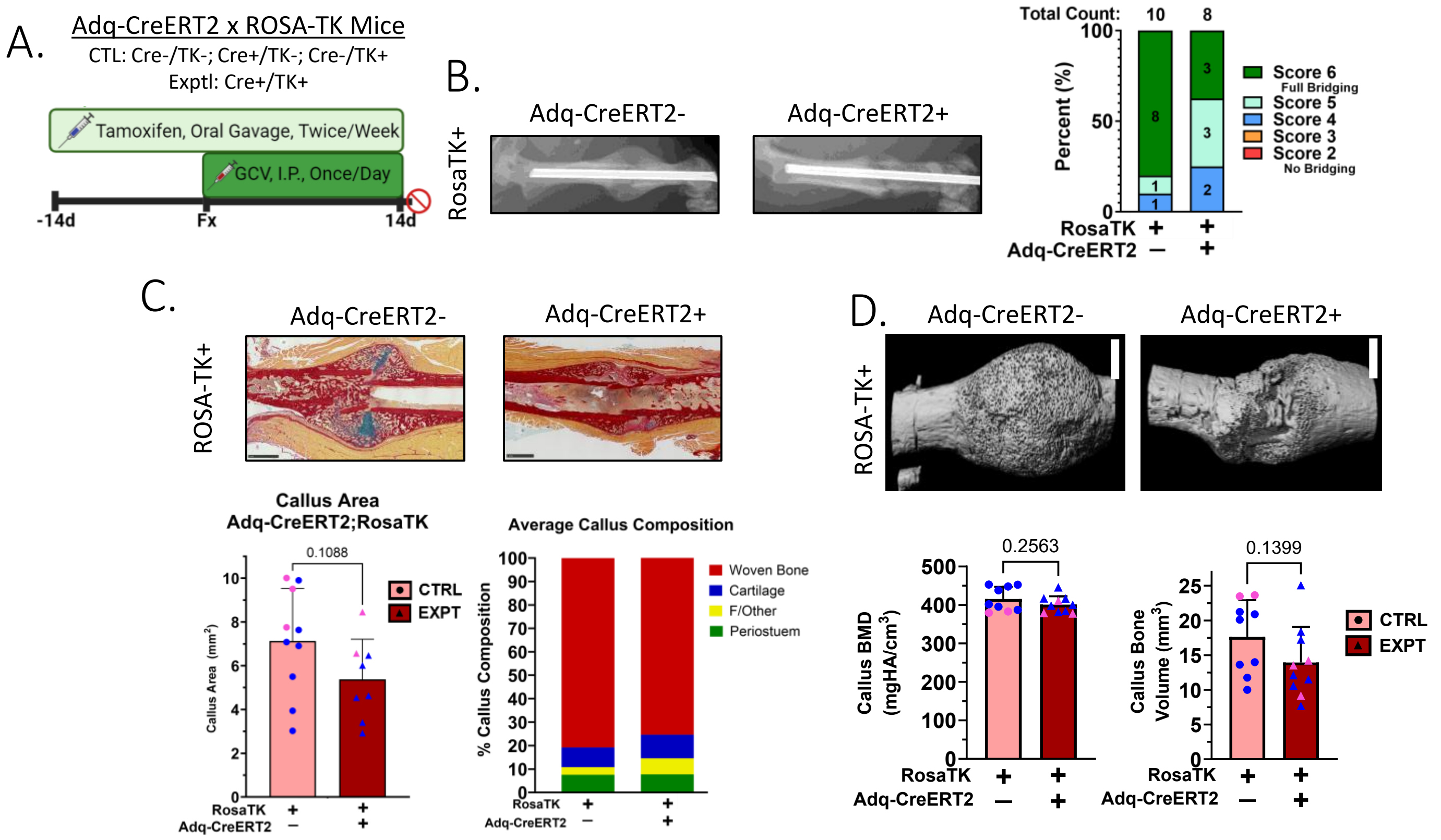

Supplemental Figure S12: Ablation of proliferating adiponectin expressing cells modestly affects fracture healing.

(A) Mice were treated with TMX starting at 10-wks age, followed by femur fracture at 12-wks, followed by 2 weeks GCV treatment. Healing was assessed 14 days after fracture. Control mice were Cre-;TK+ while experimental mice were Cre+;TK+. **(B)** Radiographs showed less mineralized callus in Cre+ mice, resulting in modestly poorer callus bridging scores than in Cre- controls. **(C)** Histological analysis shows a marginally smaller callus area in Cre+ mice, but normal percent cartilage and woven bone compared to Cre- controls. **(D)** Fractured bones were assessed by microCT; there were no significant differences in callus bone volume or BMD between Cre+ mice and control mice. (Statistical differences were determined by Chi-Square test (B) or by two-tailed t-test (C,D). Scale bars: PSRAB=1 mm, TK IHC=0.5 mm, microCT=1 mm.)

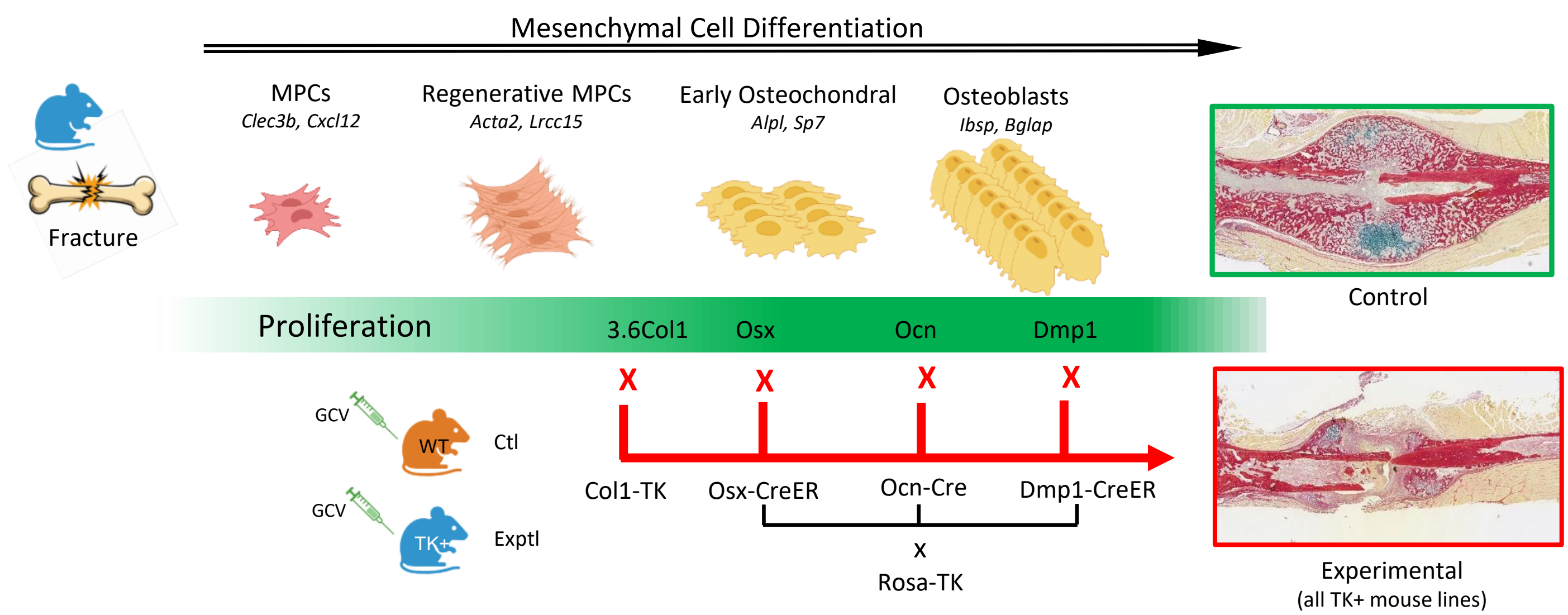

Figure S13: Working Model – Proliferation of Osteolineage Cells is Essential for Fracture Healing. Following fracture, mesenchymal progenitor cells (MPCs) differentiate through several stages, including regenerative MPCs, early osteochondral cells and osteoblasts. Cell proliferation occurs along this path to produce the large numbers of cells needed at each stage of callus formation and maturation. Ablation of proliferating cells that express 3.6Col1, Osx, Ocn or Dmp1 leads to impaired callus formation. Thus, proliferation of cells at each of these osteoblast lineage stages is essential to fracture repair. TK: HSV-Thymidine Kinase; GCV: Ganciclovir. (BioRender and PowerPoint)

Supplemental Figure S14. 5- & 10-Days Post Fracture: IHC Quantification

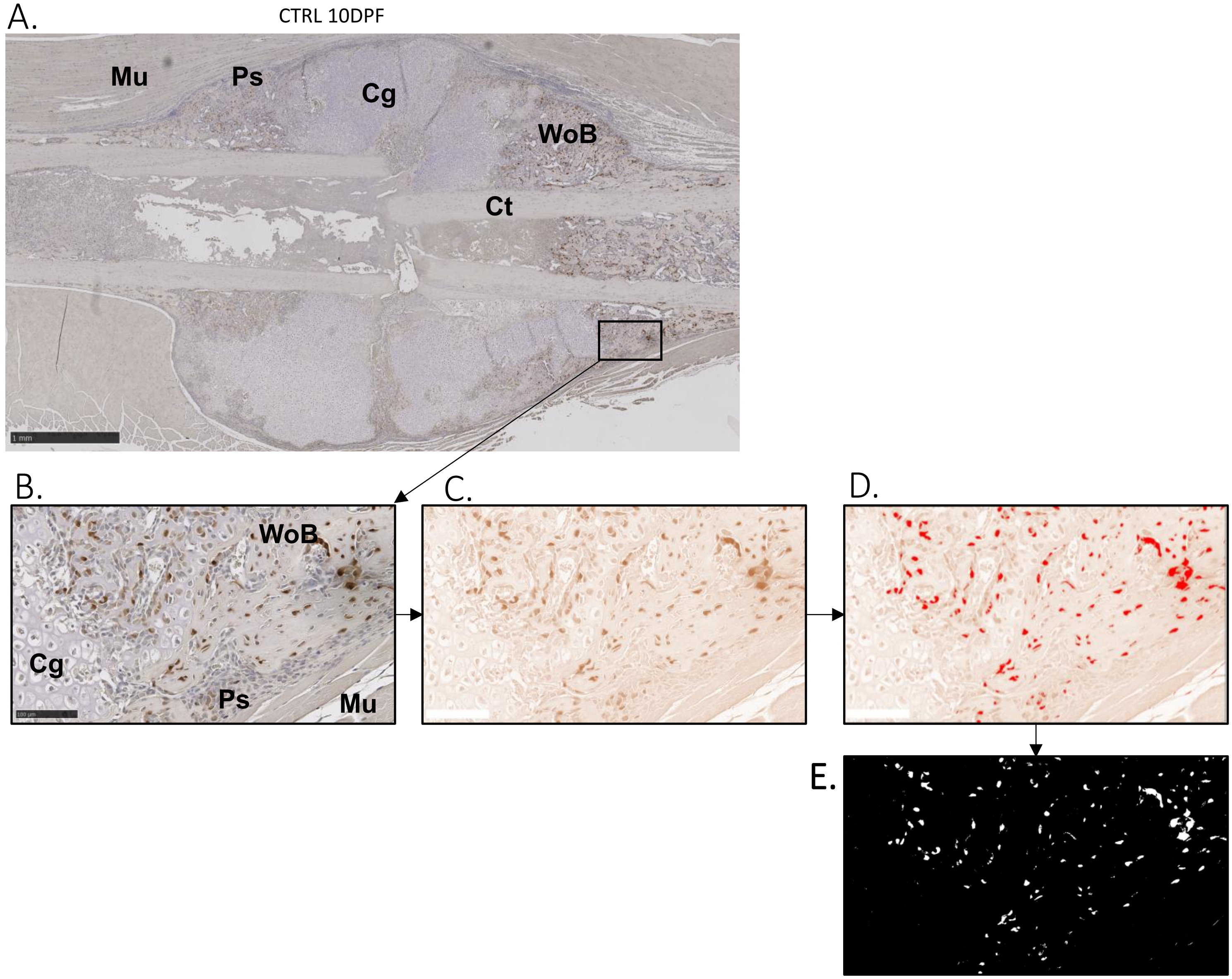

Supplemental Figure S14: Workflow for IHC quantification used for PCNA and TK staining.
(A) Representative image of immunohistochemistry for TK expression in a control sample at 10 days post fracture. (scale bar = 1 mm) **(B)** High magnification field showing woven bone (WoB), cartilage (Cg), periosteum (Ps) and muscle (Mu) tissues in fracture callus adjacent to cortical bone (Ct). (scale bar = 100 µm) **(C)** Isolated channel consisting of DAB signal from (B), **(D)** threshold selected for true positive DAB signal, **(E)** resulting image showing cells with positive DAB signal over the set threshold.

Day 5-All Cells-Top10 DEGs

Cluster 0 : Fibro/Mesench Progenitors	Cluster 1: Fibro/Myofibroblast	Cluster 2: Macrophage	Cluster 3: Endothelial Cells	Cluster 4: Fibroblasts	Cluster 5: Chondro & Pre-Osteoblasts
Igf1	Timp1	Apoe	Fabp4	AY036118	Col2a1
Thbs4	Tnc	C1qa	Ctla2a	Col1a1	Ibsp
Dcn	Tnn	C1qb	Plvap	Col5a1	Col9a3
Abi3bp	Postn	C1qc	Cdh5	Col12a1	Col11a2
Ogn	Tpm2	Hmox1	Pecam1	Col5a2	Acan
Igfbp4	Csrp2	Ctss	Egfl7	Col3a1	Hapln1
Itm2a	Lox	Pf4	Ly6c1	Postn	Col9a1
Mfap4	Loxl2	Ctsb	Col4a1	Fn1	Col11a1
Col14a1	Col5a1	Ccl8	Aqp1	Col1a2	Comp
Mfap5	Thbs2	Ms4a7	Col4a2	(Psme1)	Col9a2

()=downregulated gene

Cluster 6: Neutrophils	Cluster 7: Neutrophils	Cluster 8: Vascular Smooth Muscle	Cluster 9: Skeletal Muscle	Cluster 10: Immune Cells	Cluster 11: Immune Cells	Cluster 12: Neutrophils
Ngp	Il1b	Rgs5	Cdkn1c	H2-Ab1	Saa3	Elane
Camp	Retnlg	Gm13889	Myog	H2-Eb1	Ifitm1	Prtn3
Ltf	Cstdc4	Acta2	Ttn	Cd74	Plac8	Ctsg
Lcn2	Cxcl2	Serpine2	Des	H2-Aa	Ccr2	Mpo
Chil3	Stfa2l1	Ndufa4l2	Igfbp5	Ifitm1	Lyz2	Plac8
S100a8	Csf3r	Tagln	Dag1	S100a4	Wfdc17	Ms4a3
S100a9	Cxcr2	Myh11	Chrna1	H2-DMa	Ly6c2	Fcnb
Wfdc21	Ccl6	Mylk	Tnnt1	Ifi30	Alox5ap	Srgn
Ifitm6	Dusp1	Higd1b	Ppp1r14b	Napsa	Lgals3	Nkg7
Pglyrp1	Srgn	Sparcl1	Spats2l	H2-DMb1	Ms4a6c	Serpinb1a

Cluster 13: Endothelial Cells	Cluster 14: Osteoclasts	Cluster 15: B Lymphocytes	Cluster 16: T Lymphocytes	Cluster 17: Interferon-Stim Fibroblasts	Cluster 18: Endothelial Cells	Cluster 19: Neurons
Ccl21a	Acp5	Igkc	Trbc2	Cxcl9	Itgb2l	Mpz
Cldn5	Atp6v0d2	Ighm	Ms4a4b	Ifit1	Ptprb	Plp1
Mmrn1	Atp6v1b2	Iglc2	Cd3d	Ifit3	Podxl	Mal
Cdh5	Jdp2	Ly6d	Ptprcap	Iigp1	Cldn5	Kcna1
Lyve1	Chchd10	Cd79a	Cd3g	Ifi44	Sox17	Fxyd3
Flt4	Ccl9	Cd74	Trbc1	Ifit3b	Cd177	Cldn19
Pecam1	Slc37a2	Cd79b	Ctsw	Usp18	Adgrl4	Cadm4
Reln	Nfatc1	Iglc1	Lat	Gm4951	Cebpe	Col28a1
Tbx1	Tfrc	H2-Aa	Tespa1	F830016B08Rik	Ly6g	Sfrp5
Egfl7	Pstpip1	Ms4a1	Skap1	Gbp3	Chil1	Sox10

Day 10-All Cells-Top10 DEGs

Cluster 0: Tenocyte-like Fibroblasts	Cluster 1: Chondrocytes	Cluster 2: Hypertrophic Chondrocytes	Cluster 3: Endothelial Cells, Vascular	Cluster 4: Early Chondrocytes, Articular-like	Cluster 5: Smooth Muscle
Aspn	Hapln1	Mmp13	Fabp4	Ucma	Rgs5
Thbs2	Matn3	Col10a1	Ctla2a	Cnmd	Acta2
Tnmd	Col2a1	Spp1	Plvap	Mgp	Tagln
Cthrc1	Col9a1	Rbp4	Pecam1	Mia	Myl9
Ogn	Snorc	Cst3	Cdh5	Ecrg4	Col4a1
Fndc1	Col9a3	Ihh	Egfl7	Matn4	Sparcl1
Abi3bp	Pcolce2	Hpgd	Emcn	Itm2a	Myh11
Tnn	Col27a1	F13a1	Flt1	Peg3	Mylk
Col3a1	Col11a2	Pcsk6	Ly6c1	Col9a1	Tpm2
Timp2	Col9a2	Ddit4l	Ptprb	Col9a3	Gm13889

()=downregulated gene

Cluster 6: Mesenchymal Progenitors	Cluster 7: Macrophages	Cluster 8: Osteoblasts	Cluster 9: Hypertrophic Chondrocytes	Cluster 10: Neutrophils	Cluster 11: Chondrocytes	Cluster 12: Proliferative Cells
Mfap5	Apoe	Ibsp	Spp1	Retnlg	Col11a2	Top2a
Igfbp4	Cd74	Ifitm5	Col10a1	S100a9	Col2a1	Stmn1
Igf1	Ctss	Serpine2	Ibsp	Il1b	Col9a2	Cenpf
Col14a1	C1qa	Sgms2	Cst3	S100a8	Acan	Mki67
Dpt	C1qb	Creb3l1	Hapln1	Cstdc4	Col11a1	Ube2c
Fbn1	Lyz2	Nrp2	(Brd2)	Cxcl2	Col9a3	Birc5
Clec3b	H2-Aa	Cadm1	Snorc	Srgn	(Rplp2)	Prc1
Sfrp2	H2-Eb1	Gpc1	(Dazap2)	Tyrobp	(Rpl24)	Cenpe
Fstl1	Pf4	Pdgfa	(Mcl1)	Stfa2l1	(Rpl30)	Cenpa
Cxcl14	C1qc	Vldlr	(Lamtor4)	Cd52	(Rpl13)	Pclaf

Cluster 13: Neutrophils	Cluster 14: Endothelial Cells, Lymphatic	Cluster 15: T Cells	Cluster 16: Skeletal Muscle	Cluster 17: Proliferative Cells	Cluster 18: Stromal Cells
Camp	Ccl21a	Ccl5	Chodl	Top2a	Sstr2
Ngp	Cldn5	Nkg7	Pax7	Cenpf	Foxs1
Ltf	Mmrn1	Cd52	Myod1	Ube2c	Lrrn3
Lcn2	Egfl7	Ms4a4b	Neb	Prc1	S1pr3
S100a8	Lyve1	Cd69	Drp2	Mki67	Heyl
Chil3	Plvap	Ltb	Cdh15	Pclaf	4833422C13Rik
S100a9	Prox1	H2-Q7	Chrna1	Birc5	Ntn1
Wfdc21	Cdh5	Ptprcap	Jsrp1	Cdk1	Tnfsf11
Ifitm6	Pecam1	Trbc2	Prox1	Cenpe	Gucy1a1
Pglyrp1	Ecscr	Ptprc	Myf5	Tpx2	Ppp1r14c

Day 5-Mesenchymal Cells-Top10 DEGs

**Cluster 0: Fibro 1- Myofibroblasts & Pericytes**	**Cluster 1: Fibro 2 - Mesenchymal Progenitors**	**Cluster 2: Fibro 3- Mesenchymal Progenitors**	**Cluster 3: Fibro 4 - Undefined**	**Cluster 4: Early Osteochondral Cells**	**Cluster 5: Chondrocytes**
Col5a3	Igf1	Ebpl	AY036118	Alpl	Col2a1
Acta2	Abi3bp	Scn7a	Gm42418	Sp7	Hapln1
Tm4sf1	Slpi	Mfap5	(Lamtor5)	*Runx3*	Col9a3
Crip1	Igfbp4	Plaat3	(Erlec1)	Grb14	Col9a1
Tpm2	Sfrp1	Ddah1	(Smap1)	*Pth1r*	Col11a2
Timp1	Hp	Phldb2	(Supt4a)	Acan	Snorc
Actg2	Ecrg4	Kif26b	(Krcc1)	Fmod	Col9a2
Enpp1	*Ogn*	Adamts16	(Ppig)	(Fbln2)	Comp
Tgfbi	Mest	(Acan)	(Bloc1s1)	(Mfap5)	Matn3
Loxl2	*Gas6*	Cxcl12	(Sparc)	Inhba	Acan

()=downregulated gene

Cluster 6:Fibro 5 - Mesenchymal Progenitors	Cluster 7: Chondro/Undefined	Cluster 8: Interferon-Stimulated Fibroblasts	Cluster 9: Fibro 6 - Tenocytes & Fibrocartilage	Cluster 10: Proliferating Cells	Cluster 11: Peripheral Nerve
Eln	Retnlg	Isg15	Thbs4	Top2a	Mpz
Cxcl14	Lcn2	Bst2	Tnmd	Hist1h1b	Mbp
Cygb	S100a8	Ifit1	Chad	Mki67	Gatm
Serping1	S100a9	Cxcl9	Mgp	Ube2c	Plp1
Gdf10	Cd52	Ifit3	Abi3bp	Pclaf	Mal
Clec3b	Tyrobp	Ifitm3	Fmod	Prc1	Kcna1
Selenop	Lyz2	Ly6e	Cilp	Birc5	Fxyd3
Cfb	Cybb	Iigp1	Cilp2	Cenpf	Egfl8
C1s1	Gmfg	Oasl2	Apod	Smc2	Cldn19
Pi16	Rac2	Stat1	Chodl	Cenpe	Mt3

Day 10-Mesenchymal Cells-Top10 DEGs

Cluster 0: Chondro1- Mature Chondrocytes	Cluster 1:Fibro1-Tenocyte-like	Cluster 2:Hypertrophic Chondro1- Mature Hypertrophic Chondrocytes	Cluster 3:Chondro2-Early Chondrocytes	Cluster 4:Fibro2- Matrix Fibroblasts
Hapln1	Tnmd	Col10a1	Ucma	Ecm1
Matn3	Abi3bp	Mmp13	Cnmd	Tnn
Ppa1	Aspn	Cst3	Mgp	Crip1
Col9a1	Thbs4	Ihh	Mia	Aebp1
Col2a1	Fbn2	Spp1	Matn4	Postn
Sgms2	Ogn	Rbp4	Ecrg4	Col6a3
Col27a1	Mfap4	F13a1	Itm2a	Angptl2
Col9a3	Igf1	Hpgd	Peg3	*Tnc*
Col11a2	Lsp1	Ddit4l	H19	Col5a1
Snorc	Fndc1	Pcsk6	Scrg1	Inhba

()=downregulated gene

Cluster 5: Osteoblastic	Cluster 6:Chondro3-Proliferating	Cluster 7:Fibro3-Mesenchymal Progenitors	Cluster 8:Quiescent Chondrocytes	Cluster 9: Fibro4- Mixed/Reparative	Cluster 10: Unidentified	Cluster 11: Unidentified
Ifitm5	Col10a1	Mfap5	(Rplp2)	Gzme	Gm42418	Hp
Nrp2	AY036118	Clec3b	(Fau)	Cxcl12	AY036118	Chil1
Serpine2	(Auts2)	Pi16	(Rpl24)	Cxcl5	Lars2	Lcn2
Pdgfa	(Gaa)	Col14a1	(Rps18)	Fgf7	(Ube3a)	Lbp
Creb3l1	Spp1	Ly6a	(Rpl13)	C3	(Brd3)	Mmp3
Tmem119	(Hoxc10)	Serping1	(Rpl30)	Gas1	(Bclaf1)	Mt2
Serpinb6b	(Ttc28)	Fbn1	(Rack1)	Il1r1	(Tnrc6b)	C3
Ptprz1	Gm42418	Dpt	(Rpl5)	Cyp1b1	(Kdm2a)	Fth1
Gpc1	(Ski)	Igfbp4	(Rpl37a)	Ptgfr	(Rp9)	Steap4
Cadm1	(Scarf2)	Cilp	(Rps23)	Brinp3	(Senp6)	Spp1
